# Statistical characterization of therapeutic protein modifications

**DOI:** 10.1038/s41598-017-08333-y

**Published:** 2017-08-11

**Authors:** Tsung-Heng Tsai, Zhiqi Hao, Qiuting Hong, Benjamin Moore, Cinzia Stella, Jeffrey H. Zhang, Yan Chen, Michael Kim, Theo Koulis, Gregory A. Ryslik, Erik Verschueren, Fred Jacobson, William E. Haskins, Olga Vitek

**Affiliations:** 1 Northeastern University, 360 Huntington Avenue, Boston, MA 02115 USA; 2 Protein Analytical Chemistry, Genentech, 1 DNA Way, South San Francisco, CA 94080 USA; 3 Nonclinical Biostatistics, Genentech, 1 DNA Way, South San Francisco, CA 94080 USA; 4 Protein Chemistry, Genentech, 1 DNA Way, South San Francisco, CA 94080 USA; 5 Eurofins Lancaster Laboratories, 2425 New Holland Pike, Lancaster, PA 17605 USA

## Abstract

Peptide mapping with liquid chromatography–tandem mass spectrometry (LC-MS/MS) is an important analytical method for characterization of post-translational and chemical modifications in therapeutic proteins. Despite its importance, there is currently no consensus on the statistical analysis of the resulting data. In this manuscript, we distinguish three statistical goals for therapeutic protein characterization: (1) estimation of site occupancy of modifications in one condition, (2) detection of differential site occupancy between conditions, and (3) estimation of combined site occupancy across multiple modification sites. We propose an approach, which addresses these goals in terms of summarizing the quantitative information from the mass spectra, statistical modeling, and model-based analysis of LC-MS/MS data. We illustrate the approach using an LC-MS/MS experiment from an antibody-drug conjugate and its monoclonal antibody intermediate. The performance was compared to a ‘naïve’ data analysis approach, by using computer simulation, evaluation of differential site occupancy in positive and negative controls, and comparisons of estimated site occupancy with orthogonal experimental measurements of N-linked glycoforms and total oxidation. The results demonstrated the importance of replicated studies of protein characterization, and of appropriate statistical modeling, for reproducible, accurate and efficient site occupancy estimation and differential analysis.

## Introduction

Therapeutic proteins, also known as biologics (e.g., monoclonal antibodies, enzymes, or receptor modulators), are an important class of medicines with more than 70 marketed products and $125B in worldwide sales projected for 2020^1–3^. Therapeutic proteins are manufactured using well-controlled processes and comprehensively characterized with a variety of biophysical and functional (e.g., cell-based bioassay) methods to ensure the “totality of evidence”^4, 5^, consistent product quality, safety and efficacy for patients^6, 7^.

Peptide mapping with liquid chromatography–tandem mass spectrometry (LC-MS/MS) is one of the most important analytical methods for therapeutic protein characterization. It relies on peptides generated by enzymatic or chemical cleavage of the protein^8–13^, and provides amino acid residue-specific information. It allows confirmation of the amino acid sequence, and to identify and quantify various *peptide forms*, i.e., combinatorial post-translational and chemical modifications of peptides. Examples of modifications that can be characterized by LC-MS/MS include oxidation of methionine and tryptophan, glycosylation of asparagine, and linker-drug modifications to cysteine.

LC-MS/MS peptide mapping identifies *peptide features* (i.e., peptide ions with various charge states, isotopes and modifications) in the mass-to-charge (*m*/*z*) and chromatographic (i.e., retention time) dimensions. Modifications to specific amino acid residues (*sites*) are identified by characteristic shifts in *m*/*z* (e.g., a +15.995 Da mass shift for methionine oxidation) and/or retention times relative to the unmodified peptides. The peptide features are quantified by integrating the chromatographic peaks. This quantitative information allows us to characterize the extent of various modifications in a specific condition in terms of *site occupancy*, defined as the percent relative abundance of a specific modification of an amino acid (i.e., *site*) among all its modified or unmodified states. For example, 5% oxidation of a particular methionine residue in a peptide might reflect a 1/(1 + 19) ratio of the integrated chromatographic peak area from the single-oxidized peptide’s feature, to the sum of the peak areas from all the modified and unmodified forms of that peptide. Site occupancy calculations are essential for defining and controlling manufacturing specifications of therapeutic proteins.

Translated into statistical language, the goals of therapeutic protein characterization are (1) the objective *estimation of site occupancy* for a specific condition; (2) the determination of *differential site occupancy* (i.e., systematic changes in site occupancies between conditions)^14^; and (3) the objective *estimation of combined occupancy* over multiple sites for comparison with orthogonal evidence. Despite the importance of this goal, there is currently no consensus on how to appropriately summarize the available quantitative information from LC-MS/MS spectra, and to carry out these goals. *Ad-hoc* data analysis methods are typically used. For example, site occupancy is often quantified with the sum of the feature intensities of a form at a site, divided by the sum of all the feature intensities across all the forms at that site. A subjectively chosen subset of the most abundant and/or high-confidence features can be used. When replicate LC-MS/MS runs are available, detection of differential site occupancy is often done with a *t*-test. To save time and cost, many experiments are unreplicated, and in this case changes in site occupancy between conditions are often claimed significant when they exceed a heuristic cutoff.

Although simple, the *ad-hoc* methods lack statistical justification. Their statistical properties, including robustness to interferences and missing values, are unknown. In this manuscript we reconstruct the statistical model and the assumptions underlying a common *ad-hoc* approach (naïve approach), and highlight its deficiencies. We also propose an alternative statistical approach (proposed method), which explicitly characterizes issues arising in practical applications. It enables automatic data processing and summarization, highlights the role of replicated studies, and improves the reproducibility and the accuracy of the results.

The naïve and proposed approaches were evaluated using a case study of modifications for a cysteine-conjugated antibody-drug conjugate (ADC) and its monoclonal antibody intermediate (AI). The samples, stored at −70 °C until analysis, were characterized under control (i.e., reference) conditions and under forced degradation conditions such as 2,2′-Azobis(2-amidinopropane) dihydrochloride (AAPH). The experimental design included additional differential site occupancy controls for evaluating the statistical methodology, in particular negative controls for the differential site occupancies of non-linker-drug-modified peptides in ADC versus AI reference samples and positive controls for the differential site occupancies of AI (or ADC) forced degradation samples versus AI (or ADC) reference samples as described below. For site occupancy estimation, selected reference and forced degradation samples were also characterized with orthogonal, non-mass spectrometric methods. These included N-linked glycosylation using 2-aminobenzamide hydrophilic interaction liquid chromatography (2-AB HILIC) and capillary electrophoresis-laser induced fluorescence (CE/LIF)^15–18^, and total oxidation measurements of Fab and Fc fragments using a liquid chromatography-ultraviolet absorption (LC/UV) IdeS assay^19^. Although these orthogonal methods generally lack site-specific resolution, agreement with quantification was used as external evidence of the accuracy of summaries from peptide mapping.

Non-linker-drug-modified peptides in AI and ADC reference samples were used as negative controls for differential site occupancy determination. These reference samples shared dozens of post-translational modifications (as in Supplementary Table S1), such as oxidized methionine or tryptophan residues and N-linked glycosylation, and we expected no changes in site occupancy except for the intentional conjugation-induced modifications in linker-drug-modified peptides. In contrast, we expected differences among reference samples versus forced degradation or enriched minor variant samples. For example, significant increases in site occupancy were expected for oxidized methionine and tryptophan residues in 3 mM AAPH forced degradation samples. Intentional linker-drug modifications of reactive cysteine residues (e.g., the linker-drug and its hydrolysis products) were only considered for the ADC.

We compared the proposed statistical approach to a non-statistical naïve data analysis method that is frequently used, by using computer simulation, comparisons with orthogonal experimental measurements, and evaluation of performance in detecting differential site occupancy. Compared to the naïve approach, the proposed approach improved the accuracy and the efficiency of site occupancy estimation, resulted in a better calibrated type I error rate, and improved statistical power of detecting differential site occupancy. The proposed method reduced the negative impact of missing values and outlying measurements, and improved the efficiency of site occupancy estimates in such circumstances. It improved the sensitivity and the specificity of detecting differential site occupancies. Under the scenarios of small sample sizes that are frequently encountered, the proposed method improved the overall performance for the analysis.

## Results

### Experimental design

**Figure 1:**
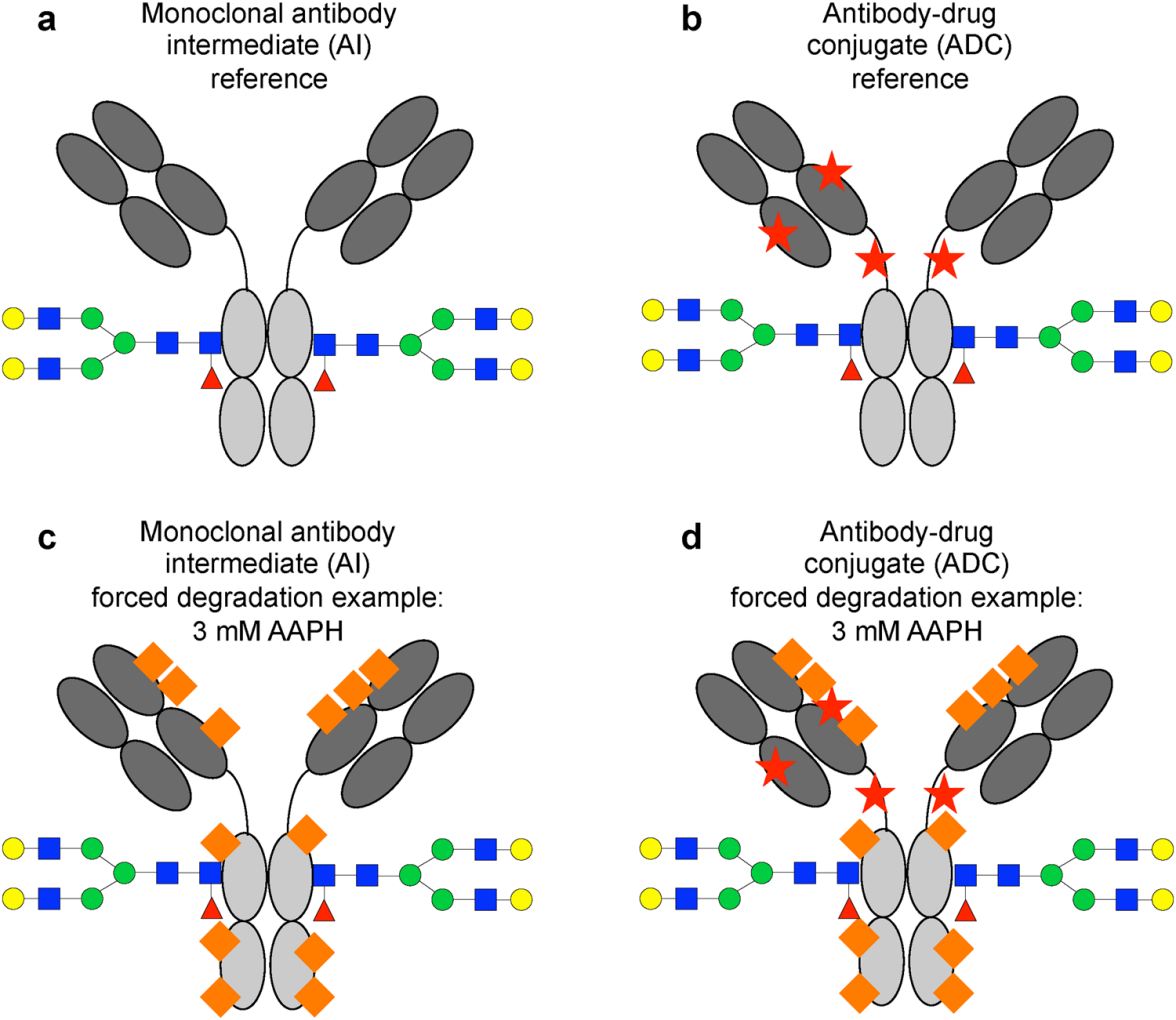
Overall experimental design. (**a**) Schematic representation of a reference antibody intermediate (AI) sample (gray), and its modifications such as N-linked glycosylation (colors). (**b**) Schematic representation of a reference antibody drug conjugate (ADC). The ADC is an immunoglobulin G1-kappa monomethylauristatin E (MMAE) conjugate, where one or more of the inter-chain disulfide bridges of the AI were intentionally chemically modified to an average of three to four linker-drug-modified cysteine residues (red stars). The conjugation was not expected to impact post-translational modifications of the AI. More than 34 post-translational modifications were considered herein, and enriched minor variant samples were generated for some modifications. (**c**) Schematic representation of one of the forced degradation conditions (e.g., 3 mM AAPH) applied to AI. The forced degradation introduces modifications such as oxidation (orange rectangles). (**d**) Schematic representation of one of the forced degradation conditions (e.g., 3 mM AAPH) applied to ADC. As in AI, forced degradation introduces modifications such as oxidation (orange rectangles). The Fab (dark gray) and Fc (light gray) regions of the molecules are shown for clarity.

The experimental design is outlined in Fig. 1. Figure 1a and b are schematic representations of the AI and of the ADC. The ADC was an immunoglobulin G1 monomethylauristatin E (MMAE) conjugate, where one or more of the inter-chain disulfide bridges of the AI were chemically reduced prior to conjugation with a linker-drug. Out of eight reactive cysteines, on average three to four were intentionally modified with a linker-drug via a thioether bond for an average drug-to-antibody ratio of 3.5. MMAE conjugation was performed with a cleavable maleimidecaproyl-valyl-citrullinyl-p-aminobenzylcarbamate (mc-val-cit-PABC) linker. Figure 1c and d schematically represent examples of forced degradation conditions applied to the AI and ADC (e.g., stressed with 3 mM AAPH). More than eight different forced degradation conditions were applied to the AI and ADC. Moreover, enriched minor variant samples were generated via preparative chromatographic and electrophoretic methods for particular modifications (data not shown).

To evaluate the ability of the proposed statistical method to accurately estimate site occupancy (*Goal 1*), experiments were also conducted using orthogonal non-mass spectrometric methods as an external benchmark of performance. These included the analyses of N-linked glycosylation with 2-AB HILIC and CE/LIF of AI and ADC reference samples (see Supplementary Materials).

To evaluate the performance of the statistical approach in detecting differential site occupancies (*Goal 2*), three pairs of conditions were evaluated. First, the AI and the ADC reference samples were compared (Fig. 1a and b). Except for the intentional linker-drug modifications, the conjugation was not expected to impact post-translational modifications of the AI such as N-linked glycosylation. Second, the AI reference samples were compared to the AI samples under stress (Fig. 1a and c). The stresses were expected to introduce modifications such as oxidative modifications. Third, the ADC reference samples were compared to the ADC samples under forced degradation conditions or enriched minor variant samples (Fig. 1b and d), which were also expected to introduce modifications such as oxidative modifications (data not shown). The information on the expected modifications allowed evaluation of the proposed statistical method in terms of sensitivity and specificity of detecting differential site occupancies. Comparison between AI and ADC under stress was not performed, as the stresses had to be performed under different conditions (e.g., formulation buffers) for AI versus ADC.

To estimate the combined site occupancy (*Goal 3*), an orthogonal method, LC/UV IdeS assay was used to quantify total oxidation in AI and ADC reference samples and forced degradation samples. This method is not site-specific and in order to use it as an external benchmark, estimation of combined occupancy of the oxidative forms over multiple sites is needed for the comparison.

### Results of LC-MS/MS peptide mapping

**Figure 2:**
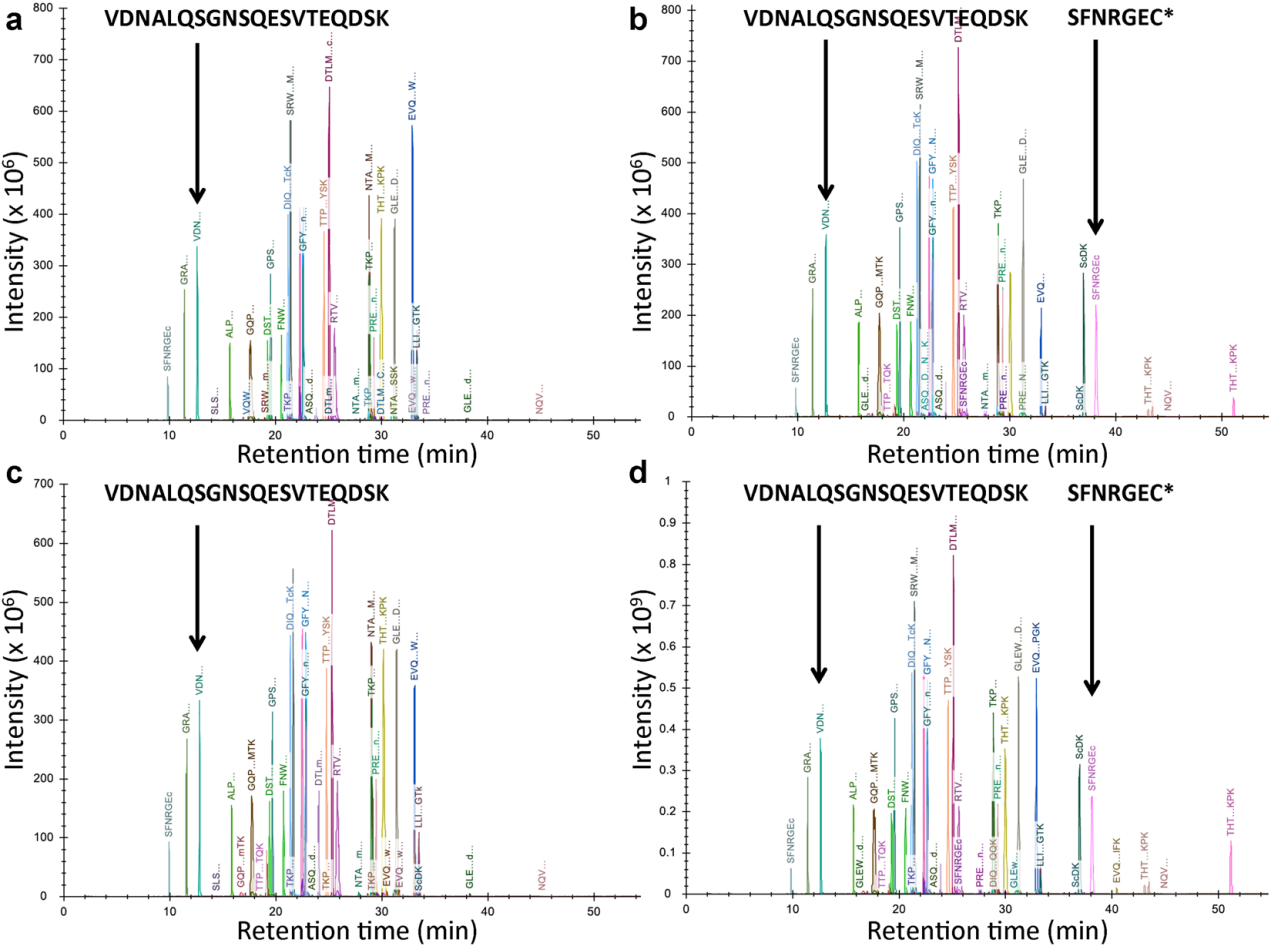
Representative extracted ion chromatograms for peptides from (**a**) AI and (**b**) ADC reference samples, and (**c**) AI and (**d**) ADC forced degradation (e.g., 3 mM AAPH) samples. Most peptides, including the unmodified peptide VDNALQSGNSQESVTEQDSK that can be used for normalization, were unaffected by the conjugation of linker drug. On the other hand, the linker-drug-modified peptides such as SFNRGEC were only observed in the ADC. In the ADC, the cysteine in this peptide was observed with carboxymethylation (+58 Da), vcMMAE- (+1315 Da), and vcMMAEhydro (+1333 Da) modifications. In the AI, the cysteine was fully carboxymethylated and no linker-drug modifications were observed. Comparisons of non-linker-drug modifications between reference samples provided negative control for differential site occupancy analysis while comparisons between reference samples versus their forced degradation samples provided positive controls.

The AI and ADC samples, and their stresses, were enzymatically digested, randomized, and analyzed with 3–6 replicates using LC-MS/MS. Figure 2 shows representative peptide mapping data for AI and ADC samples. All unmodified cysteines were carboxymethylated [+58 Da] during sample preparation prior to LC-MS/MS-based peptide mapping analysis. Most peptides, including the unmodified peptide VDNALQSGNSQESVTEQDSK shown in Fig. 2 and Supplementary Fig. S1 and the N-linked glycopeptides shown in Supplementary Fig. S2, were unaffected by the conjugation of linker drug, as expected. Consequently, their relative intensities appeared unchanged and these peptides were negative controls for the differential site occupancies of AI versus ADC. The peptide VDNALQSGNSQESVTEQDSK was used for normalization, as described below. In contrast, the linker-drug-modified peptides such as SFNRGEC shown in Fig. 2b and d were only observed in the ADC. Its hydrophobic vcMMAE- and vcMMAEhydro-modified forms have an increased retention time relative to the fully carboxymethylated form of this peptide (the only form observed in the AI), where late-eluting chromatographic peaks are a clear indication of the ADC. In contrast, comparison of Fig. 2a versus 2c (or Fig. 2b versus 2d) revealed positive controls for the differential site occupancies of AI (or ADC) reference samples versus AI (or ADC) forced degradation samples (e.g., 3 mM AAPH), such as the oxidized peptides shown in Supplementary Fig. S3 (with expanded views in Supplementary Fig. S4). The monoisotopic mass shifts and molecular formulas of characteristic modifications are shown in Supplementary Table S1, including modifications observed at low (e.g., <1%) levels (or not at all) in reference samples, as well as modifications observed at high levels (e.g., >1%) in forced degradation samples (e.g., 3 mM AAPH). Characteristic peptides and modified amino acid residues are shown in Supplementary Table S2 while the entire list of identified peptides with and without modifications is provided in Supplementary Materials (Dataset 1).

### Practical data analysis with Skyline and R

Skyline^20^ was used to detect and quantify the chromatographic peaks in the LC-MS and LC-MS/MS profiles. The selected peaks were verified manually and problematic peaks were corrected or removed as appropriate as described in the Methods Section (chromatography-based quantification). After the verification, a report dataset was exported for statistical analysis in R. In the dataset, a peptide feature for each run is defined as a combination of charge state, isotope and modification, and quantified by the area under the chromatographic peak (the quantity is referred to as *feature intensity* henceforth). Overall the dataset contains 32 sites, 42 peptides, 102 modified peptides, and 793 peptide features. To characterize a site in terms of the occupancies of different forms, intensities of peptide features were used to infer the underlying abundances of the forms. If a peptide is modified at multiple sites, the observed feature intensity cannot be attributed to a form at one of the modification sites in full certainty. Therefore, in this study characterization of site occupancy for a particular site was based on peptides only modified at that site (except for an additional carboxymethylated cysteine) to avoid the confounding effect due to other modifications. Focus was given to the characterization at 30 sites with 73 forms, represented by 778 peptide features.

**Figure 3:**
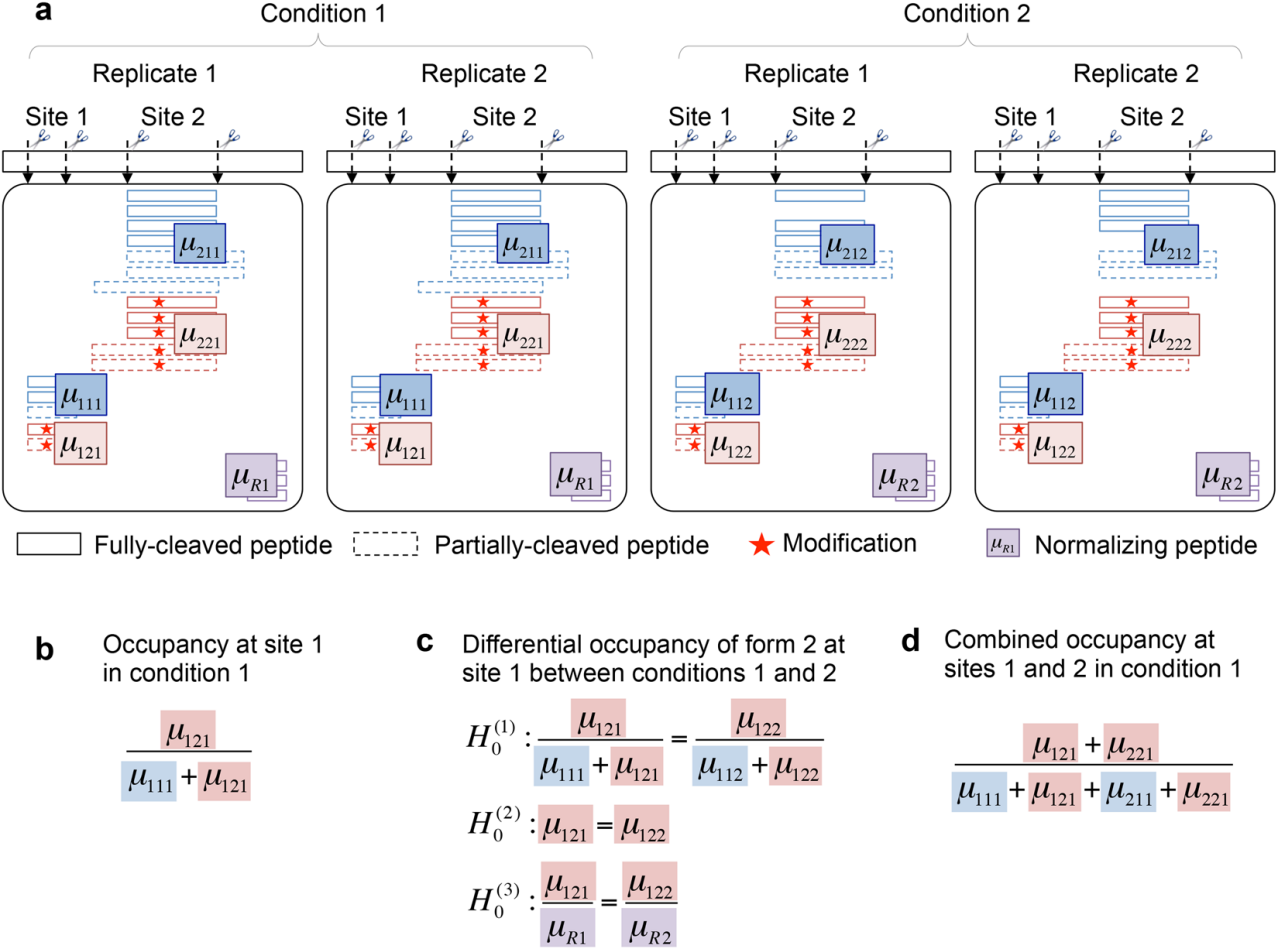
Goals of statistical characterization of therapeutic proteins. (**a**) Schematic representation of the data, in a simplified case of two conditions, two replicate runs, two sites and two forms. A form in each site is quantified by multiple spectral features (boxes), which are charge states and isotopes of a peptide. The feature intensities are viewed as repeated measurements of the underlying abundance of the form, where the abundance of site *i*, form *j*, and condition *k* is denoted by μ_*ijk*_. Peptides can be fully cleaved (solid lines) and/or partially cleaved (a.k.a., mis-cleaved, dashed lines). Some spectral features can be missing. (**b**) Goal 1: statistical characterization of site occupancy estimates the quantities underlying a peptide feature in each condition, replicate, site and form. (**c**) Goal 2: statistical characterization of differential site occupancy tests the null hypothesis of *no change* against the alternative. The hypotheses differ in the use of the normalizing factor in the denominator. (**d**) Goal 3: as in (**b**), but combining the quantitative information across multiple sites.

Figure 3a schematically illustrates a simplified version of the resulting data structure, to be used to address the goals in therapeutic protein characterization. The figure illustrates the multiple layers of variation present in the experiment. A site is characterized by a set of modification forms, each represented by multiple peptide features. The features vary in sequence (e.g., fully or partially cleaved peptides), ionization efficiency, charge states, isotopes, etc. The number and type of features vary across the sites and the forms, and also across replicate LC-MS/MS acquisitions of a same sample, and across conditions.

### Implementation of the naïve approach

The downstream data analysis must summarize all the available quantitative information. A frequently used method for doing this, which we refer to as the *naïve approach*, quantifies site occupancy in a single LC-MS run by summing all the feature intensities of a form at a site, and dividing them by the sum of all the feature intensities across all the forms at that site (*Goal 1*). Replicated studies were considered, and detection of differential site occupancy with a *t*-test was implemented, taking as input the calculated site occupancy of a form in a run (*Goal 2*). When it is necessary to compare the results with evidence from other experiments lacking site-specific resolution, combined occupancy is quantified in a single run by summing all the feature intensities of a modification over sites, and dividing them by the sum of all the feature intensities across all the forms at the considered sites (*Goal 3*). Supplementary Section S3 explicitly states the model and the assumptions that are implicitly assumed by the naïve approach. We argue below that this naïve approach is inadequate, because it can lead to bias (i.e., systematic mistakes in estimation of site occupancies, or their changes between conditions), inefficiencies (i.e., larger than necessary uncertainty associated with the summaries), or both.

### Proposed statistical approach for therapeutic protein characterization

The proposed approach is described informally below, and a more formal description is provided in Supplementary Sections S4–S6.

Figure 3a illustrates the quantity of interest for the three goals in therapeutic protein characterization. In the proposed approach, the LC-MS features are viewed as repeated measurements of the underlying abundance of the form, which is denoted by μ. The underlying site occupancy is therefore a combination of these quantities for all of the forms, as shown in Fig. 3b (*Goal 1*).

To detect systematic changes in site occupancies between conditions (*Goal 2*), Fig. 3c shows that there are several ways to define differential site occupancy as function of μ. These include changes in the form on the scale of its site occupancy (Hypothesis *H*_0_^(1)^), on the scale of abundance (Hypothesis *H*_0_^(2)^), or on a scale that relates the estimates of the abundance with respect to peak intensity of a peptide that is not subjected to modifications, and has a constant form across all conditions of interest (Hypothesis *H*_0_^(3)^). Hypothesis *H*_0_^(1)^ relates directly to the quantity of interest, i.e., difference in site occupancy, and the associated uncertainty is characterized based on the estimates of abundances for all forms at the site. Hypotheses *H*_0_^(2)^ and *H*_0_^(3)^, on the other hand, are only based on the form being tested, regardless of the status of the other forms. The two hypotheses differ in the use of reference peptide. Testing based on *H*_0_^(2)^ is the same as in relative label-free quantitative proteomics, while testing based on *H*_0_^(3)^ is the same as in relative label-free quantitative proteomics with a global reference standard.

The estimation of combined occupancy (*Goal 3*) requires the integration of the abundances over multiple sites, quantified by peptide features with different amino acid residues, as illustrated in Fig. 3d. This summarization makes a critical assumption of identical ionization efficiency for the peptide features across different sites. This assumption may not be verified in practice, and as a result only an approximate agreement of combined site occupancy estimation between LC-MS/MS based peptide mapping and alternative experiments that do not have site-specific resolution can be expected.

When all the features are reliably observed in every form and run, the *ad-hoc* methods for data summarization and analysis may suffice. In real-life experiments, however, missing values, interferences and noise arise. We explicitly recognize such issues in practical applications, and propose an automated data processing and summarization procedure. To avoid the confounding due to different ionization efficiencies of features, we automatically select a subset of the LC-MS features with consistent coverage in runs and forms. To reduce the negative impact of missing values and outlying measurements, we perform imputation of censored missing values and robust summarization of the retained features using Tukey’s median polish (TMP). The details of summarization are described in Supplementary Section S4.1.

After the summarization, estimates of the underlying abundance of a form μ and measures of associated uncertainty are derived using a family of linear mixed models. The remaining quantities of interest (e.g., site occupancy), are then obtained by non-linear transformation (Supplementary Sections S4–S6).

### Computer simulation

**Figure 4:**
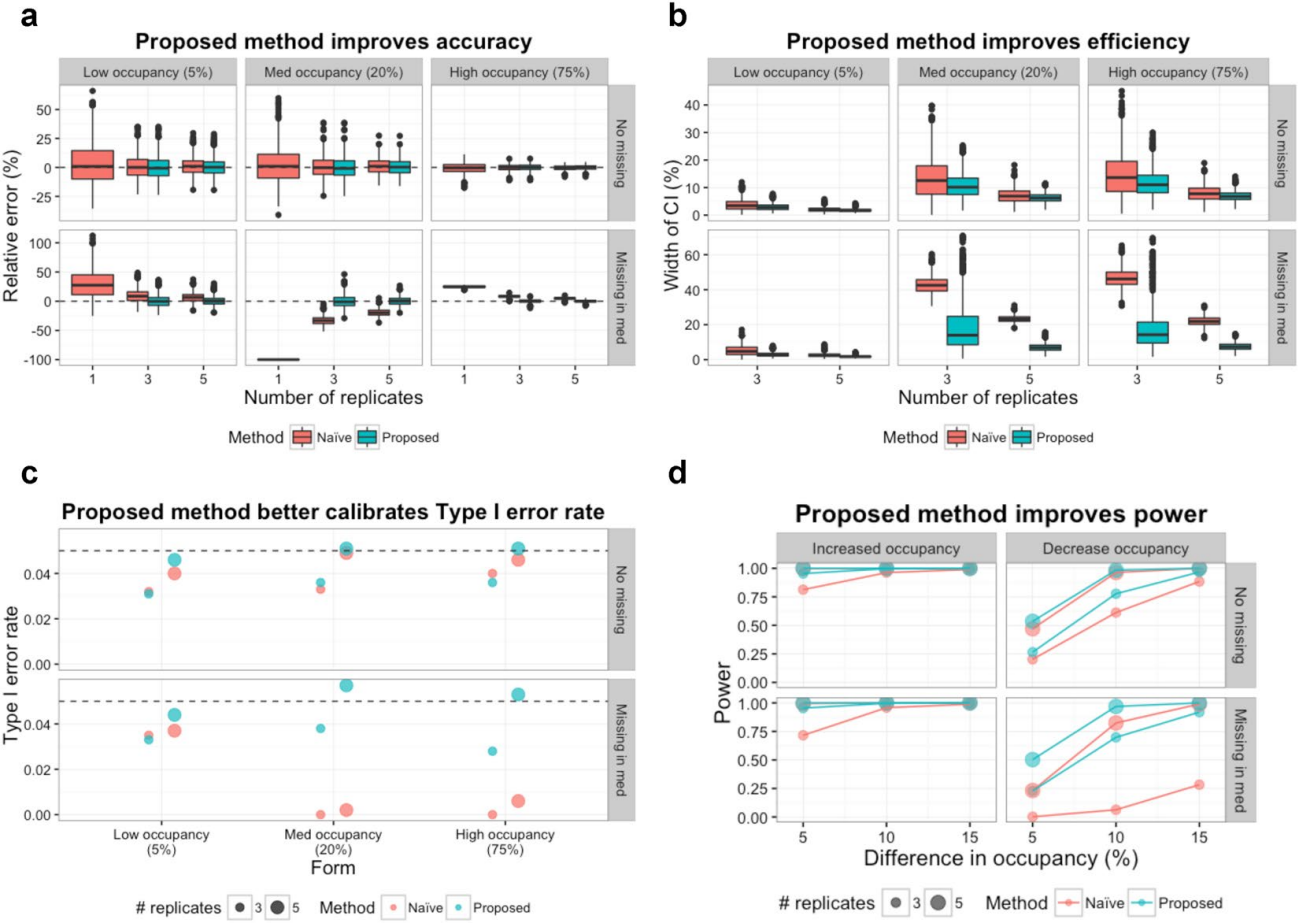
Simulation analysis for site occupancy estimation and detection of differential site occupancy. A scenario of three forms with occupancy of 5%, 20% and 75% for each is considered, where mean and standard deviation of log2-intensity are set to 25 and 0.2, respectively. The results by the naïve and proposed methods are compared based on 1000 simulations. (**a**) Relative errors of site occupancy estimates (i.e., estimation error divided by the true value), with different number of replicates and presence of missing data. (**b**) Widths of the 95% confidence intervals. (**c**) Type I error rate based on a statistical significance level set at 0.05. The horizontal dashed lines indicate the theoretical Type I error. The dots indicate the actual Type I error across 1000 simulations. (**d**) Statistical power to detect differential site occupancies with differences of 5%, 10% and 15%.

The proposed statistical approach for site occupancy estimation (*Goal 1*) and detection of differential site occupancy (*Goal 2*) was evaluated and compared with the naïve approach using computer simulation. We considered a scenario of 3 forms of a site, with occupancies of 5%, 20% and 75%. To highlight the role of the statistical analysis, we did not consider alternative approaches to feature intensity summarization, and simulated the summarized intensities for each form. The effect of missing peaks was investigated by removing a summarized intensity from one replicate of a form of low, medium or high occupancy. The results are summarized in Fig. 4. The proposed method improved the accuracy (i.e., relative error) and efficiency (i.e., the width of confidence intervals) of site occupancy estimation as compared to the naïve approach (Fig. 4a and b). For differential site occupancy determination, the proposed method reported experimental Type I error that is closest to the theoretical values, and maximized the power of detecting differential site occupancy (Fig. 4c and d). The improvements were particularly apparent in simulations with fewer replicates, and in presence of missing values. Additional details of the simulation and results are provided in Supplementary Section S7.

### Experimental data, Goal 1: site occupancy estimation

**Figure 5:**
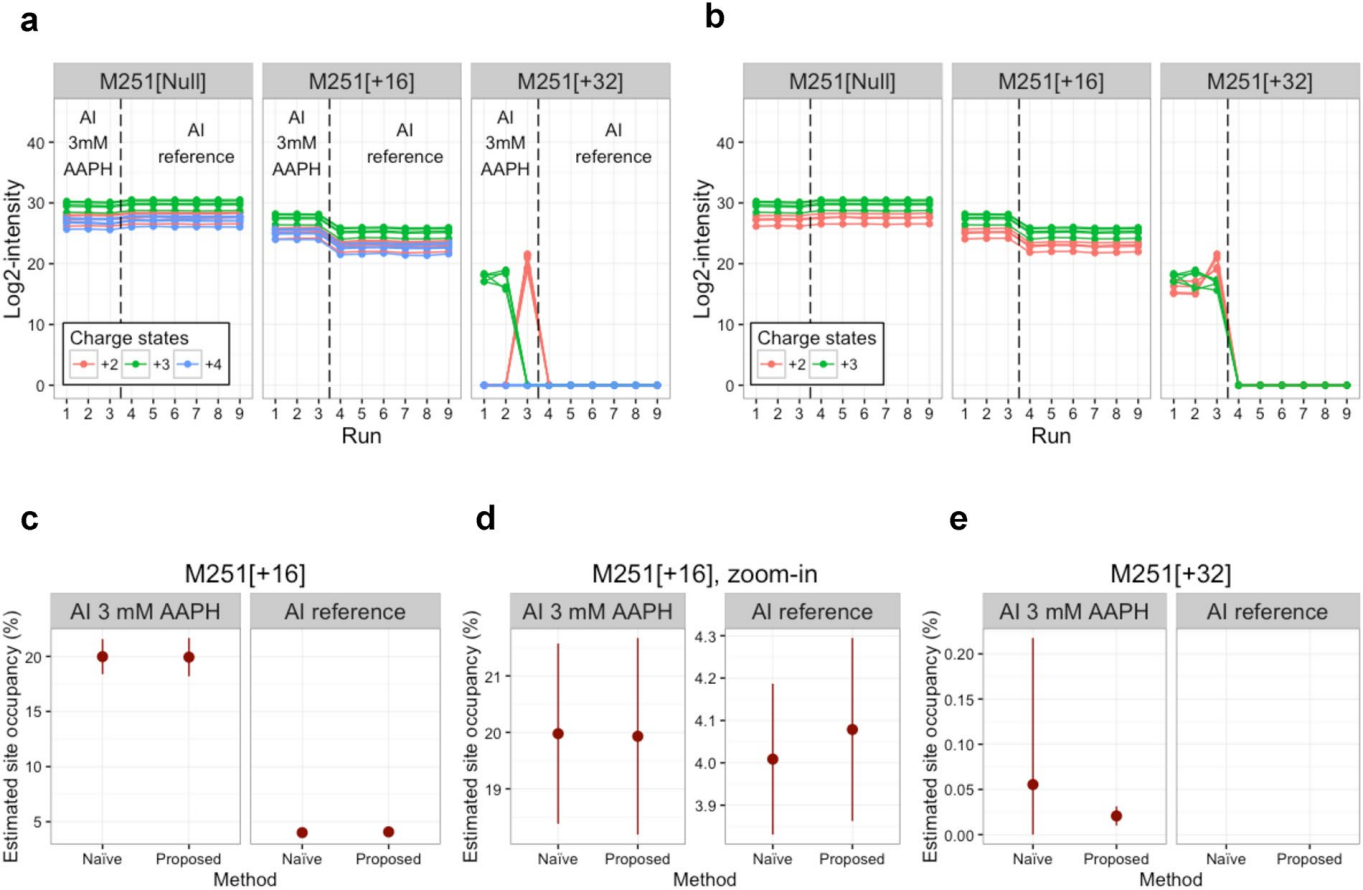
Goal 1: site occupancy estimation. Statistical characterization of site occupancy at site M251 in AI 3 mM AAPH and AI reference samples. (**a**) Profile plot of the features at site M251. There are three replicates in AI 3 mM AAPH (left) and six in AI reference (right). The features of the triply charged ions for the oxidative form M251[+32] are missing in the third replicate of the AI 3 mM AAPH sample while the features of the doubly charged ions are only observed in that replicate. (**b**) Profile plot of the features after feature selection and imputation of censored missing values by the proposed method. (**c**) Estimated site occupancies of the form M251[+16] with 95% confidence intervals by the naïve and proposed methods. (**d**) Expanded view of (**c**). (**e**) As in (**c**), but for the form M251[+32].

Figure 5 illustrates an example using the proposed approach for site occupancy estimation at site M251 in AI 3 mM AAPH and AI reference samples. The profile plot (Fig. 5a) of observed feature log-intensities is a useful tool for exploratory data analysis. It helps detect features with inconsistent quantitative profiles and reveals distinct coverage of each feature in forms, runs and conditions. For example, the features of triply charged ions for the oxidative form M251[+32] were missing in the third replicate run of the AI 3 mM AAPH sample while the features of doubly charged ions were only detected in that run. The inconsistency among replicates suggested a re-examination of those features, where one possible explanation is irreproducible chromatography leading to suppression of the triply charged ion by co-eluting species. Regardless of the reason for inconsistent charge state distribution across these replicates, appropriate summarization must be robust to outlying and inconsistent measurements. Figure 5b shows the profile plot after the proposed selection of the representative features (with charge states +2 and +3) and imputation of censored missing values. The procedure effectively reduced the variability of summarized intensities between runs. On the other hand, the naïve approach quantified site occupancy in every LC-MS run, and hindered the assessment of the variability in feature intensities. When all the features can be consistently detected and quantified in a form, e.g., M251[+16], the difference in site occupancy estimate between the two methods was negligible (Fig. 5c and d). When measurement issues arose as in M251[+32], however, the proposed approach had improved efficiency with a narrower confidence interval than the naïve approach (Fig. 5e). Additional details, as well as results of the proposed approach combined with LogOfSum summarization are shown in Supplementary Fig. S7. Results for the estimation of site occupancy for N-linked glycoforms, including the summarization, model-based inference and the comparison with the estimates from N-linked glycan data by 2-AB HILIC and CE/LIF are provided in Supplementary Section S8.

### Experimental data, Goal 2: differential site occupancy

**Figure 6:**
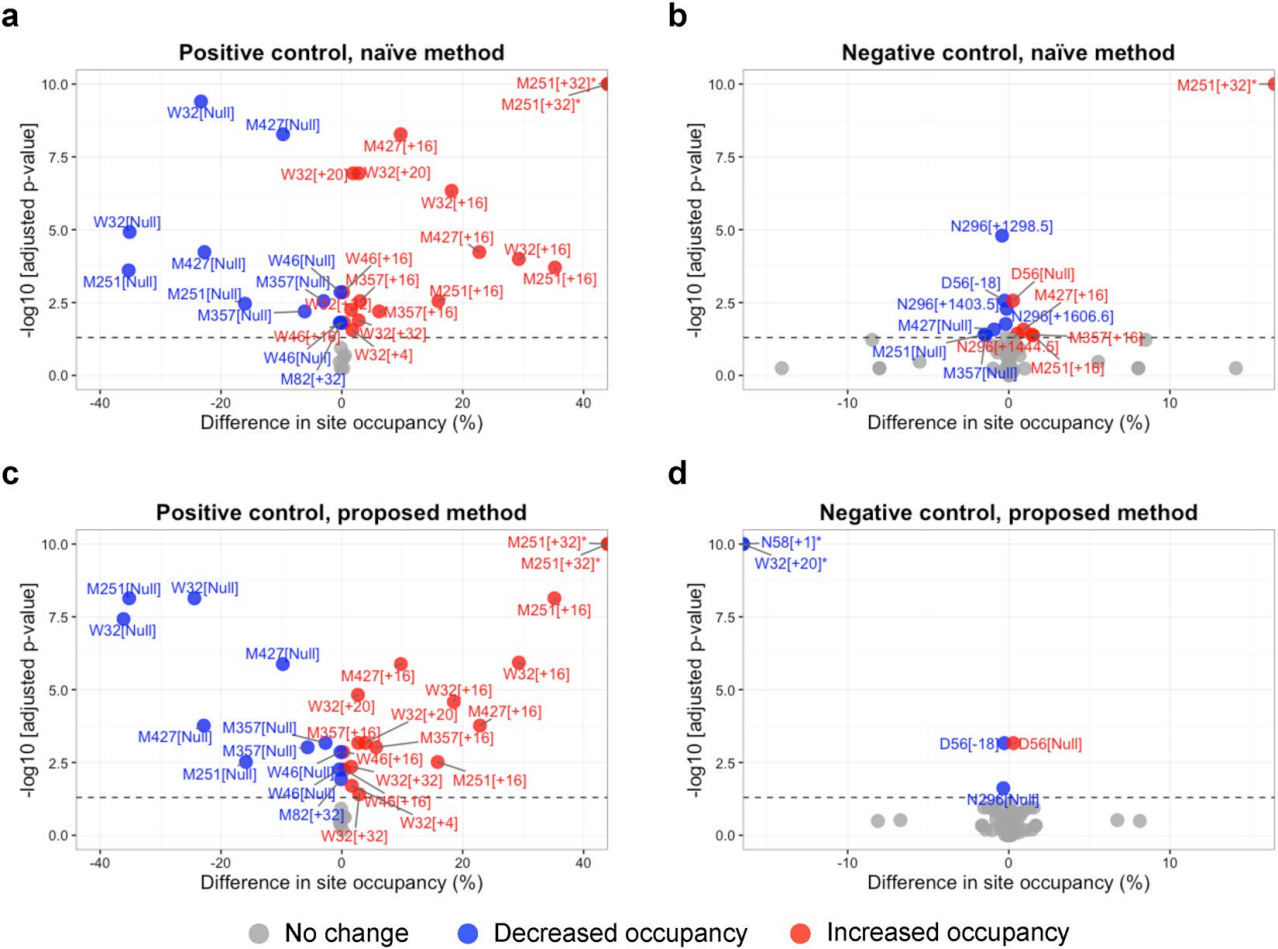
Goal 2: differential site occupancy. Volcano plots for the tests of differential site occupancy based on *H*_0_^(1)^. Each dot represents the testing result for differential site occupancy of one form. X-axis: practical significance, difference in site occupancy between conditions. Y-axis: statistical significance, FDR-adjusted *p*-values on the negative log10 scale. The horizontal dashed lines represent the 0.05 FDR cutoff. Forms with decreased and increased site occupancies are shown in blue and red colors, respectively. (**a**) Comparison of sites in Fab and Fc between AI 3 mM AAPH or AI 10 mM AAPH versus AI reference by the naïve method. All forms are expected to change in the comparison (positive control). (**b**) Comparison between ADC reference versus AI reference by the naïve method, excluding the sites for drug modifications. None of the forms is expected to change in the comparison (negative control). (**c**) As in (**a**), but by the proposed method. (**d**) As in (**b**), but by the proposed method.

The volcano plots (Fig. 6) summarize the results of differential site occupancy for the positive and negative controls, in terms of practical significance (i.e., difference in site occupancy) and statistical significance for each form. Statistical significance determines whether a difference in site occupancy between conditions is more systematic than as expected by random chance, based on *H*_0_^(1)^. Adjustment for multiple comparisons was performed using the Benjamini-Hochberg procedure to control the false discovery rate (FDR) at 0.05. The sensitivity, specificity and positive predictive value (PPV) by the naïve approach and proposed approach are summarized in Table 1. Compared to the naïve approach, the proposed approach provided the same sensitivity with improved specificity and PPV. Similar performance is observed for the proposed approach with either the TMP or the LogOfSum summarization (Supplementary Table S4). The results based on *H*_0_^(2)^ and *H*_0_^(3)^ are shown in Supplementary Section S9.2.

**Table 1:** Results of differential site occupancy analysis at the FDR cutoff 0.05 in comparisons for positive and negative controls.

Approach	Positive control		Negative control		Sensitivity	Specificity	PPV
	TP	FN	FP	TN			
Naïve	28	6	13	49	0.824	0.790	0.683
Proposed	28	6	5	56	0.824	0.918	0.848

The hypothesis testing is based on the null hypothesis *H*_0_^(1)^ as in Fig. 3c. The comparison between AI 3 mM AAPH or AI 10 mM AAPH versus AI reference samples for sites in Fab and Fc is considered as positive control, where all forms are expected to change. The comparison between ADC reference and AI reference samples excluding the sites for the linker-drug modifications is considered as negative control, where none of the forms is expected to change. Performances of the naïve approach and the proposed approach are compared based on sensitivity, specificity, and positive predictive value (PPV). Sensitivity is calculated as TP/(TP + FN), where TP is the number of true positives and FN is the number of false negatives. Specificity is calculated as TN/(FP + TN), where FP is the number of false positives and TN is the number of true negatives. PPV is calculated as TP/(TP + FP).

**Figure 7:**
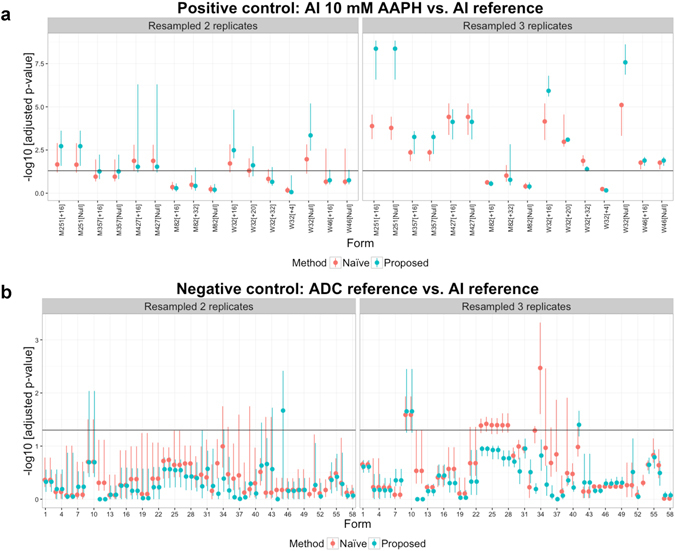
Differential analysis with small sample sizes. The replicate LC-MS/MS runs of the AI 10 mM AAPH, AI reference and ADC reference samples were resampled to a size of 2 or 3 per condition (without replacement) prior to the differential analysis based on *H*_0_^(1)^. X-axis: compared forms. Y-axis: statistical significance, FDR-adjusted *p*-values on the negative log10 scale. The horizontal lines indicate the 0.05 FDR cutoff. For each form, the vertical line represents the range between the maximum and minimum significance values among the testing results for the resampled data, and the dot represents the medium value. (**a**) The results for the positive controls (AI 10 mM AAPH versus AI reference). (**b**) The results for the negative controls (ADC reference versus AI reference). The list of compared forms for the negative controls is shown in Supplementary Table S6.

We evaluated the performance of the proposed method and naïve approach for differential analysis in scenarios of small sample sizes, with a focus on the samples used for the positive and negative controls. In the original dataset, there are three replicate LC-MS/MS runs for the AI 3 mM AAPH, AI 10 mM AAPH and ADC reference samples, and six replicate runs for the AI reference sample. These runs were resampled to a size of 2 or 3 per condition (without replacement) prior to the differential analysis (based on *H*_0_^(1)^). The results for the positive controls (AI 10 mM AAPH versus AI reference) and negative controls (ADC reference versus AI reference) are shown in Fig. 7, where the statistical significance is represented based on FDR-adjusted *p*-values on the negative log10 scale. The maximum, medium and minimum significance values among the testing results for the resampled data are shown for each form. With small sample sizes, the proposed method improved the overall performance in the differential analysis (higher values of statistical significance in the positive controls and lower ones in the negative controls) compared to the naïve approach. Performances based on average sensitivity, average specificity, and range of PPV over resampled replicates are summarized in Supplementary Table S7. More details and results are provided in Supplementary Section S9.3.

### Experimental data, Goal 3: combined occupancy estimation

Site occupancies and combined occupancies for oxidative modifications in Fab and Fc in the AI reference sample were estimated by the naïve approach and the proposed method (as defined in Fig. 3d). The estimation accuracy was evaluated by comparing the estimates with the LC/UV IdeS data. Overall, the considered methods with LC-MS/MS data provided similar estimates of combined occupancies, slightly lower than those from the LC-UV data (Supplementary Section S10).

### Evaluation of model assumptions

Appropriate statistical inference requires the choice of statistical model that characterizes and summarizes the data on a scale in good agreement with the model assumptions. While often left without indication, the implicit model for the naïve approach assumes that the residuals from the predicted site occupancy (average over runs) are independent and distributed as a normal distribution (Supplementary Section S3). On the other hand, the proposed method for model-based inference is based on the modeling on the scale of summarized feature intensities. Evaluation of model assumptions based on the residual plot and quantile-quantile (Q-Q) plot is shown in Supplementary Section S11.

## Discussion

Therapeutic protein characterization benefits from high sequence coverage and site-specific resolution of peptide mapping by LC-MS/MS. At the same time, it imposes high accuracy requirements to both estimation of site occupancy, and detection of differential site occupancy between conditions. In this manuscript, we proposed an effective statistical approach that aims for three goals: (1) *site occupancy estimation*, (2) *differential site occupancy*, and (3) *combined occupancy estimation*. The proposed approach characterized observed feature intensities with statistical models, translated each goal into model-based quantity, defined optimal inference procedure, and formalized associated challenges. In the following we discuss critical considerations and implications of the results: (1) use of replicates, (2) selection of consistent features for data processing and summarization, (3) hypothesis testing for differential site occupancy, (4) objective feature curation, and (5) limitation of LC-MS/MS-based peptide mapping for protein characterization.

Replicated LC-MS/MS runs play a key role throughout the proposed approach. Comparisons among replicates (as in Fig. 5a) allowed us to spot potential issues in sample preparation, and perform consistent data processing. Importantly, replicates were essential for characterizing the uncertainty associated to the quantity of interest. This includes the estimation of confidence intervals for site occupancy (Figs 4 and 5) and combined occupancy (Supplementary Fig. S18). These experiments were required for an objective determination of systematic changes between conditions (Fig. 6). In contrast, unreplicated studies have to rely on heuristics, by denoting a certain change in site occupancy as a significant change. As can be seen from the figures, reporting the percentage difference without replication undermines the accuracy and the reproducibility of the results. In contrast, increasing the number of replicates improves the accuracy of the results, even in the presence of missing values and interferences, and facilitates the automation of data analysis. Analyses such in this manuscript can be performed using data from pilot experiments, to select the appropriate number of LC-MS/MS runs.

Selection of consistent features for data processing and summarization across all the forms and conditions enables the comparison of their abundance estimates. This is a crucial aspect of the proposed approach. Since the definition of site occupancy involves all the possible forms at a same site (Fig. 3b), the proposed approach avoids confounding that can arise when different forms are characterized by features with different ionization efficiency. By selecting consistent features, the proposed approach also reduces the negative impact of missing values and outlying peaks. As the result, the proposed approach improved the consistency of summarization across forms and conditions, and facilitated the goals of protein characterization (Figs 5–7 and Table 1).

Statistical hypothesis testing provides an objective way to determine differential site occupancies. Among the three considered hypotheses, the testing based on *H*_0_^(1)^ that relates directly to a difference in site occupancy outperformed *H*_0_^(2)^ and *H*_0_^(3)^. However, it should be noted that the definitions of positive and negative controls in the evaluation relied on an understanding of the chemical processes of conjugation and stress. We did not collect additional orthogonal experimental measurements to confirm the defined positive and negative controls in this study. As a result, the positive and negative controls may not be exact, and the true extent of changes in site occupancies between conditions may be unknown. Moreover, hypotheses *H*_0_^(2)^ or *H*_0_^(3)^ differ in the use of a global reference peptide. When there is a substantial run-to-run variation in feature intensities, the difference can lead to remarkable distinction and the testing based on *H*_0_^(3)^ is expected to be more reliable. In this work, however, testing based on either *H*_0_^(2)^ or *H*_0_^(3)^ gave similar results (Supplementary Table S5).

Manual curation of abundances of LC-MS/MS features, reported by automated tools such as Skyline, is often necessary. The results indicate that exploratory data analysis, evaluation of model assumptions, and comparison with orthogonal data all facilitate the iterative process, and can help improve the workflow and produce more objective results. To ensure the consistency across forms, revisions (such as adjustments to boundaries for peak integration) should be applied to the entire dataset, (as opposed to a manually selected subset of runs). When comparison with data from orthogonal methods is used to evaluate the performance of the workflow, the signal processing and the statistical analysis of LC-MS/MS data should be done independently to ensure the objectivity of the orthogonal evaluation.

In the comparison of combined occupancy for total oxidation (Supplementary Section S10), the estimates by the considered approaches using LC-MS/MS data were all slightly lower than the results from the orthogonal LC/UV IdeS data. This may be due to mis-identification or undetected LC-MS features for the oxidative modifications, and better analytical methods and signal processing tools are needed. It should be also noted that the current multi-site approximation gives equal weight to each site when combining the abundance, assuming that peptide features at different sites share an identical ionization efficiency. This assumption may not always be valid as the ionization efficiency is expected to vary for different peptide sequences. More accurate estimate of the combined occupancy may be achieved by incorporating labeled reference peptides for the associated sites.

Overall, this manuscript offers a statistical approach, which enables an efficient use of LC-MS/MS-based peptide mapping for therapeutic protein characterization. The proposed method improved the accuracy of site occupancy estimation, and the detection of differential site occupancy between conditions. The improvements were particularly notable in situations with fewer LC-MS/MS replicate runs, and in presence of missing peaks. The proposed approach can be applied to datasets with other label-free chromatography-based quantification methods such as data-independent acquisition. Among the proposed analysis steps, the summarization of feature intensities for each modification form is the most computationally intensive. The TMP summarization employed in the proposed approach is the same as implemented in a more general software MSstats^21^, which has been applied to numerous large-scale LC-MS/MS datasets, and we expect similar scalability of the proposed approach. This general statistical modeling framework can be extended to other experimental designs. It can be used to enhance data processing strategies, and to evaluate the results using orthogonal experiment types. We advocate the use of the proposed approach in practice. The R code implementing the proposed approach, as well as the evaluation dataset (Dataset 2), are publicly available.

## Methods

### Sample preparation

Forced degradation and enriched minor variant samples (preparative ion exchange chromatography for the AI and preparative free flow electrophoresis for the ADC) were prepared in a manner similar to what previously described^22^. Since the formulation buffer and stress conditions of the AI and ADC were different, no direct comparisons were made between the AI forced degradation and enriched minor variant samples and those of the ADC. *In vitro* proteolysis of all samples was performed with endolysC, reduction was performed with TCEP, and carboxymethylation of cysteine residues was performed with iodoacetic acid.

### LC-MS/MS analysis

A Fusion trihybrid LC-MS/MS system with electron transfer dissociation (ETD) and high energy collision-induced dissociation (HCD) (Thermo, San Jose, CA) with a binary ultrahigh performance high pressure LC pump (H class, Waters), a 70 min reverse-phase 0.1% triflouroaceticacid/acetonitrile gradient at 0.25 mL/min, and a Zorbax C8 column (300-SB, 2.1 × 100 mm, 1.8 μm, Agilent, San Jose, CA) at 60 °C was employed to collect 3–6 technical replicates per sample, where only the last 3 replicates, collected in randomized fashion several months later than the initial technical replicates, are the focus of this work. Data-dependent acquisition was performed where the top 5 most abundant ions were selected with a unique MS/MS scan function with 120k mass resolution in the MS dimension.

### Peptide identification

Both Pepfinder (Thermo, San Jose, CA)^13^ and Mascot (Matrixscience)^23^ were employed for sequence database searching. Both Pepfinder and Mascot settings included: 2 missed cleavages, no static modifications, and the amino acid residue-specific variable modifications discussed in the main text. Several precautions were taken to minimize the omission of low abundance peptides due to inadvertent data reduction during automated or manual data analysis. First, we combined the accurate mass and time tag strategy^24^ of LC-MS-based peak assignments with Pepfinder and Skyline with the ‘shotgun’ proteomics strategy of LC-MS/MS-based peak assignments with Pepfinder and Mascot. Moreover, Xcalibur and Skyline assignments were based on manual inspection of LC-MS, LC-MS/MS and UV absorption data. Second, we employed relatively less stringent settings for each of these tools in combination than we might have if we had used any one tool alone (despite detailed knowledge of the most abundant modifications for each peptide). Pepfinder contained a single amino acid sequence for each of the heavy and light chains of the cysteine-conjugated ADC and its AI. The Mascot database contained more than 300 amino acid sequences for the light and heavy chains. Pepfinder used both LC-MS and LC-MS/MS for the identification, and Mascot used solely LC-MS/MS. MS-based identifications are particularly important for labile chemical modifications such as linker-drug modifications where high quality MS/MS spectra can be difficult to achieve and can challenge sequence database search algorithms. Pepfinder and Mascot were employed with the manufacturer’s recommended settings for the Fusion trihybrid LC-MS/MS system with ETD/HCD, including: 5–10 ppm mass tolerance for extracted ion chromatograms and no abundance threshold.

### Chromatography-based quantification

Based on the results of the sequence database search, Skyline was used to detect and quantify the chromatographic peaks in the LC-MS- and LC-MS/MS profiles. Skyline settings included mass resolution of 120,000 at *m*/*z* 200, charge states +1 to +6, isotopes 0 to 4 (1^st^ to 5^th^ isotope), number of amino acid residues 0 to 70, and either the MS filter or the MS/MS-based library were used to automatically select peaks in extracted ion chromatograms prior to manual verification. A report was exported for each sample, which defined a peptide feature as a combination of charge state, isotope and modification, and quantified each peptide feature by the area under the curve. Subtle changes in retention time across samples were sometimes observed due to imperfect chromatographic reproducibility. Therefore, manual verification of qualitative peak assignments with Pepfinder and Mascot was performed within Xcalibur (Thermo) and Skyline. This included both a verification of a match between the theoretical and observed isotopic envelopes and a verification of consistent selection of retention times for integration of peaks in extracted ion chromatograms. Problematic peaks were corrected or removed as appropriate. For example, correct integration of chromatographically resolved deamidated species oftentimes required removal of the incorrectly selected ^13^C (2^nd^) isotope of the corresponding unmodified peptide. Likewise, correct integration of chromatographically resolved isobaric species, such as peptides with tryptophan oxidation, oftentimes required selection of a relatively wide retention time window in order to capture all important peptide features. Finally, peptide features that were incorrectly selected, as revealed by low (e.g., <0.7) isotopic dot product scores (idotp, a measure of the match to the theoretical isotopic envelope), or MS/MS-based peak assignments more than 5 min from the MS/MS library were removed. Xcalibur settings included a 5–10 ppm mass tolerance for extracted ion chromatograms and no abundance threshold. The data were exported into a custom report within Skyline for downstream analysis with R. The report included numerous fields for each peptide feature, including the idotp, isotope- and charge-based peak area, retention time, sequence, modified peptide sequence, etc.

### Orthogonal method: LC/UV IdeS Assay of the AI

Quantification of the total oxidation (Fab and Fc) of the AI with the LC/UV IdeS Assay was performed in a manner similar to that previously reported^19^. These results were compared to the LC-MS/MS-derived combined occupancies for the most reactive oxidation sites at W32, W46 and M82 in Fab and M251, M357 and M427 in Fc.

### Orthogonal method: 2-AB HILIC and CE/LIF of the AI

Quantification of N-linked glycans of the AI was performed with 2-AB HILIC and CE/LIF in a manner similar to that previously reported^15, 16^. These results were compared to the LC-MS/MS-derived combined occupancies for the most abundant N-linked glycopeptides, including: A1G0, M5, A2G0, A2G0F, A2G1F, A2G2F, A2S1G2F.

### Statistical model, parameter estimation and summarization

The observed log-intensity of a feature is represented using a linear mixed model in consideration of the effects of form, condition, run, feature and interaction between run and feature. The model parameters are estimated using the split-plot approach as in MSstats^21^ where feature intensities per run are summarized in a sub-plot model and a whole-plot model is used to carry out the model-based inference of the underlying abundance for each form in a condition. Three steps are involved in a sequence for the sub-plot summarization: (1) feature selection, (2) imputation of censored missing values, and (3) summarization of feature intensities. The step of feature selection searches a subset of features that are the most representative in terms of their coverage in forms and runs. An accelerated failure time (AFT) model is used to impute censored missing data whose values are assumed to be below the detection limit^25–27^. Finally, Tukey’s median polish (TMP) method is applied for robust estimation of model parameters. The AFT and TMP methods are applied as in MSstats^21^. After the summarization of feature intensities, the model-based inference of the underlying abundance of a form is then carried out using the whole-plot model based on the experimental design. The summarization and model-based inference are applied for all the three goals. More details are provided in Supplementary Section S4.1.

### Proposed approach for goal 1: site occupancy estimation

Site occupancy is estimated by the abundance of all forms at the same site as defined in Fig. 3b. This involves non-linear transformation of the log-abundance estimates of the forms, and we resort to the delta method to approximate the standard error (SE) of site occupancy estimate. The confidence interval can be further derived with the point estimate and SE of site occupancy. More details are provided in Supplementary Section S4.

### Proposed approach for goal 2: differential site occupancy

Statistical characterization of differential site occupancy tests the null hypothesis of ‘no change’ against the alternative. As shown in Fig. 3c, there are three hypotheses used in the differential analysis for a form, differing in the use of the normalizing factor in the denominator. Hypothesis *H*_0_^(1)^ states that there is no difference in site occupancy between conditions, where the abundance of the form is normalized by the sum of the abundances over all forms. Hypothesis *H*_0_^(2)^ states that there is no difference in log-abundance between conditions for the form. Hypothesis *H*_0_^(3)^ states that there is no difference in log-abundance between conditions for the form, normalized by the reference peptide. Based on the considered hypothesis, the test statistic is defined as the estimate of difference between the two conditions, divided by the SE of the estimate. The test statistic is then compared against the *t* distribution to determine the statistical significance of the difference. More details are provided in Supplementary Section S5.

### Proposed approach for goal 3: combined occupancy estimation

Combined occupancy is estimated by the abundance of the form across multiple sites as defined in Fig. 3d. Similarly to the site occupancy estimation, a non-linear transformation of the log-abundance estimates is involved, and the delta method is used to approximate the SE of the combined occupancy estimate with forms across multiple sites involved. The confidence interval is given with the point estimate and SE of the combined occupancy. More details are provided in Supplementary Section S6.

An example R markdown file is provided in the Supplementary Information, which demonstrates the whole workflow including data importing and manipulation, exploratory data analysis, visualization and the proposed statistical methods for the three goals.

## Electronic supplementary material

Supplementary Materials

Dataset 1

Dataset 2
