# Supplementary material for: Statistical characterization of therapeutic protein modifications
Source: Sci Rep. 2017 Aug 11;7:7896. doi: 10.1038/s41598-017-08333-y (PMC5554216; doi:10.1038/s41598-017-08333-y)
Supplement: Supplementary file 1 — Supplementary Materials [file 41598_2017_8333_MOESM1_ESM.pdf]

Statistical characterization of therapeutic protein modifications

Supplementary Materials

Tsung-Heng Tsai[1], Zhiqi Hao[2], Qiuting Hong[2,5], Benjamin Moore[2], Cinzia Stella[2], Jeffrey H. Zhang[2], Yan Chen[2], Michael Kim[2], Theo Koulis[3], Gregory A. Ryslik[3], Erik Verschueren[4], Fred Jacobson[2], William E. Haskins[2], and Olga Vitek[1]

[1]Northeastern University, Boston, MA, USA
[2]Protein Analytical Chemistry, Genentech, South San Francisco, CA, USA
[3]Nonclinical Biostatistics, Genentech, South San Francisco, CA, USA
[4]Protein Chemistry, Genentech, South San Francisco, CA, USA
[5]Eurofins Lancaster Laboratories, Lancaster, PA, USA

June 11, 2017

Contents

1 The motivating example **3**

2 Overview of proposed approach and notation **9**

3 Currently used method and its implementation **10**

4 Proposed approach for Goal 1: site occupancy estimation **12**

4.1 Statistical model for input data, parameter estimation and summarization 12

4.2 Model-based inference for Goal 1 15

4.3 Alternative to Step 1 in Section 4.1 15

5 Proposed approach for Goal 2: differential site occupancy **16**

5.1 Statistical model for input data, parameter estimation and summarization 17

5.2 Model-based inference for Goal 2 17

6 Proposed approach for Goal 3: combined occupancy estimation **18**

6.1 Statistical model for input data, parameter estimation and summarization 18

6.2 Model-based inference for Goal 3 18

7 Computer simulation **20**

7.1 Computer simulation for Goal 1 20

7.2 Computer simulation for Goal 2 20

8 Details for the example: site occupancy estimation **24**

9 Details for the example: differential site occupancy **28**

9.1 Differential site occupancy based on $H_0^{(1)}$ 28

9.2 Differential site occupancy based on $H_0^{(2)}$ and $H_0^{(3)}$ 28

9.3 Differential site occupancy analysis with small sample sizes 31

10 Details for the example: combined occupancy estimation **34**

11 Evaluation of model assumptions **35**

1 The motivating example

Table S1: Monoisotopic mass shifts and molecular formulas of characteristic modifications in the motivating example, which include modifications observed at low (e.g., $< 1\%$) levels (or not at all) in reference samples, as well as modifications observed at high levels (e.g., $> 1\%$) in forced degradation samples (e.g., 3 mM AAPH).

Modification	Amino Acid Residue(s)	Monoisotopic Mass Shift (Da)	Molecular Formula
K/P cleavage	K/P	Sequence-dependent	Sequence-dependent
D/P	cleavage D/P	Sequence-dependent	Sequence-dependent
C-terminal lysine cleavage	/K	-128.094963	$-C_6H_{12}N_2O$
N-terminal Pyroglutamate	E	-18.010565	$-H_2O$
Carboxymethyl	C	+58.0054729	$+C_2H_2O_2$
Deamidation	NQ	+0.984016	$+H_2O$-NH_3 (+O-NH)
Hexose	K	+162.052824	$+C_6H_{10}O_5$
Isoaspartate	D	0 (isobaric)	–
Oxidation, Dioxidation	MW	+15.994915, +31.989829	+O, $+O_2$
Kynurenine	W	+3.994915	-C+O
Hydroxykynurenine	W	+19.989829	$-C+O_2$
Succinimide	N or D	-17.026549 or -18.010565	$-NH_3$ or $-H_2O$
vcMMAE	CK	+1315.779162	$+C_{68}H_{105}N_{11}O_{15}$
vcMMAEhydro	CK	+1333.789727	$+C_{68}H_{107}N_{11}O_{16}$
Non-glycosylated	N296	0	–
A2S1G2F (G2+NANA or 2121 or Hex5HexNAc4Fuc1NANA1)	N296	+2221.787757	$+C_{85}H_{139}N_5O_{62}$
A1G0 (G0-F-GlcNnac)	N296	+1095.396588	$+C_{42}H_{69}N_3O_{30}$
A1G0F (G0-GlcNac)	N296	+1241.454497	$+C_{48}H_{79}N_3O_{34}$
A1G1F (G1-GlcNac)	N296	+1403.507321	$+C_{54}H_{89}N_3O_{39}$
A2G0 (G0-F)	N296	+1298.475961	$+C_{50}H_{82}N_4O_{35}$
A2G0F (G0)	N296	+1444.533870	$+C_{56}H_{92}N_4O_{39}$
A2G1F (G1)	N296	+1606.586693	$+C_{62}H_{102}N_4O_{44}$
A2G1 (G1-F-GlcNac) or A2G0M4	N296	+1460.528784 (isobaric)	$+C_{56}H_{92}N_4O_{40}$
A2G2F (G2)	N296	+1768.639517	$+C_{68}H_{112}N_4O_{49}$
A2S1G0F	N296	+1897.68211	$+C_{73}H_{119}N_5O_{52}$
A3G1F	N296	+1809.666066	$+C_{70}H_{115}N_5O_{49}$
A3G0F (3100)	N296	+1647.613242	$+C_{64}H_{105}N_5O_{44}$
A3G0 (3000)	N296	+1501.555334	$+C_{58}H_{95}N_5O_{40}$
A4G0	N296	+1704.634706	$+C_{66}H_{108}N_6O_{45}$
A4G1	N296	+1866.68753	$+C_{72}H_{118}N_6O_{50}$
M5	N296	+1216.422863	$+C_{46}H_{76}N_2O_{35}$
M6	N296	+1378.475686	$+C_{52}H_{86}N_2O_{40}$
M7	N296	+1540.52851	$+C_{58}H_{96}N_2O_{45}$
M8	N296	+1702.58133	$+C_{64}H_{106}N_2O_{50}$

Table S2: Characteristic peptides and modified amino acid residues. Peptide sequence is shown with its [start position, stop position].

	Heavy Chain
$[0, 64]$	-.EVQLVESGGGLVQPGGSLRLSCAASGYTFSSYWIEWVRQAPGK∧GLEWIGEILPGGGDTNYNEIFK.G
	∧ = unexpected missed cleavage site
$[0, 42]$	.EVQLVESGGGLVQPGGSLRLSCAASGYTFSSYWIEWVRQAPGK.G
$[43, 75]$	K.GLEWIGEILPGGGDTNYNEIFK*GRATFSADTSK.N
	* = missed cleavage due to K modification
$[43, 64]$	K.GLEWIGEILPGGGDTNYNEIFK.G
$[76, 120]$	K.NTAYLQMNSLRAEDTAVYYCTRRVPIRLDYWGQGTLVTVSSASTK.G
$[218, 221]$	K.SCDK.T
$[222, 247]$	K.THTCPPCPAPELLGGPSVFLFPPK∧PK.D
	∧ = missed cleavage due to K/P cleavage site
$[222, 245]$	K.THTCPPCPAPELLGGPSVFLFPPK.∧P
	∧ = K/P cleavage site
$[248, 273]$	K.DTLMISRTPEVTCVVVDVSHEDPEVK.F
$[288, 316]$	K.TK∧PREEQYNSTYRVVSVLTVLHQDWLNGK.E
	∧ = missed cleavage due to K/P cleavage site
$[290, 316]$	K.∧PREEQYNSTYRVVSVLTVLHQDWLNGK.E
	∧ = K/P cleavage site
$[340, 359]$	K.GQPREPQVYTLPPSREEMTK.N
$[414, 438]$	K.SRWQQGNVFSCSVMHEALHNHYTQK.S
$[439, 446]$	K.SLSLSPG∧K.-
	∧ = C-terminal K cleavage site
$[153, 172]$	K.VDNALQSGNSQESVTEQDSK.D
	Reference peptide
	Light Chain
$[0, 45]$	-.DIQLTQSPSSLSASVGDRVTITCK*ASQSVDYEGDSFLNWYQQK∧PGK.A
	* = missed cleavage due to K modification
	∧ = missed cleavage due to K/P cleavage site
$[24, 45]$	K.ASQSVDYEGDSFLNWYQQK*∧PGK.A
	* = missed cleavage due to K modification
	∧ = missed cleavage due to K/P cleavage site
$[24, 42]$	K. ASQSVDYEGDSFLNWYQQK. P
$[49, 106]$	K.LLIYAASNLESGVPSRFSGSGSGTDFTLTISSLQPEDFATYYCQQSNED∧PLTFGQGTK.V
	∧ = D/P cleavage site
$[49, 97]$	K.LLIYAASNLESGVPSRFSGSGSGTDFTLTISSLQPEDFATYYCQQSNED∧
	∧ = D/P cleavage site
$[98, 106]$	D.∧PLTFGQGTK.V
	∧ = D/P cleavage site
$[211, 217]$	K.SFNRGEC.-

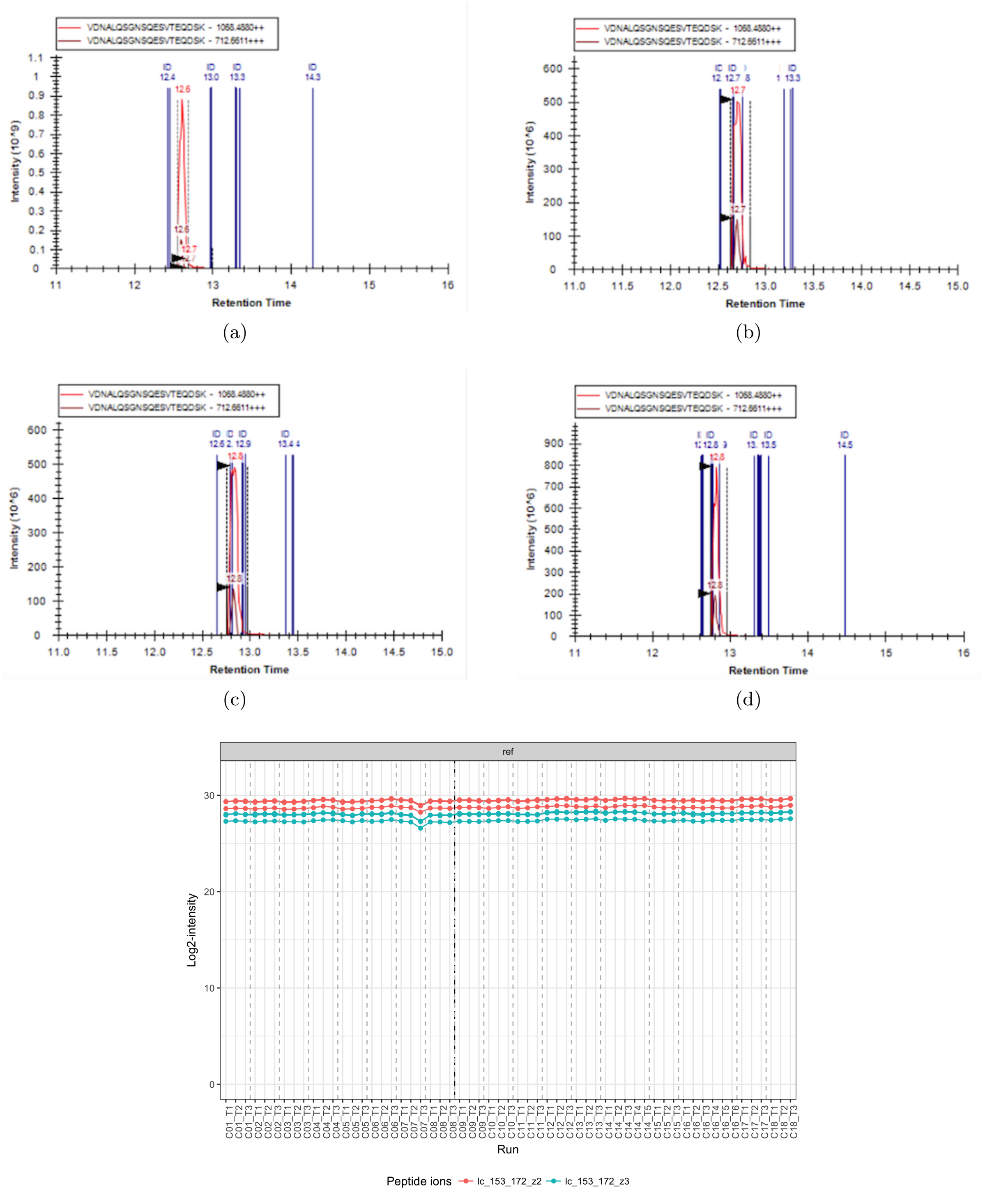

(e)

Figure S1: Representative extracted ion chromatogram of the normalizing peptide VDNALQSGNSQESVTEQDSK in samples of (a) AI reference, (b) ADC reference, (c) AI 3 mM AAPH, and (d) ADC 3 mM AAPH. (e) Profile plot of the area under the chromatographic curve of the reference peptide in all analyzed LC-MS/MS runs, where replicate runs of the same condition are arranged together and different conditions are separated by gray dashed lines. The black dashed line separates the 8 ADC samples (left) and the 10 AI samples (right).

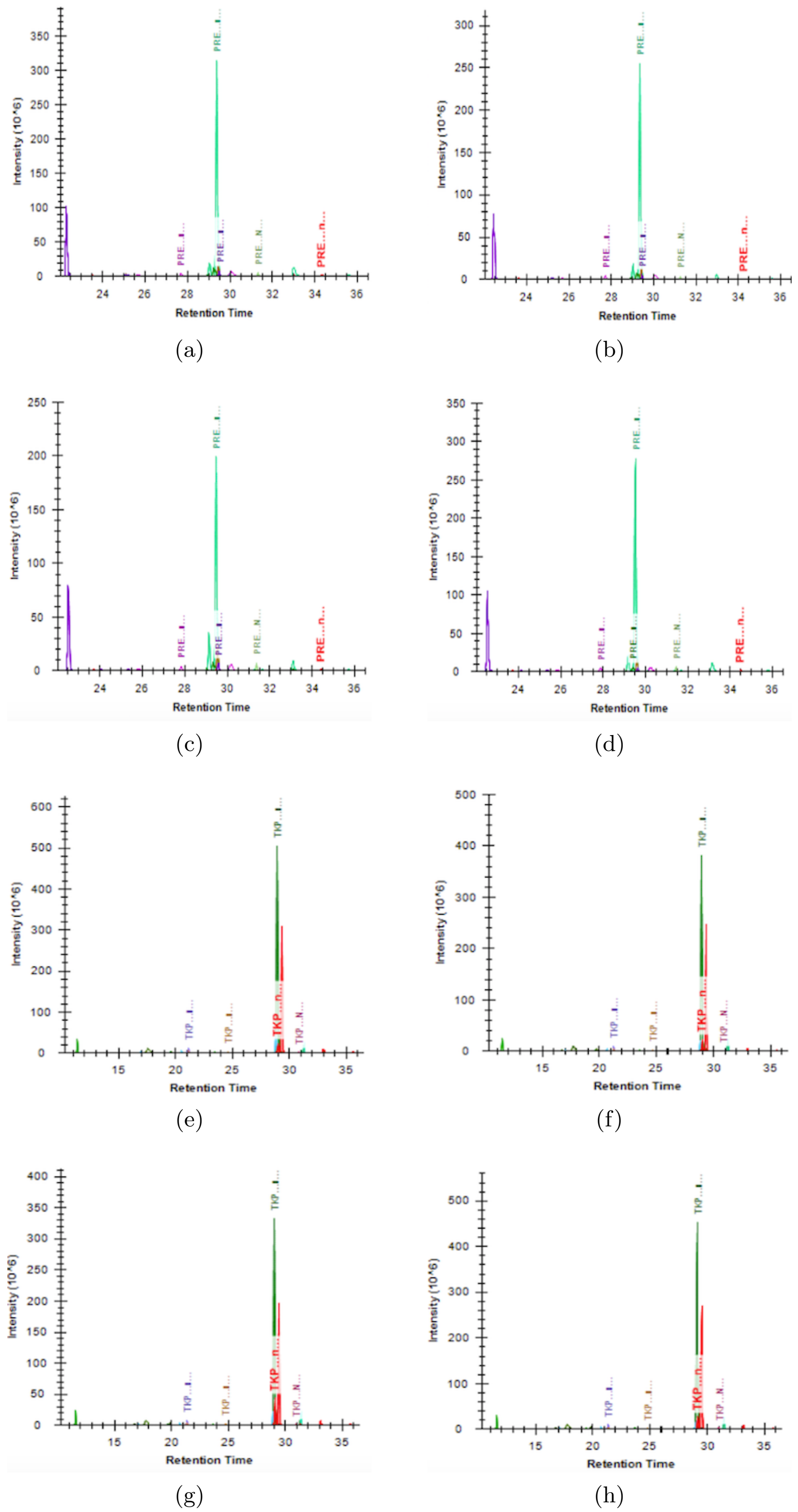

Figure S2: Representative extracted ion chromatograms for fully-cleaved N-linked glycopeptide in (a) AI reference, (b) ADC reference, (c) AI 3 mM AAPH, and (d) ADC 3 mM AAPH, and partially-cleaved N-linked glycopeptide in (e) AI reference, (f) ADC reference, (g) AI 3 mM AAPH, and (h) ADC 3 mM AAPH. Both the fully- and partially-cleaved N-linked glycopeptides are observed at the same levels in AI and ADC samples.

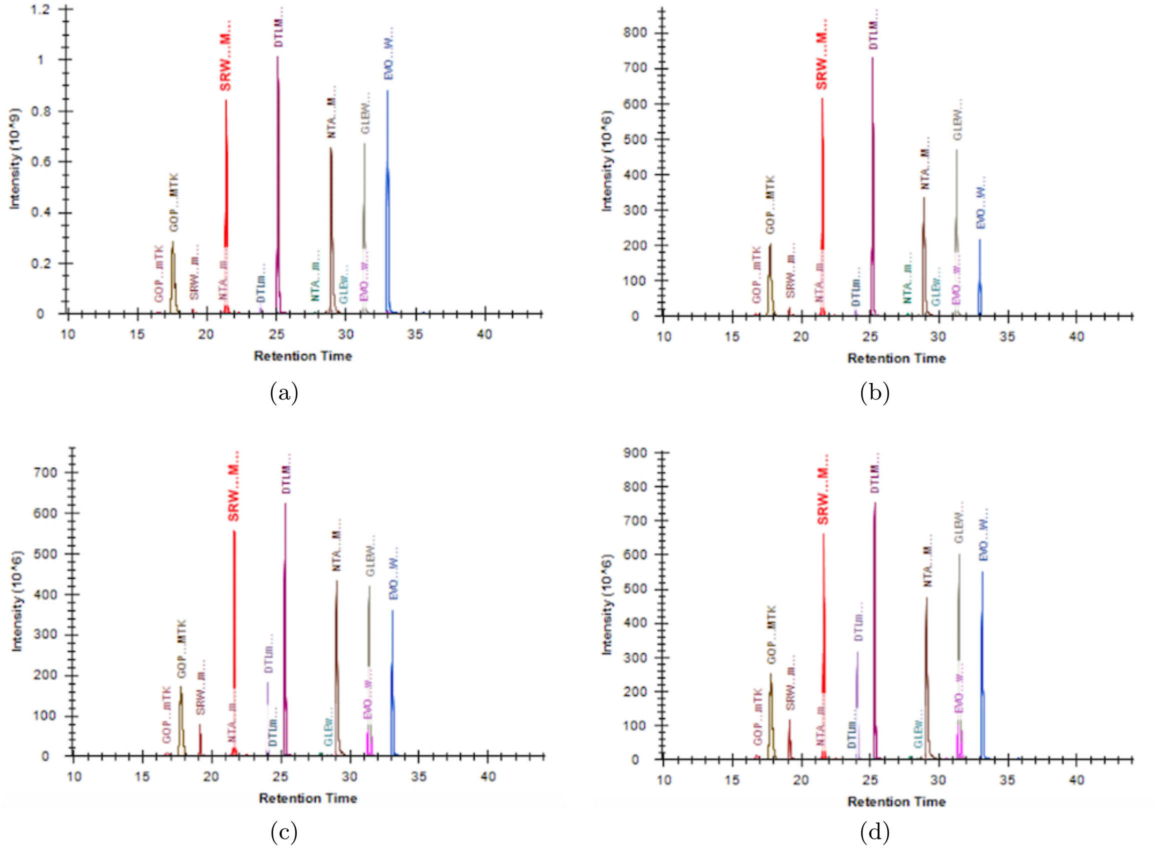

Figure S3: Representative extracted ion chromatograms for oxidized peptides in (a) AI reference, (b) ADC reference, (c) AI 3 mM AAPH, and (d) ADC 3 mM AAPH. The oxidized methionine in peptide 248-273 (K.DTL**M**ISRTPEVTCVVVDVSHEDPEVK.F) at 24 min is observed at higher levels in panels (c) and (d) compared to panels (a) and (b) for the 3 mM AAPH-treated AI and ADC samples, respectively.

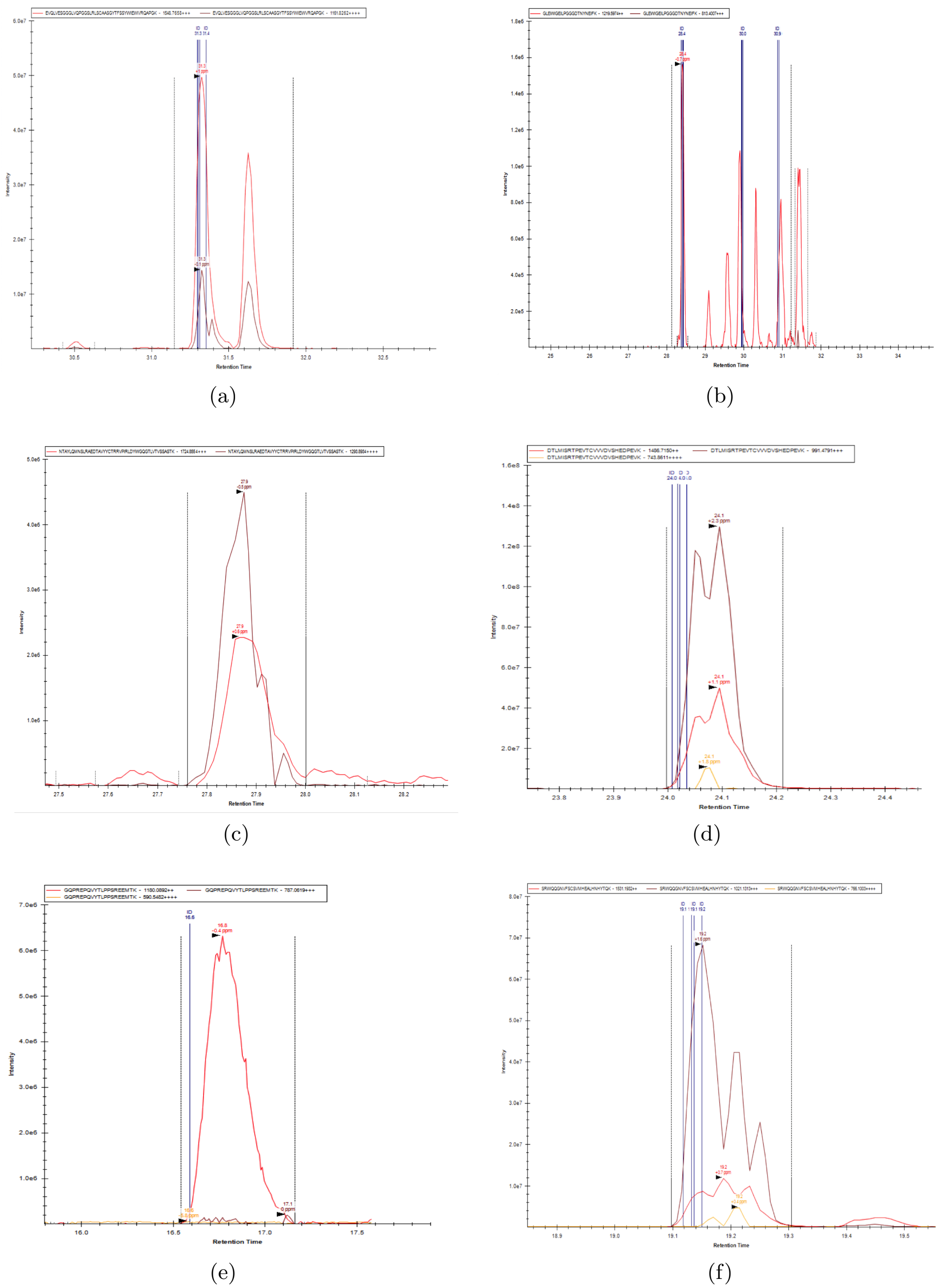

Figure S4: Expanded views of the extracted ion chromatograms for peptides with oxidative modifications in AI 3mM AAPH sample at sites: (a) W32, (b) W46, (c) M82, (d) M251, (e) M357 and (f) M427.

2 Overview of proposed approach and notation

We consider the following three goals for therapeutic protein characterization.

Goal 1: the objective estimation of site occupancy in a particular condition.

Goal 2: the determination of differential site occupancy between conditions.

Goal 3: the objective estimation of combined occupancy over multiple forms/sites in a condition.

Data structure of protein characterization experiments. A set of fully-cleaved and/or partially-cleaved peptides such as Table S2 with a same modification ranging over I sites are considered together, where the modification is represented by J forms. There are K conditions in the experiment, and L mass spectrometry runs (technical replicates) per condition. In total, there are $K \times L$ runs in the experiment. Each form is represented by multiple spectral features (isotopic peaks of peptide ions, distinguished by their mass shifts and charge states), where the number of features for Form j at Site i is M_{ij}. There is also a reference form at a reference site, that is not modified and its abundance is expected to stay identical across conditions. The log-intensity (base 2) of Feature m of Form j at Site i, in Run l of Condition k is denoted by y_{ijklm}.

			Condition 1				...	Condition K			
			Run 1	Run 2	...	Run L	...	Run 1	Run 2	...	Run L
Site 1	Form 1	Feature 1	y_{11111}	$-$	...	y_{111L1}	...	y_{11K11}	y_{11K21}	...	y_{11KL1}
		Feature 2	y_{11112}	y_{11122}	...	y_{111L2}	...	y_{11K12}	y_{11K22}	...	y_{11KL2}
		...	...	...	...	...	...	...	...	...	...
		Feature M_{11}	$y_{1111M_{11}}$	$y_{1112M_{11}}$	...	$y_{111LM_{11}}$	...	$y_{11K1M_{11}}$	$y_{11K2M_{11}}$	...	$y_{11KLM_{11}}$
	Form 2	Feature 1	y_{12111}	y_{12121}	...	y_{121L1}	...	y_{12K11}	y_{12K21}	...	y_{12KL1}
		Feature 2	y_{12112}	y_{12122}	...	$-$	...	y_{12K12}	y_{12K22}	...	y_{12KL2}
		...	...	...	...	...	...	...	...	...	...
		Feature M_{12}	$y_{1211M_{12}}$	$y_{1212M_{12}}$	...	$y_{121LM_{12}}$	...	$y_{12K1M_{12}}$	$y_{12K2M_{12}}$	...	$y_{12KLM_{12}}$
Site 2	Form 1	Feature 1	y_{21111}	y_{21121}	...	y_{211L1}	...	y_{21K11}	y_{21K21}	...	y_{21KL1}
		Feature 2	$-$	$-$	...	$-$	...	$-$	$-$	...	$-$
		...	...	...	...	...	...	...	...	...	...
		Feature M_{21}	$y_{2111M_{21}}$	$y_{2112M_{21}}$	...	$y_{211LM_{21}}$	...	$y_{21K1M_{21}}$	$y_{21K2M_{21}}$	...	$y_{21KLM_{21}}$
	Form 2	Feature 1	y_{22111}	y_{22121}	...	y_{221L1}	...	y_{22K11}	y_{22K21}	...	y_{22KL1}
		Feature 2	y_{22112}	$-$	...	y_{221L2}	...	y_{22K12}	y_{22K22}	...	y_{22KL2}
		...	...	...	...	...	...	...	...	...	...
		Feature M_{22}	$y_{2211M_{22}}$	$y_{2212M_{22}}$	...	$y_{221LM_{22}}$	...	$y_{22K1M_{22}}$	$y_{22K2M_{22}}$	...	$y_{22KLM_{22}}$
Ref. Site	Ref. Form	Feature 1	$y_{\mathrm{R}111}$	$y_{\mathrm{R}121}$	...	$y_{\mathrm{R}1L1}$	...	$y_{\mathrm{R}K11}$	$y_{\mathrm{R}K21}$	...	$y_{\mathrm{R}KL1}$
		Feature 2	$y_{\mathrm{R}112}$	$y_{\mathrm{R}122}$	...	$y_{\mathrm{R}1L2}$	...	$y_{\mathrm{R}K12}$	$-$	...	$y_{\mathrm{R}KL2}$
		...	...	...	...	...	...	...	...	...	...
		Feature M_{R}	$y_{\mathrm{R}11M_{\mathrm{R}}}$	$y_{\mathrm{R}12M_{\mathrm{R}}}$	...	$y_{\mathrm{R}1LM_{\mathrm{R}}}$	...	$y_{\mathrm{R}K1M_{\mathrm{R}}}$	$y_{\mathrm{R}K2M_{\mathrm{R}}}$	...	$y_{\mathrm{R}KLM_{\mathrm{R}}}$

Figure S5: Representation of the data, in a simplified case of two sites, two forms, K conditions and L replicate runs. A form in each site is quantified by multiple spectral features. Some spectral features can be missing (shown as $-$), either randomly in individual runs or completely from a particular form. In real practice, the number of runs can vary across conditions.

Figure S5 shows an example data representation in a simplified case, where the modification arises at two sites with two forms (unmodified and modified) for each. The modification forms are represented by different numbers of features. To address the goals, statistical analysis will have to summarize values in this table using appropriate statistical models, translate each goal into a model-based quantity of interest, and draw inference (i.e., characterize the uncertainty) about the quantity.

3 Currently used method and its implementation

The method used in current practice (termed *naïve method*) summarizes site occupancies (or combined occupancies) for each run separately as input for the three goals. In each run, a form is first quantified by summing the feature intensities over all charge states and isotopes. The site occupancy is then calculated as the proportion of the summarized value to the sum over all forms at the same site. Specifically, the site occupancy of Form j at Site i in Run l of Condition k is given by

$$p_{ijkl} = \frac{x_{ijkl+}}{\sum_{j'=1}^{J} x_{ij'kl+}}, \tag{1}$$

where x_{ijklm} is the feature intensity at the original scale (i.e., $x_{ijklm} = 2^{y_{ijklm}}$, where y_{ijklm} is as in Figure S5) and $x_{ijkl+} = \sum_{m=1}^{M_{ij}} x_{ijklm}$. Alternatively, with fewer features as input a form may be quantified by the maximum feature intensity or the maximum intensity of a charge state with all isotopes integrated.

Goal 1: site occupancy estimation. In a particular condition, the naïve method estimates the site occupancy as the average of the site occupancies calculated in individual runs of the condition (as in Eq. (1)), where the estimate of uncertainty associated with the summary is typically not reported. To reconstruct the meaning of the method, an underlying model for the procedure can be formulated as

$$p_{ijkl} = \pi_{ijk} + \epsilon_{\pi,ijkl}, \tag{2}$$

where π_{ijk} is the expected value of site occupancy of Form j at Site i in Condition k on average over runs and $\epsilon_{\pi,ijkl}$ is a random error term with zero mean, i.e., $\mathrm{E}[\epsilon_{\pi,ijkl}] = 0$, $\pi_{ijk} = \mathrm{E}[p_{ijk\cdot}]$. The estimate of π_{ijk} is

$$\hat{\pi}_{ijk} = \frac{\sum_{l=1}^{L} p_{ijkl}}{L} = \frac{p_{ijk+}}{L}, \tag{3}$$

where L is the number of runs. The $100 \times (1 - \alpha/2)$ percent confidence interval of the estimate is given by

$$\hat{\pi}_{ijk} \pm t_{L-1,\alpha/2}\mathrm{SE}(\hat{\pi}_{ijk}), \tag{4}$$

where $t_{L-1,\alpha/2}$ is the $100 \times (1 - \alpha/2)$ percentile of the t distribution with $L - 1$ degrees of freedom, and the standard error (SE) is estimated via the sample variance. This generates the confidence intervals for the naïve method shown in the main text (Figure 5).

Goal 2: differential site occupancy. Differential site occupancy is typically obtained, e.g., using a t-test, which takes as input the calculated site occupancy of a form in a run (i.e., p_{ijkl}). The approach tests the null hypothesis stating that there is no difference in mean site occupancy between Conditions k and k' for Form j at Site i against the alternative:

$$H_0^{(p)} : \pi_{ijk} - \pi_{ijk'} = 0$$
$$H_a^{(p)} : \pi_{ijk} - \pi_{ijk'} \neq 0$$

While often left without indication, the implicitly assumed model for the testing is

$$p_{ijkl} = \pi_{ijk} + \epsilon_{\pi,ijkl}, \tag{5}$$

where $\epsilon_{\pi,ijkl} \overset{\text{iid}}{\sim} \mathcal{N}(0, \sigma^2_{\pi,ijk})$. The test statistic for the t-test is given by

$$\frac{\hat{\pi}_{ijk} - \hat{\pi}_{ijk'}}{\text{SE}\left(\hat{\pi}_{ijk} - \hat{\pi}_{ijk'}\right)} = \frac{\frac{1}{L}p_{ijk+} - \frac{1}{L}p_{ijk'+}}{\left[\text{SE}(\hat{\pi}_{ijk})^2 + \text{SE}(\hat{\pi}_{ijk'})^2\right]^{1/2}} = \frac{\frac{1}{L}p_{ijk+} - \frac{1}{L}p_{ijk'+}}{\left(\frac{1}{L}\hat{\sigma}^2_{\pi,ijk} + \frac{1}{L}\hat{\sigma}^2_{\pi,ijk'}\right)^{1/2}},$$

where $\hat{\sigma}^2_{\pi,ijk}$ and $\hat{\sigma}^2_{\pi,ijk'}$ are estimated as sample variances. The statistical significance is determined by comparing the test statistic against the t distribution with the effective degrees of freedom calculated using the Welch-Satterthwaite equation:

$$\frac{\left(\frac{1}{L}\hat{\sigma}^2_{\pi,ijk} + \frac{1}{L}\hat{\sigma}^2_{\pi,ijk'}\right)^2}{\frac{1}{L-1}\left(\frac{1}{L}\hat{\sigma}^2_{\pi,ijk}\right)^2 + \frac{1}{L-1}\left(\frac{1}{L}\hat{\sigma}^2_{\pi,ijk'}\right)^2}.$$

Goal 3: combined occupancy estimation. Similarly as in site occupancy estimation, the combined occupancy across sites is calculated in each run, followed by averaging the run-level summaries over all runs in the same condition. The combined occupancy of Form j in Run l of Condition k is denoted by $p^{(c)}_{jkl}$ and calculated as

$$p^{(c)}_{jkl} = \frac{x_{+jkl+}}{x_{++kl+}}, \tag{6}$$

where a form is quantified by summing the feature intensities over charge states, isotopes, and sites, i.e., $x_{+jkl+} = \sum_{i=1}^{I}\sum_{m=1}^{M_{ij}} x_{ijklm}$. The underlying model can be formulated as

$$p^{(c)}_{jkl} = \pi^{(c)}_{jk} + \epsilon^{(c)}_{\pi,jkl}, \tag{7}$$

where $\pi^{(c)}_{jk}$ is the expected value of combined occupancy of Form j in Condition k, and $\epsilon^{(c)}_{\pi,jkl}$ is a random error term with zero mean. The estimate of $\pi^{(c)}_{jk}$ is given by

$$\hat{\pi}^{(c)}_{jk} = \frac{1}{L}\sum_{l=1}^{L} p^{(c)}_{jkl} = \frac{1}{L}\sum_{l=1}^{L}\frac{x_{+jkl+}}{x_{++kl+}}, \tag{8}$$

and the $100 \times (1 - \alpha/2)$ percent confidence interval of the estimate is

$$\hat{\pi}^{(c)}_{jk} \pm t_{L-1,\alpha/2}\text{SE}(\hat{\pi}^{(c)}_{jk}). \tag{9}$$

4 Proposed approach for Goal 1: site occupancy estimation

The proposed data analysis approach for protein characterization is overviewed in Figure S6. Statistical characterization of site occupancy estimates the quantities underlying a peptide feature in each condition, replicate, site and form as illustrated in Figure 3b in the main text. This section describes the proposed method for estimation and inference of site occupancy at one site, including the inference procedure for the underlying abundance of a form and its site occupancy.

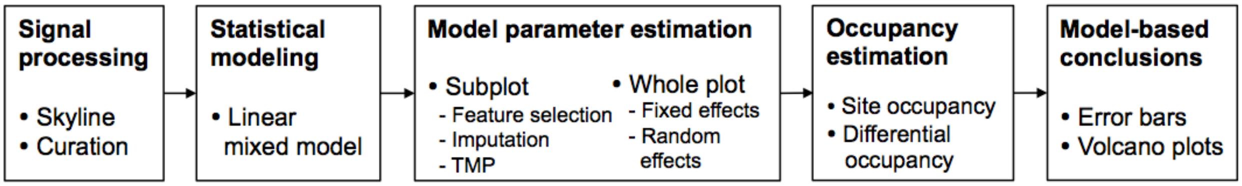

Figure S6: Overview of the proposed data analysis approach.

4.1 Statistical model for input data, parameter estimation and summarization

Model. In the proposed approach for estimating site occupancy of a form in a condition, the LC-MS features are viewed as repeated measurements of the underlying abundance of the form. Specifically, the observed log-intensity of Feature m of Form j at Site i, in Run l of Condition k (denoted by y_{ijklm}) is represented using a linear mixed model (4)

$$y_{ijklm} = \psi_{ij} + C_{ijk} + R_{ijl(k)} + F_{ijm} + (R \times F)_{ijklm}, \tag{10}$$

where ψ_{ij} is the expected value of log-abundance for Form j at Site i, and the effects of condition C_{ijk}, run $R_{ijl(k)}$, and feature F_{ijm} characterize how the intensity of each feature deviates from this reference. The interaction between run and feature $(R \times F)_{ijklm}$ is essentially viewed as a random noise. The effects of condition and feature are modeled as fixed effects:

$$\sum_{k=1}^{K} C_{ijk} = 0, \; \sum_{m=1}^{M_{ij}} F_{ijm} = 0, \tag{11}$$

and the effects of run and its interaction with feature are considered as random effects arising from normal distributions with mean 0:

$$R_{ijl(k)} \overset{\text{iid}}{\sim} \mathcal{N}(0, \sigma^2_{R,ijk}), \; (R \times F)_{ijklm} = \epsilon_{ijklm} \overset{\text{iid}}{\sim} \mathcal{N}(0, \sigma^2_{\epsilon,ij}). \tag{12}$$

The formulation for the observed log-intensity is similar to linear models in common use such as those in `MSstats` (1) except that the basic unit for quantification is a modification form rather than a protein.

Model parameter estimation. The model parameters in Eq. (10) are estimated using the split-plot approach as in `MSstats` (1). This approach reflects that features of the same run are acquired together as a block, and the parameter estimation is achieved by using a sub-plot model to summarize feature intensities per run and a whole-plot model to carry out the model-based inference of the underlying abundance.

Model parameter estimation, Step 1: run-level summarization. The sub-plot model used for the summarization of feature intensities for each form is given by

$$y_{ijklm} = \psi_{ij} + R_{ijl(k)} + F_{ijm} + \epsilon_{ijklm}, \quad (13)$$

where $\sum_{k=1}^{K}\sum_{l=1}^{L} R_{ijl(k)} = 0$, $\sum_{m=1}^{M_{ij}} F_{ijm} = 0$ and $\epsilon_{ijklm} \overset{\text{iid}}{\sim} \mathcal{N}(0, \sigma^2_{\epsilon,ij})$. Site occupancy of a form is determined by the abundance of all forms at the same site. Therefore, it is important to carry out the summarization for each form in a consistent fashion. Also, to reduce the impact of missing values on the estimation and summarization, appropriate imputation of missing values is needed. We describe below the three steps involved in a sequence for the sub-plot summarization: (a) feature selection, (b) imputation of censored missing value, and (c) summarization of feature intensities.

(a) Feature selection. To avoid the confounding due to different ionization efficiencies of features, the most representative features in terms of their coverage in forms and runs are selected for the summarization across runs. This step is translated into a weighted set cover problem (2). At Site i, there are $J \times K \times L$ pairs of forms and runs, denoted by a set of paired elements $E = \{\xi_{111}, \ldots, \xi_{JKL}\}$, where ξ_{jkl} represents the pair of Form j, Condition k and Run l. The number of unique features at the site is denoted by M. A feature may be missing in some forms and runs, and S_m denotes a subset of E, where Feature m is detected. Given the set $E = \{\xi_{111}, \ldots, \xi_{JKL}\}$, and a set of M subsets of E, $\mathcal{S} = \{S_1, S_2, \ldots, S_M\}$, the weighted set cover problem attempts to find a *least cost* collection $\mathcal{F}$ of sets from $\mathcal{S}$ such that the selected sets cover all the elements in E (2). That is, $\mathcal{F} \subset \{1, \ldots, M\}$ and $\cup_{m\in\mathcal{F}} S_m = E$. The cost function for each feature is defined as the number of (form × run) pairs where the feature is missing, i.e., $c(S_m) = |E \setminus S_m|$. In every iteration, inclusion of a feature is determined by the ratio of its cost to the number of newly added elements. The procedure to solve this problem is summarized in Algorithm 1. The algorithm assumes that every element of E must be covered by at least one of the M features. Uncovered elements need to be excluded before applying the algorithm.

Algorithm 1 Feature selection as a weighted set cover problem $(E, \mathcal{S}, c)$

Initialize $U \leftarrow E$ and $\mathcal{F} \leftarrow \emptyset$
while $U \neq \emptyset$ **do**
  Let m be the feature index which minimizes $\frac{c(S_m)}{|S_m \cap U|}$
  $\mathcal{F} \leftarrow \mathcal{F} \cup m$, $U \leftarrow U \setminus S_m$ (all tied features are included)
end while
Return $\mathcal{F}$

(b) Missing value imputation. An accelerated failure time (AFT) model is used to impute censored missing data whose values are assumed to be below the detection limit as in `MSstats` (1). The use of the AFT model in proteomics has also been explored in other studies (3, 5, 7). The imputation based on the AFT model is applied for each form separately, taking into account the effects of feature and run as well as the censoring threshold. Missing values are imputed for a feature when there is at least one measurement of the feature. In a rare case where none of the selected features is detected throughout all the forms, between-form imputation is carried out thereafter. The difference in log-intensity among the majority of features

(reflecting their relative ionization efficiencies) is assumed to be consistent across forms, and the intensity value for a completely missing feature is imputed based on the difference in log-intensity between features in other forms. Algorithm 2 summarizes the imputation procedure for y_{ijklm}, where Feature m is completely missing in Form j.

Algorithm 2 Between-form imputation $(\mathcal{F}, y)$

```
Let m be the index of the feature completely missing in Form j
Initialize Y ← ∅
for all m' ∈ F \ m do
   Initialize D ← ∅
   for all j' ∈ {1, . . . , J} \ j do
      D ← D ∪ median_{k,l}(y_{ij'klm} − y_{ij'klm'})
   end for
   Y ← Y ∪ (y_{ijklm'} + median(D))
end for
Return y_{ijklm} ← median(Y)
```

(c) Summarization of feature intensities. Tukey's median polish (TMP) method (6) is applied for robust estimation of the model parameters in Eq. (13) as in `MSstats` (1). The constraints for the robust estimation are $\text{median}_{k,l}(R_{ijl(k)}) = 0$, $\text{median}_m(F_{ijm}) = 0$, $\text{median}_{k,l}(\epsilon_{ijklm}) = 0$, and $\text{median}_m(\epsilon_{ijklm}) = 0$. The log-intensity of the site in each run is then represented as

$$\hat{y}_{ijkl} = \hat{\psi}_{ij} + \hat{R}_{ijl(k)}. \tag{14}$$

Model parameter estimation, Step 2: model-based inference of the underlying abundance. The whole-plot model represents the summarized log-intensity from the sub-plot model in consideration of the experimental design

$$\hat{y}_{ijkl} = \psi_{ij} + C_{ijk} + \theta_{ijkl}, \tag{15}$$

where $\sum_{k=1}^{K} C_{ijk} = 0$, $\theta_{ijkl} \overset{\text{iid}}{\sim} \mathcal{N}(0, \sigma^2_{\theta,ijk})$. We denote by μ_{ijk} the expected value of abundance for Form j at Site i in Condition k, on average over the effects of runs, features and their interactions, i.e.,

$$\log_2(\mu_{ijk}) = \text{E}[\hat{y}_{ijk\cdot}] = \psi_{ij} + C_{ijk}. \tag{16}$$

The log-abundance can be estimated by the summation of the estimates of the reference level ψ_{ij} and effect of condition C_{ijk}:

$$\log_2(\hat{\mu}_{ijk}) = \hat{\psi}_{ij} + \hat{C}_{ijk} = \frac{\hat{y}_{ijk+}}{L}, \tag{17}$$

and the standard error associated to the estimate is

$$\text{SE}\left(\log_2(\hat{\mu}_{ijk})\right) = \sqrt{\frac{1}{L}\hat{\sigma}^2_{\theta,ijk}}. \tag{18}$$

4.2 Model-based inference for Goal 1

The site occupancy of Form j is determined by the abundance of all forms at the same site and defined as

$$\phi_{ijk} = \frac{\mu_{ijk}}{\sum_{j'=1}^{J} \mu_{ij'k}} = \frac{\mu_{ijk}}{\mu_{i+k}}, \tag{19}$$

where the sum in the denominator is over all forms at Site i. With the estimates of the abundance as in Eq. (17), the estimate of the site occupancy is given by

$$\hat{\phi}_{ijk} = \frac{\hat{\mu}_{ijk}}{\hat{\mu}_{i+k}}. \tag{20}$$

This is a non-linear transformation of the model-based summaries of log-abundance, and we resort to the delta method to approximate the SE of the site occupancy estimate as

$$\mathrm{SE}(\hat{\phi}_{ijk}) = \left[\sum_{j'=1}^{J} \left(\frac{\partial \phi_{ijk}}{\partial \log_2(\mu_{ij'k})} \right)^2 \cdot \mathrm{SE}\left(\log_2(\hat{\mu}_{ij'k})\right)^2 \right]^{1/2}_{(\hat{\mu}_{i1k},\ldots,\hat{\mu}_{iJk})}, \tag{21}$$

where

$$\frac{\partial \phi_{ijk}}{\partial \log_2(\mu_{ij'k})} = \begin{cases} \left(\frac{1}{\mu_{i+k}}\right)^2 \cdot \log 2 \cdot \mu_{ijk} \cdot (\mu_{i+k} - \mu_{ijk}) & \text{if } j' = j \\ -\left(\frac{1}{\mu_{i+k}}\right)^2 \cdot \log 2 \cdot \mu_{ijk} \cdot \mu_{ij'k} & \text{if } j' \neq j \end{cases}$$

With the SE, the $100 \times (1-\alpha)$ percent confidence interval of the estimate is given by

$$\hat{\phi}_{ijk} \pm t_{df,\alpha/2} \mathrm{SE}(\hat{\phi}_{ijk}), \tag{22}$$

where $t_{df,\alpha/2}$ is the $100 \times (1-\alpha/2)$ percentile of the t distribution with degrees of freedom calculated using the Welch-Satterthwaite equation:

$$df = \frac{\left[\sum_{j'=1}^{J} \left(\frac{\partial \phi_{ijk}}{\partial \log_2(\mu_{ij'k})} \right)^2 \cdot \mathrm{SE}\left(\log_2(\hat{\mu}_{ij'k})\right)^2 \right]^2_{(\hat{\mu}_{i1k},\ldots,\hat{\mu}_{iJk})}}{\sum_{j'=1}^{J} \left[\frac{1}{L-1} \left(\frac{\partial \phi_{ijk}}{\partial \log_2(\mu_{ij'k})} \right)^4 \cdot \mathrm{SE}\left(\log_2(\hat{\mu}_{ij'k})\right)^4 \right]_{(\hat{\mu}_{i1k},\ldots,\hat{\mu}_{iJk})}}.$$

This generates the confidence intervals shown in the main text (Figure 5).

4.3 Alternative to Step 1 in Section 4.1

The log of sum (LogOfSum) method can be used as an alternative to summarize the feature intensities in each run, followed by the same procedures for the whole-plot modeling as described in Section 4.1 and the model-based inference for site occupancy as described in Section 4.2. Feature selection is not performed with this approach. The summarization procedure consists of (a) taking the sum of the feature intensities in each run at the original scale, and (b) applying the log-transformation to the sum. This effectively gives higher weights to features with higher intensities, and sets the intensities of missing values to 0. Different features may be used for the summarization in different forms and runs. The summarized value by the LogOfSum method can therefore be confounded by the ionization efficiency of feature. The procedure is illustrated in Figure S7 using the example as in Figure 5 of the main text.

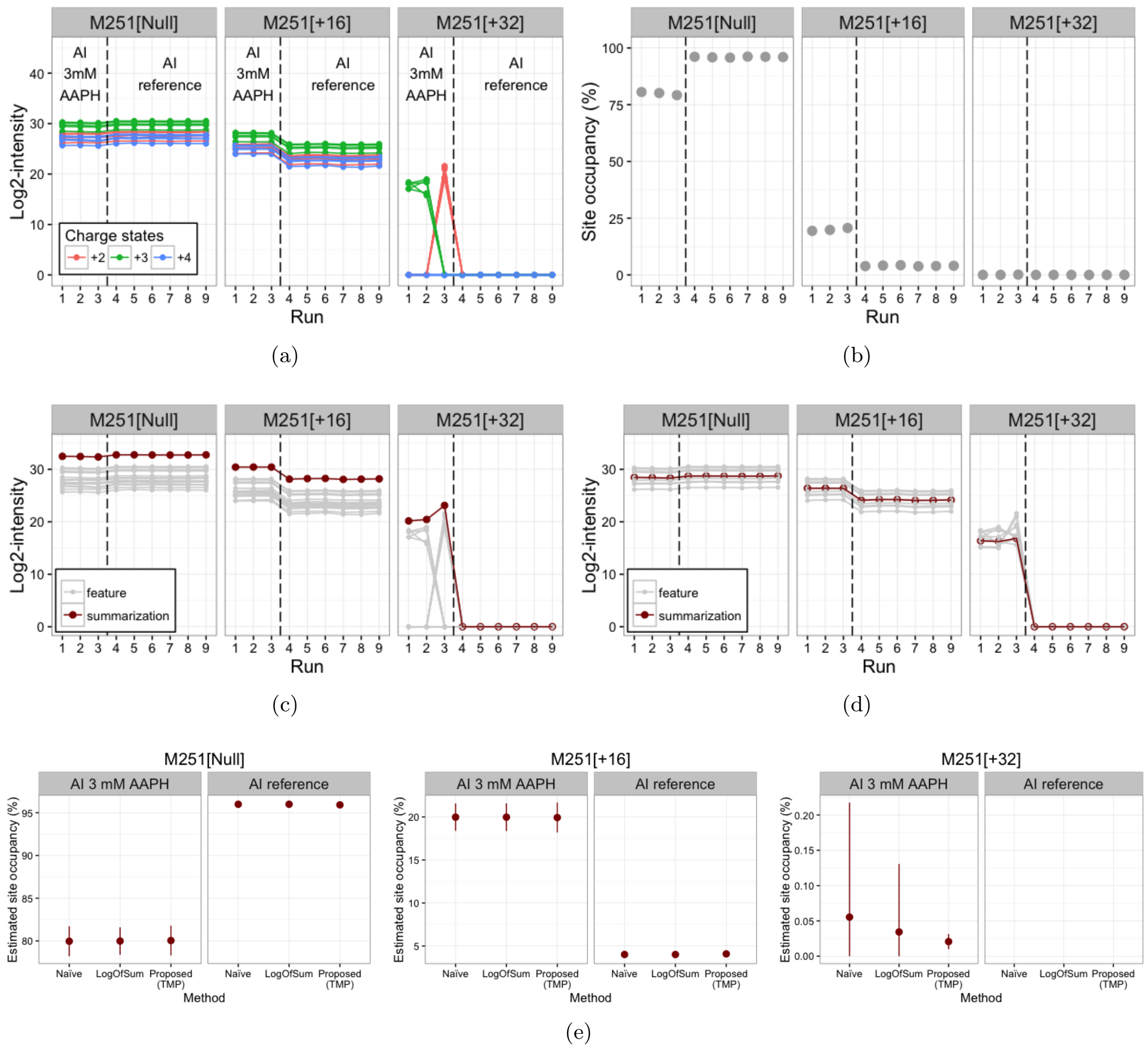

Figure S7: (a) Profile plot of the original features for the 3 forms at site M251 in AI 3 mM AAPH and AI reference samples as in Figure 5a of the main text. (b) Calculated site occupancy of each form in each run. (c) Profile plot with summarization of feature intensities by the LogOfSum method. Summarized intensities are shown in dark red. (d) Profile plot with the TMP summarization of feature intensities. (e) Estimates of site occupancies with 95% confidence intervals by the naïve, LogOfSum and proposed TMP methods.

5 Proposed approach for Goal 2: differential site occupancy

Statistical characterization of differential site occupancy tests the null hypothesis of *no change* against the alternative. There are three considered hypotheses as introduced in Figure 3c in the

main text. They are based on the same linear mixed model as for Goal 1.

5.1 Statistical model for input data, parameter estimation and summarization

Same as for Goal 1 (Section 4.1).

5.2 Model-based inference for Goal 2

The three considered hypotheses used in the differential analysis for Form j at Site i are described below. They differ in the use of the normalizing factor in the denominator.

Hypothesis $H_0^{(1)}$. The hypothesis states that there is no difference in site occupancy (as defined in Eq. (19)) between Conditions k and k' for the form

$$\begin{aligned} H_0^{(1)} &: \phi_{ijk} - \phi_{ijk'} = 0 \\ H_a^{(1)} &: \phi_{ijk} - \phi_{ijk'} \neq 0 \end{aligned} \tag{23}$$

The difference in site occupancy is denoted by $\delta^{(1)}$ and estimated as

$$\hat{\delta}^{(1)} = \hat{\phi}_{ijk} - \hat{\phi}_{ijk'} = \frac{\hat{\mu}_{ijk}}{\hat{\mu}_{i+k}} - \frac{\hat{\mu}_{ijk'}}{\hat{\mu}_{i+k'}}, \tag{24}$$

where $\hat{\phi}_{ijk}$ is as in Eq. (20), and $\hat{\mu}_{ijk}$ is as in Eq. (17). The test statistic is

$$\frac{\hat{\delta}^{(1)}}{\mathrm{SE}(\hat{\delta}^{(1)})} = \frac{\frac{\hat{\mu}_{ijk}}{\hat{\mu}_{i+k}} - \frac{\hat{\mu}_{ijk'}}{\hat{\mu}_{i+k'}}}{\left[\mathrm{SE}^2(\hat{\phi}_{ijk}) + \mathrm{SE}^2(\hat{\phi}_{ijk'})\right]^{1/2}}, \tag{25}$$

where $\mathrm{SE}(\hat{\phi}_{ijk})$ and $\mathrm{SE}(\hat{\phi}_{ijk'})$ can be estimated as in Eq. (21). The test statistic is compared against the t distribution. The degrees of freedom of the t distribution are calculated as in Section 4.2.

Hypothesis $H_0^{(2)}$. The hypothesis states that there is no difference in log-abundance between Conditions k and k' for the form, same as in relative label-free quantification

$$\begin{aligned} H_0^{(2)} &: \log(\mu_{ijk}) - \log(\mu_{ijk'}) = 0 \\ H_a^{(2)} &: \log(\mu_{ijk}) - \log(\mu_{ijk'}) \neq 0 \end{aligned} \tag{26}$$

The difference in log-abundance is denoted by $\delta^{(2)}$ and estimated as

$$\hat{\delta}^{(2)} = \log(\hat{\mu}_{ijk}) - \log(\hat{\mu}_{ijk'}) = \frac{\hat{y}_{ijk+}}{L} - \frac{\hat{y}_{ijk'+}}{L}. \tag{27}$$

The test statistic is

$$\frac{\hat{\delta}^{(2)}}{\mathrm{SE}(\hat{\delta}^{(2)})} = \frac{\hat{\delta}^{(2)}}{\left[\mathrm{SE}^2(\log(\hat{\mu}_{ijk})) + \mathrm{SE}^2(\log(\hat{\mu}_{ijk'}))\right]^{1/2}} = \frac{\frac{1}{L}\hat{y}_{ijk+} - \frac{1}{L}\hat{y}_{ijk'+}}{\left(\frac{1}{L}\hat{\sigma}^2_{\theta,ijk} + \frac{1}{L}\hat{\sigma}^2_{\theta,ijk'}\right)^{1/2}}. \tag{28}$$

The estimate of log-abundance and associated standard error are calculated from the whole-plot model as in Eq. (15). The test statistic is compared against the t distribution.

Hypothesis $H_0^{(3)}$. The hypothesis states that there is no difference in log-abundance between Conditions k and k' for the form, normalized by the reference peptide, same as in relative label-free quantification with a global reference standard

$$\begin{aligned} H_0^{(3)} &: \log\left(\frac{\mu_{ijk}}{\mu_{\mathrm{R}k}}\right) - \log\left(\frac{\mu_{ijk'}}{\mu_{\mathrm{R}k'}}\right) = 0 \\ H_a^{(3)} &: \log\left(\frac{\mu_{ijk}}{\mu_{\mathrm{R}k}}\right) - \log\left(\frac{\mu_{ijk'}}{\mu_{\mathrm{R}k'}}\right) \neq 0 \end{aligned} \tag{29}$$

The difference in log-abundance is denoted by $\delta^{(3)}$ and estimated as

$$\hat{\delta}^{(3)} = \frac{\hat{y}_{ijk+} - \hat{y}_{\mathrm{R}k+}}{L} - \frac{\hat{y}_{ijk'+} - \hat{y}_{\mathrm{R}k'+}}{L}, \tag{30}$$

The estimation and testing for $H_0^{(3)}$ are based on the same approach as for $H_0^{(2)}$. The key difference is the use of reference peptide for $H_0^{(3)}$, i.e., in the whole-plot model (Eq. (15)), the modeled log-intensity is $(\hat{y}_{ijkl} - \hat{y}_{\mathrm{R}kl})$, rather than $\hat{y}_{ijkl}$.

6 Proposed approach for Goal 3: combined occupancy estimation

Data from non-mass spectrometric methods provide orthogonal evidence to evaluate the ability of our proposed statistical methods to accurately estimate site occupancy using LC-MS/MS data. These methods, however, are not always site-specific. To use orthogonal methods as an external benchmark of performance, extended methodology combining the quantitative information across multiple sites is needed. The combined occupancy of Form j in Condition k can be approximated by

$$\phi_{jk}^{(c)} = \frac{\sum_{i=1}^{I} \mu_{ijk}}{\sum_{i=1}^{I} \sum_{j'=1}^{J} \mu_{ij'k}} = \frac{\mu_{+jk}}{\mu_{++k}}. \tag{31}$$

The approximation gives equal weight to each site for combining the abundance, assuming that features (peptide ions) at different sites share an identical ionization efficiency.

6.1 Statistical model for input data, parameter estimation and summarization

Same as for Goal 1 (Section 4.1).

6.2 Model-based inference for Goal 3

In a similar way as for Goal 1 (Section 4.2), the combined occupancy can be estimated by the estimates of the abundance of the forms across sites ($i = 1, \ldots, I$)

$$\hat{\phi}_{jk}^{(c)} = \sum_{i=1}^{I} \frac{\hat{\mu}_{ijk}}{\hat{\mu}_{++k}} = \frac{\hat{\mu}_{+jk}}{\hat{\mu}_{++k}}, \tag{32}$$

and the SE of the estimate is computed using the delta method

$$\mathrm{SE}(\hat{\phi}_{jk}^{(c)}) = \left[\sum_{i=1}^{I} \sum_{j'=1}^{J} \left(\frac{\partial \hat{\phi}_{jk}^{(c)}}{\partial \log_2(\hat{\mu}_{ij'k})} \right)^2 \cdot \mathrm{SE}\left(\log_2(\hat{\mu}_{ij'k}) \right)^2 \right]^{1/2}, \tag{33}$$

where

$$\frac{\partial \hat{\phi}_{jk}^{(c)}}{\partial \log_2(\hat{\mu}_{ij'k})} = \begin{cases} (\hat{\mu}_{++k})^{-2} \cdot \log 2 \cdot \hat{\mu}_{ijk} \cdot (\hat{\mu}_{++k} - \hat{\mu}_{+jk}) & \text{if } j' = j \\ -(\hat{\mu}_{++k})^{-2} \cdot \log 2 \cdot \hat{\mu}_{ij'k} \cdot \hat{\mu}_{+jk} & \text{if } j' \neq j \end{cases}$$

Finally, the $100 \times (1 - \alpha)$ percent confidence interval of the estimate is given by

$$\hat{\phi}_{jk}^{(c)} \pm t_{df,\alpha/2} \mathrm{SE}(\hat{\phi}_{jk}^{(c)}), \tag{34}$$

where $t_{df,\alpha/2}$ is the $100 \times (1 - \alpha/2)$ percentile of the t distribution with degrees of freedom calculated as in Section 4.2. While being introduced for combining information of a form across multiple sites, this approach is applicable to combinations involving multiple forms and sites.

7 Computer simulation

The proposed statistical method is evaluated for site occupancy estimation (Goal 1) and detection of differential site occupancy (Goal 2) in comparison with the naïve method using simulation data.

7.1 Computer simulation for Goal 1

Setting in the simulation for site occupancy estimation:

- One condition
- Three forms: 5%, 20%, 75%
- Number of replicates: 1, 3, 5
- Mean of log-intensity: 25
- Standard deviation of log-intensity: 0.2
- Number of realizations: 1000

7.2 Computer simulation for Goal 2

Setting in the simulation for differential site occupancy:

- Two conditions: C_0, C_1
- Three forms:
  - With no change: 5%, 20%, 75% in C_0; 5%, 20%, 75% in C_1
  - With change: 5%, 20%, 75% in C_0; $(5 + \delta)$%, 20%, $(75 - \delta)$% in C_1
- Difference in site occupancy δ: 5%, 10%, 15%
- Number of replicates: 3, 5
- Mean of log-intensity: 25
- Standard deviation of log-intensity: 0.2
- Number of realizations: 1000

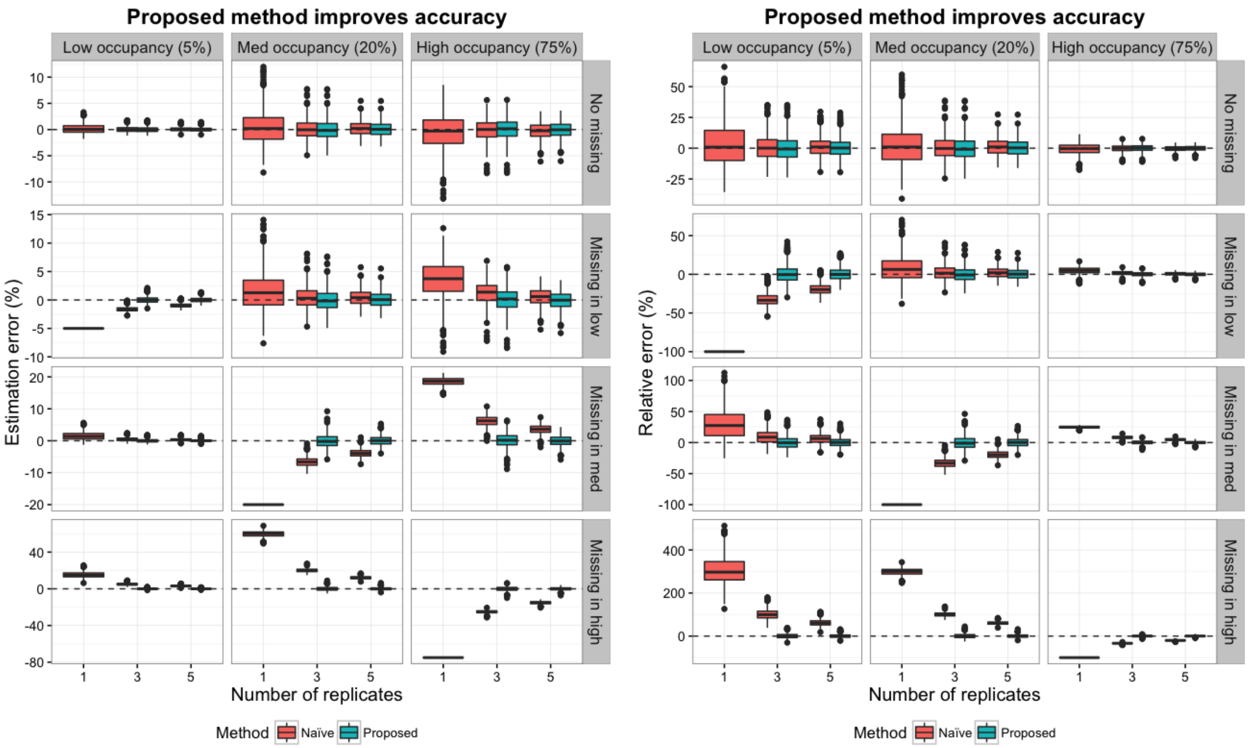

(a) (b)

Figure S8: Results of simulation analysis for Goal 1 in terms of estimation accuracy. (a) Boxplot of the estimation errors. (b) Boxplot of the relative errors of the estimates (i.e., estimation errors divided by true values).

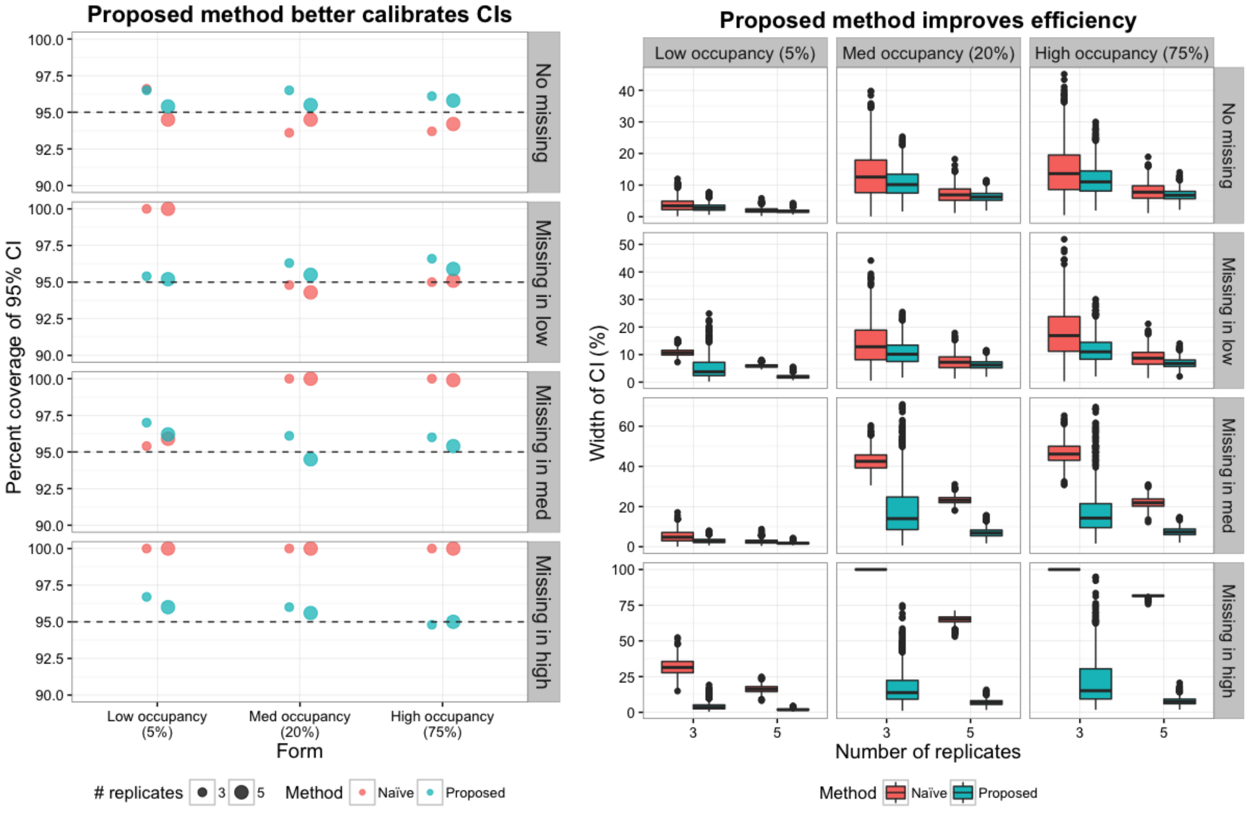

(a) (b)

Figure S9: Results of simulation analysis for Goal 1 in terms of inference. (a) Percent coverage of 95% confidence interval. (b) Boxplot of the widths of 95% confidence intervals.

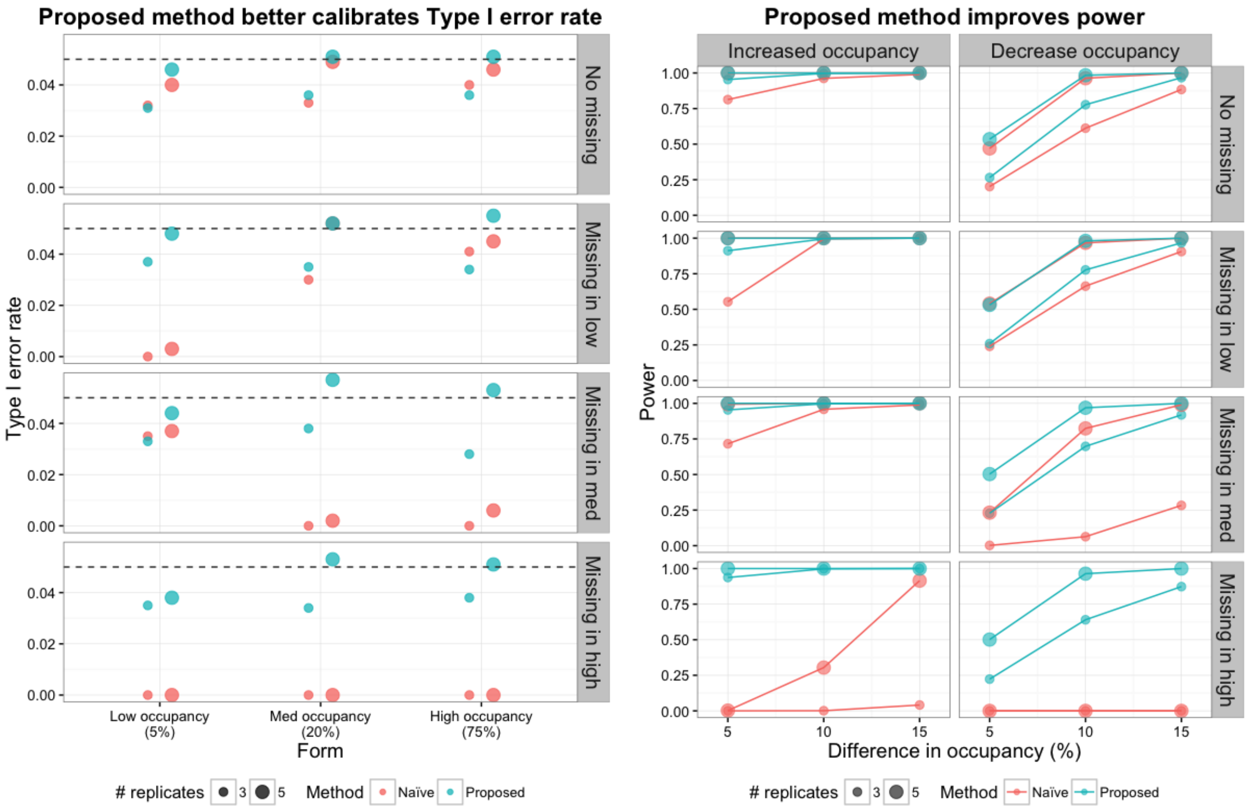

(a) (b)

Figure S10: Results of simulation analysis for Goal 2. (a) Type I error rate. (b) Statistical power in detecting differential site occupancies.

8 Details for the example: site occupancy estimation for N-linked glycoforms

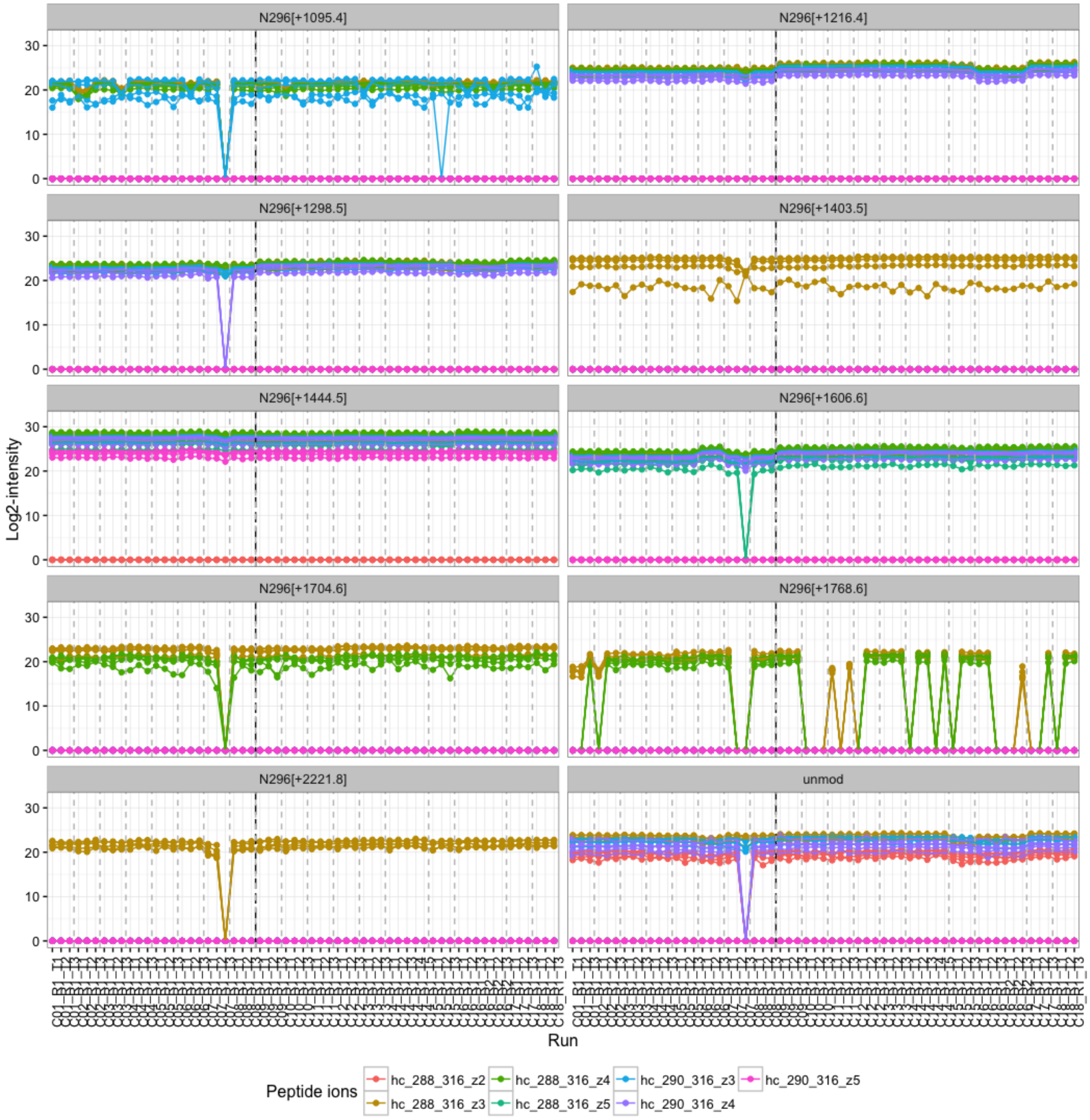

Figure S11: Profile plot of the peptide features for the 10 forms at site N296. Replicate runs of the same condition are arranged together; different conditions are separated by gray dashed lines. The black dashed line separates the 8 ADC samples (left) and the 10 AI samples (right).

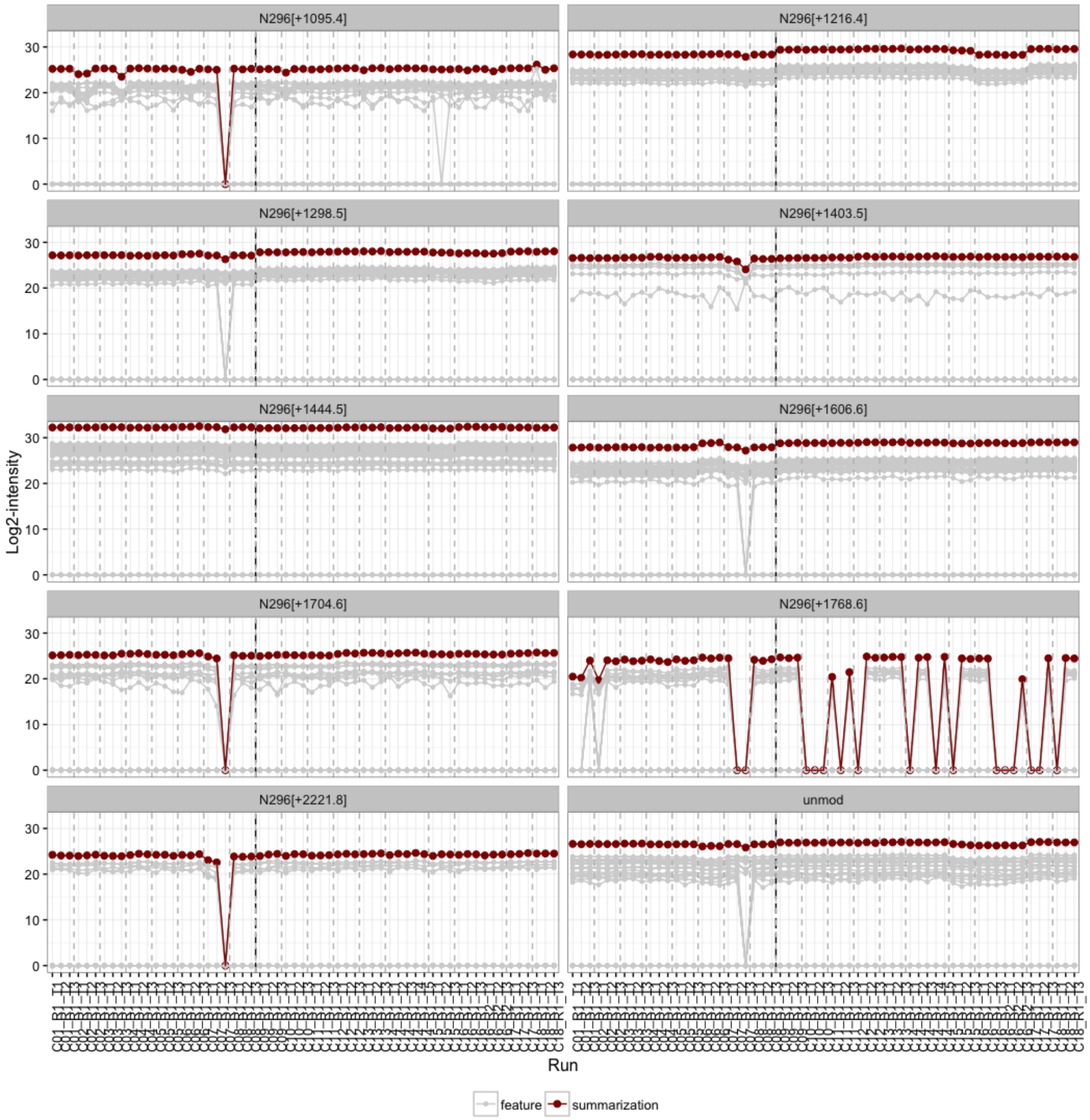

Figure S12: Profile plot with summarization of feature intensities by the LogOfSum method at site N296. Summarized intensities are shown in dark red. Feature selection is not done with this method and different sets of features are used for summarization in different forms and runs.

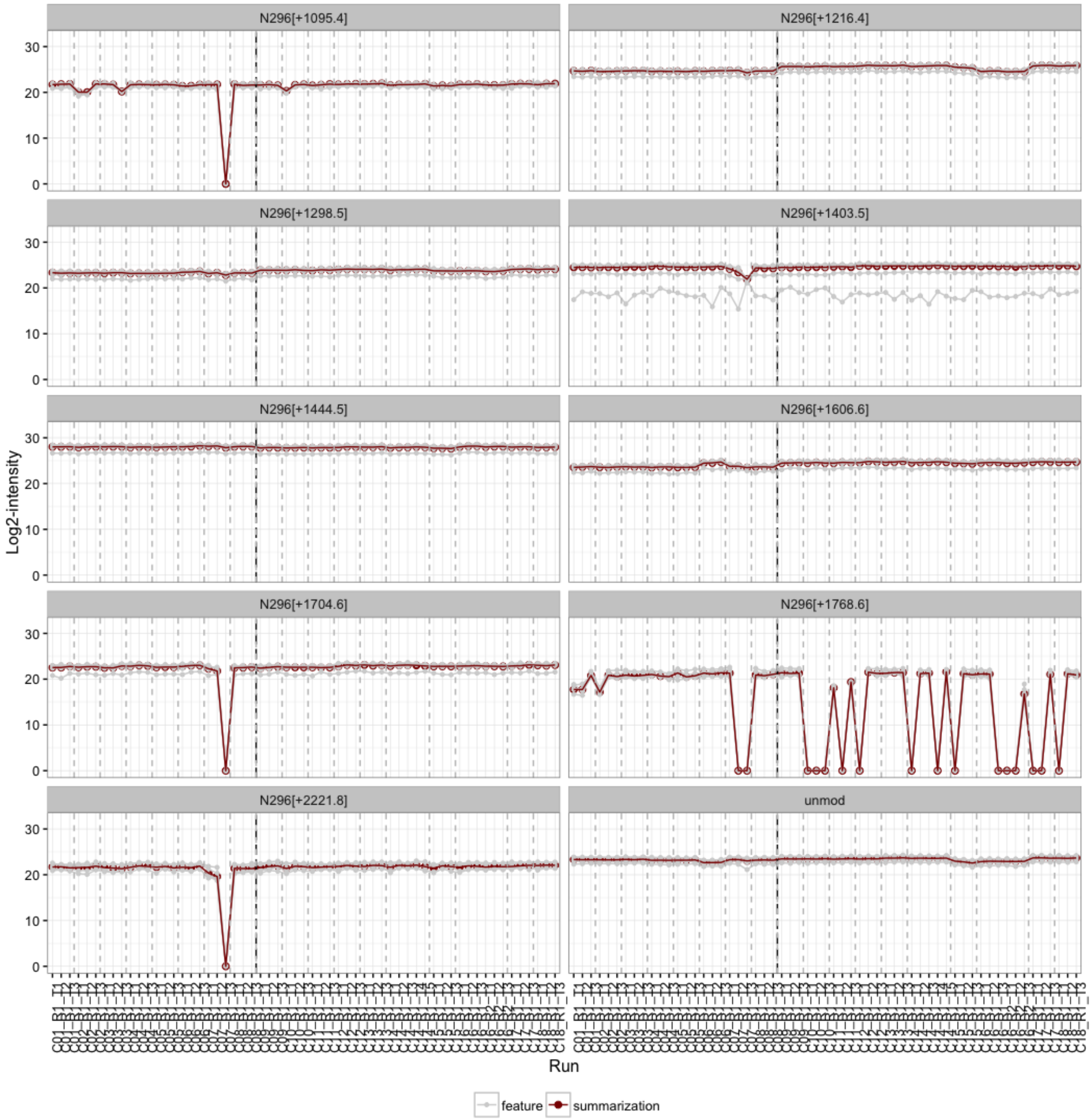

Figure S13: Profile plot with summarization of feature intensities by the proposed TMP method at site N296. Summarized intensities are shown in dark red. The same features (triply charged peptide from heavy chain [288, 316]) are used for summarization for all the 10 forms. Whereas the confounding due to different ionization efficiencies of features is eliminated, most features are not selected for the summarization in this example due to their missingness in some forms.

Table S3: Estimation of site occupancies for N-linked glycoforms in the AI Reference and ADC Reference using the naïve, LogOfSum and proposed TMP methods. The results for the AI Reference and ADC Reference should be similar as the conjugation of linker drugs is only expected to affect the characteristics at drug modification sites. The results are compared with those from orthogonal experiments using 2-AB HILIC and CE/LIF. The TMP method gives a lower estimate for the most abundant A2G0F glycoform than other methods and orthogonal data. This is because many features that are absent in other low abundant forms are removed in the feature selection step, including those with high intensities in A2G0F. Using the selected features (triply charged peptide from heavy chain [288, 316]) alone may not be sufficient for accurate inference of the abundances of all forms. For a site with many modification forms as in this example, there is a tradeoff between the number of selected features and the consistency across forms. Alternative definitions using reference peptides may be better suited.

Mod.	Form	2AB-HILIC	CE/LIF	Naïve		LogOfSum		Proposed (TMP)	
		AI-Ref	AI-Ref	ADC-Ref	AI-Ref	ADC-Ref	AI-Ref	ADC-Ref	AI-Ref
A1G0	N296[+1095.4]	0.515	NA	0.457	0.520	0.450	0.515	0.723	0.835
M5	N296[+1216.4]	4.645	0.9	5.069	4.831	5.069	4.832	6.585	6.275
A1G0F	N296[+1241.5]	1.859	2.1	NA	NA	NA	NA	NA	NA
A2G0	N296[+1298.5]	2.598	2.1	2.581	3.005	2.582	3.005	2.944	3.397
M6	N296[+1378.5]	0.247	NA	NA	NA	NA	NA	NA	NA
A1G1F	N296[+1403.5]	NA	0.2	1.569	1.707	1.569	1.707	6.413	6.818
A2G0F	N296[+1444.5]	81.087	82.1	81.123	80.601	81.131	80.604	72.231	71.624
A2G1	N296[+1460.5]	0.314	NA	NA	NA	NA	NA	NA	NA
A2G0M4									
A3G0	N296[+1501.6]	0.000	NA	NA	NA	NA	NA	NA	NA
M7	N296[+1540.5]	0.084	NA	NA	NA	NA	NA	NA	NA
A2G1F	N296[+1606.6]	7.100	11.6	6.871	7.065	6.872	7.065	6.047	6.051
A3G0F	N296[+1647.6]	0.223	NA	NA	NA	NA	NA	NA	NA
M8	N296[+1702.6]	0.239	NA	NA	NA	NA	NA	NA	NA
A4G0	N296[+1704.6]	NA	NA	0.666	0.652	0.666	0.651	1.957	1.901
A2G2F	N296[+1768.6]	0.302	0.5	0.354	0.113	0.353	0.118	0.627	0.209
A2S1G2F	N296[+2221.8]	0.000	0.1	0.277	0.299	0.276	0.299	0.833	0.908
NA	Unmodified	NA	NA	1.034	1.205	1.033	1.204	1.638	1.981

9 Details for the example: differential site occupancy

9.1 Differential site occupancy based on $H_0^{(1)}$

Table S4: Complete results of the differential site occupancy analysis as in Table 1 of the main text. The analysis is based on the hypotheses $H_0^{(1)}$. The naïve, LogOfSum and proposed TMP methods are compared. Performances are evaluated based on sensitivity, specificity, and positive predictive value (PPV). Sensitivity is calculated as TP/(TP+FN), where TP is the number of true positives and FN is the number of false negatives. Specificity is calculated as TN/(FP+TN), where FP is the number of false positives and TN is the number of true negatives. PPV is calculated as TP/(TP+FP).

Method	Positive control		Negative control		Sensitivity	Specificity	PPV
	TP	FN	FP	TN			
Naïve	28	6	13	49	0.824	0.790	0.683
LogOfSum	28	6	6	55	0.824	0.902	0.824
Proposed (TMP)	28	6	5	56	0.824	0.918	0.848

9.2 Differential site occupancy based on $H_0^{(2)}$ and $H_0^{(3)}$

Table S5: Results of differential site occupancy analysis at the FDR cutoff 0.05 in the comparisons for positive and negative controls. The analysis was based on the hypotheses $H_0^{(2)}$ and $H_0^{(3)}$. Performances of the LogOfSum and proposed TMP methods were evaluated based on sensitivity, specificity, and PPV.

Hypothesis	Method	Positive control		Negative control		Sensitivity	Specificity	PPV
		TP	FN	FP	TN			
$H_0^{(2)}$	LogOfSum	22	12	10	51	0.647	0.836	0.688
$H_0^{(2)}$	Proposed (TMP)	23	11	9	52	0.676	0.852	0.719
$H_0^{(3)}$	LogOfSum	21	13	12	49	0.618	0.803	0.636
$H_0^{(3)}$	Proposed (TMP)	22	12	13	48	0.647	0.787	0.629

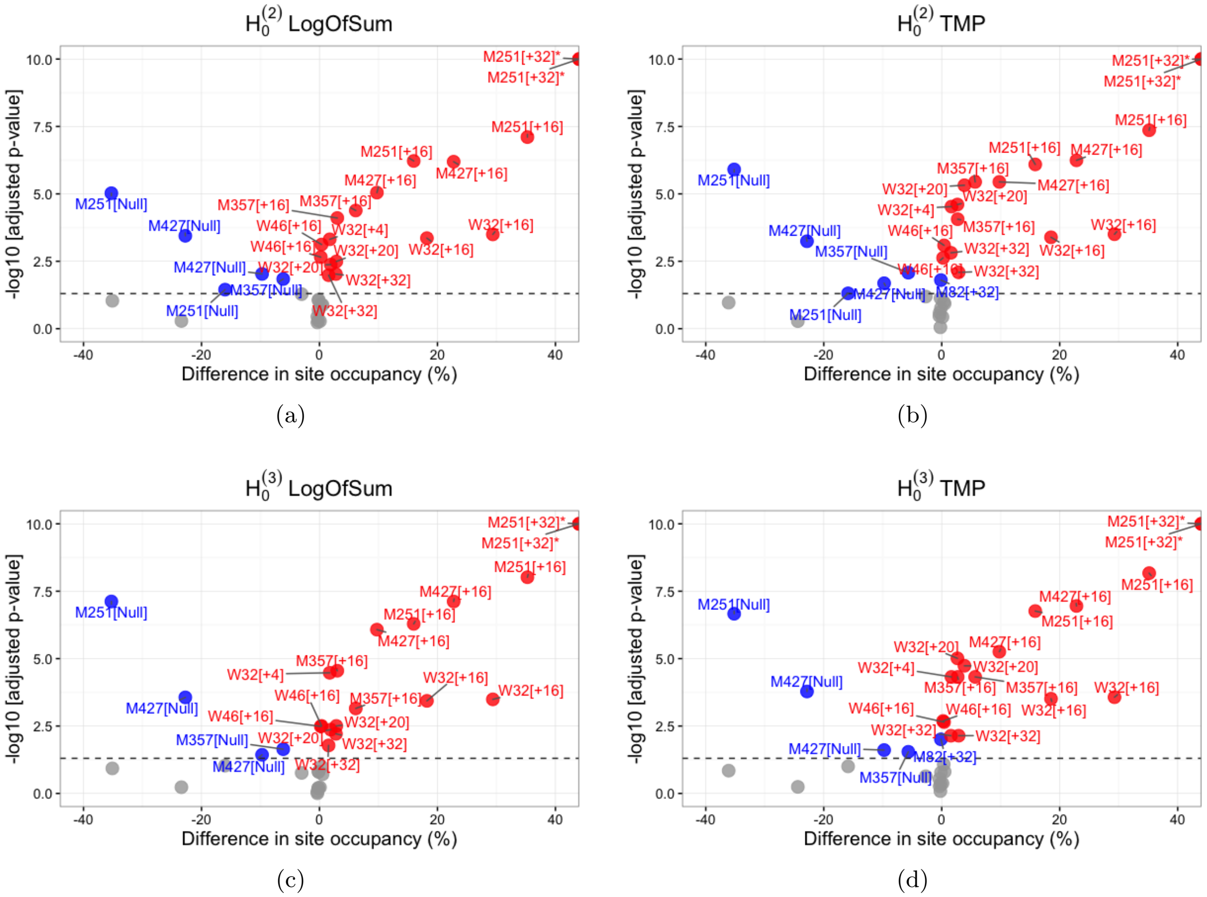

Figure S14: Comparison of sites at Fab and Fc between AI 3mM/10mM AAPH versus AI Reference as positive control. The differential analysis was based on the hypotheses $H_0^{(2)}$ (upper panel) and $H_0^{(3)}$ (lower panel). Each testing was performed by applying the LogOfSum and proposed TMP methods. Results of the analysis are summarized in a volcano plot showing the practical significance (change in site occupancy) and the statistical significance (-$\log_{10}$[adjusted p-value]). The horizontal line represents the 0.05 FDR cutoff. In this example, testing based on $H_0^{(2)}$ or $H_0^{(3)}$ (using either the LogOfSum or proposed TMP method) resulted in similar results.

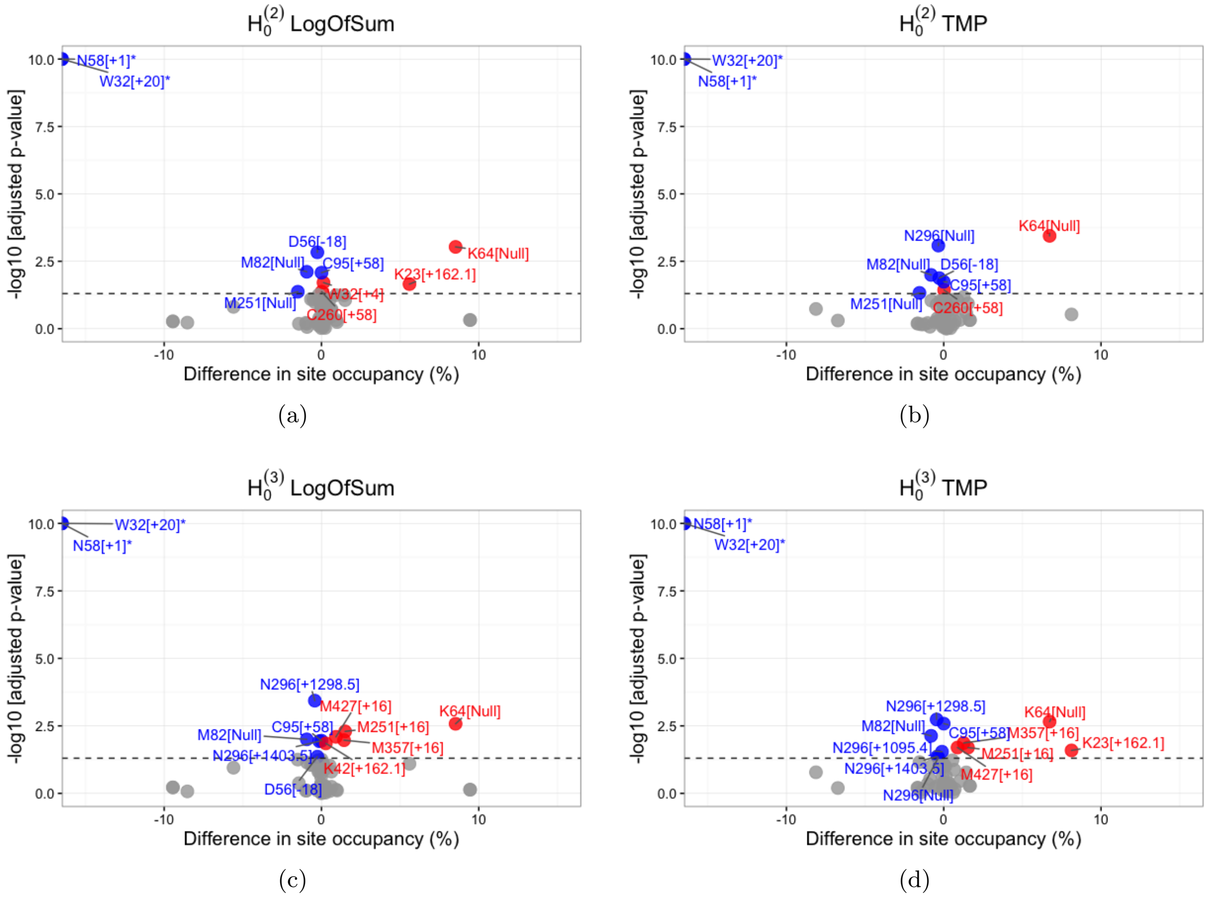

Figure S15: Comparison between ADC Reference versus AI Reference excluding the sites for drug modifications as negative control. The differential analysis was based on the hypotheses $H_0^{(2)}$ (upper panel) and $H_0^{(3)}$ (lower panel). Each testing was performed by applying the LogOfSum and proposed TMP methods. Results of the analysis are summarized in a volcano plot showing the practical significance (change in site occupancy) and the statistical significance (-$\log_{10}$[adjusted p-value]). The horizontal line represents the 0.05 FDR cutoff. In this example, testing based on $H_0^{(2)}$ (using either the LogOfSum or proposed TMP method) resulted in slightly better specificity than $H_0^{(3)}$.

9.3 Differential site occupancy analysis with small sample sizes

The LC-MS/MS runs of the AI 3 mM AAPH, AI 10 mM AAPH, AI reference and ADC reference samples (used as positive and negative controls) were resampled to a size of 2 or 3 (without replacement) per condition prior to the differential analysis (based on $H_0^{(1)}$).

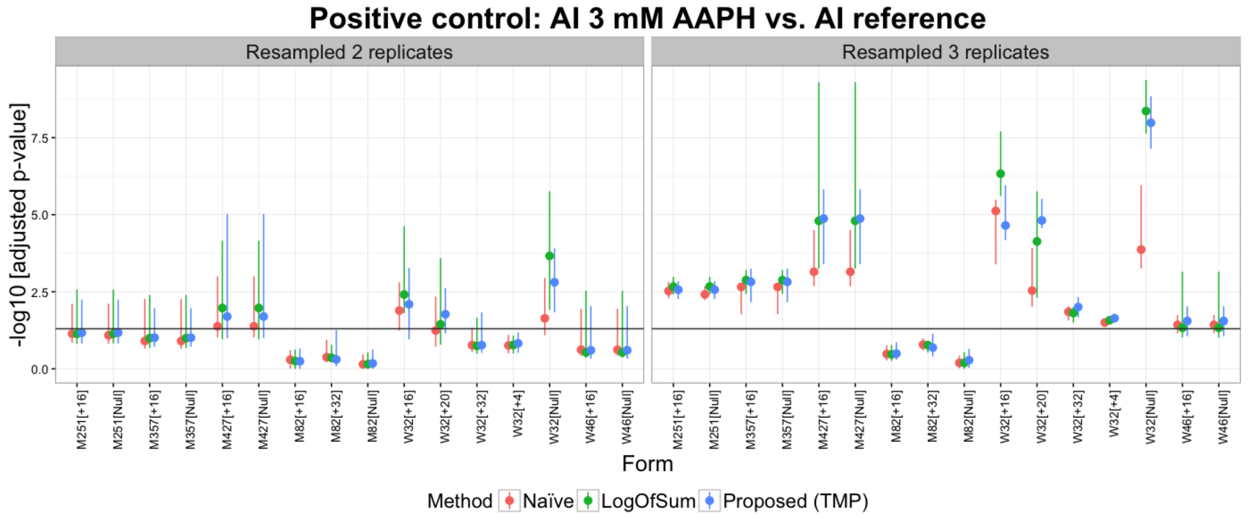

(a)

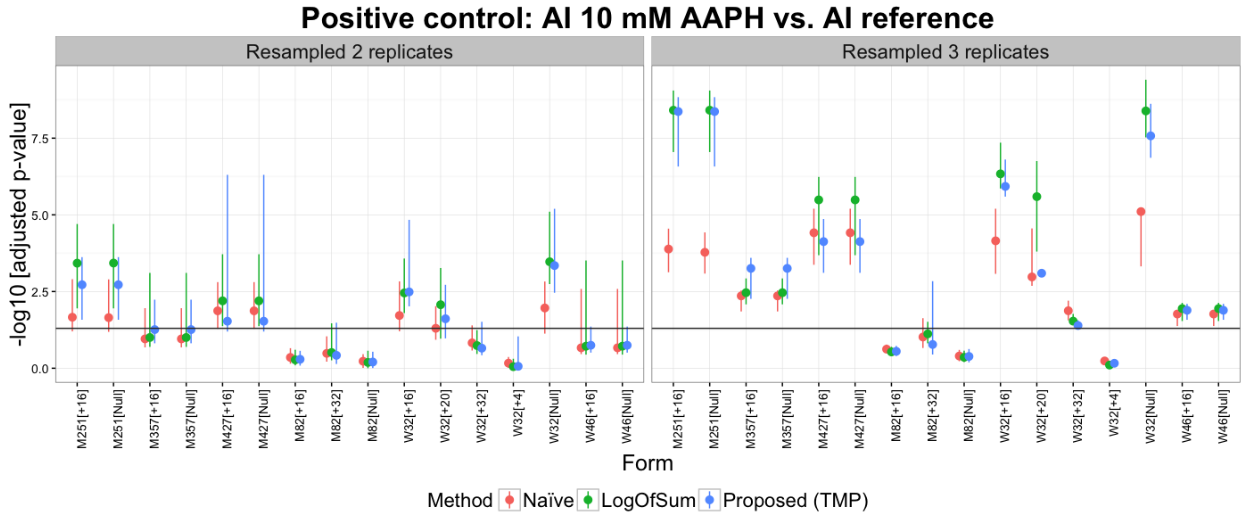

(b)

Figure S16: Statistical significance of the testing based on $H_0^{(1)}$ for the positive control using subsets of the data. (a) AI 3 mM AAPH versus AI reference. (b) AI 10 mM AAPH versus AI reference. The statistical significance is represented as the FDR-adjusted p-values on the negative log10 scale.

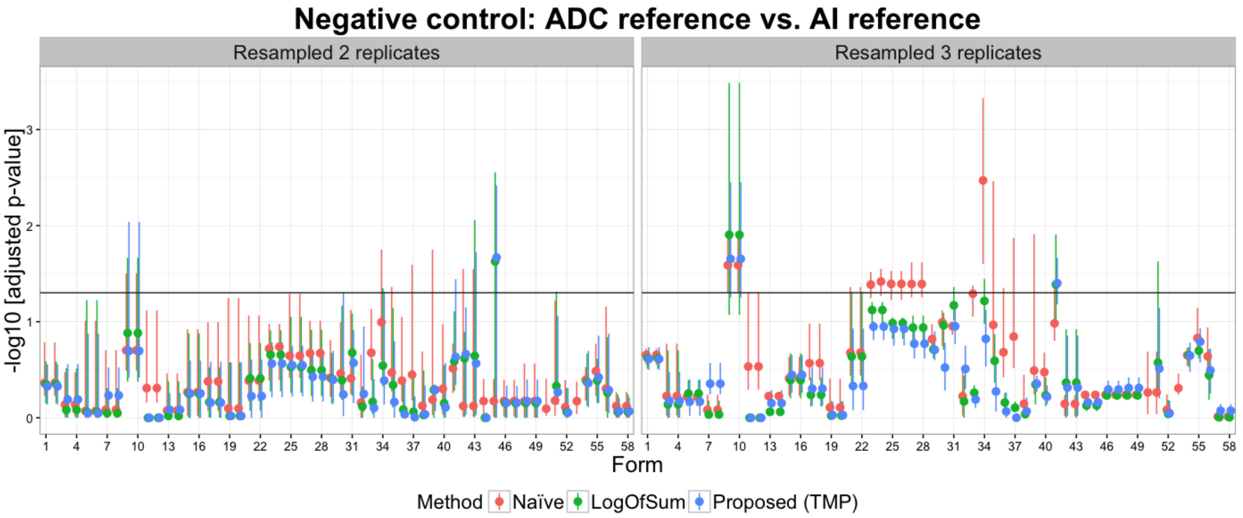

Figure S17: Statistical significance of the testing based on $H_0^{(1)}$ for the negative control (ADC reference versus AI reference) using subsets of the data. The statistical significance is represented as the FDR-adjusted p-values on the negative log10 scale. The list of compared forms is shown in Table S6.

Table S6: List of compared forms for the negative controls.

Forms 1–5	C21[+58]	C21[Null]	C260[+58]	C260[Null]	C424[+58]
Forms 6–10	C424[Null]	D29[-18]	D29[Null]	D56[-18]	D56[Null]
Forms 11–15	K106[-1]	K106[Null]	K152[+162.1]	K152[Null]	K23[+162.1]
Forms 16–20	K23[Null]	K42[+162.1]	K42[Null]	K446[-128.1]	K446[Null]
Forms 21–25	K64[+162.1]	K64[Null]	M251[+16]	M251[Null]	M357[+16]
Forms 26–30	M357[Null]	M427[+16]	M427[Null]	M82[+16]	M82[+32]
Forms 31–35	M82[Null]	N296[+1095.4]	N296[+1216.4]	N296[+1298.5]	N296[+1403.5]
Forms 36–40	N296[+1444.5]	N296[+1606.6]	N296[+1704.6]	N296[+1768.6]	N296[+2221.8]
Forms 41–45	N296[Null]	N37[+1]	N37[Null]	N383[+1]	N383[Null]
Forms 46–50	N388[+1]	N388[Null]	N389[+1]	N389[Null]	N58[+1]
Forms 51–55	N58[Null]	W32[+16]	W32[+20]	W32[+32]	W32[+4]
Forms 56–58	W32[Null]	W46[+16]	W46[Null]		

Table S7: Results of differential site occupancy analysis for positive and negative controls. Number of replicates was resampled to 1 (unreplicated), 2 and 3. For unreplicated data, changes in site occupancy between conditions were considered significant when they exceeded a 5% cutoff, where significant uncertainty was observed. For the other comparisons (with > 1 replicates), hypothesis testing based on $H_0^{(1)}$ was carried out with the FDR cutoff set at 0.05. Performances of the proposed TMP method and naïve method were compared based on average sensitivity, average specificity, and range of PPV over resampled replicates. With small sample sizes, the proposed method improved the overall performance in the differential analysis. Also, the proposed method gave more stable results.

Method	Size	Average sensitivity	Average specificity	Range of PPV
5% cutoff	1	0.416	0.930	(0.583, 0.875)
Naïve	2	0.349	0.989	(0.786, 1.000)
Naïve	3	0.773	0.841	(0.676, 0.862)
Proposed (TMP)	2	0.423	0.980	(0.769, 1.000)
Proposed (TMP)	3	0.761	0.952	(0.885, 0.962)

10 Details for the example: combined occupancy estimation for oxidative modifications

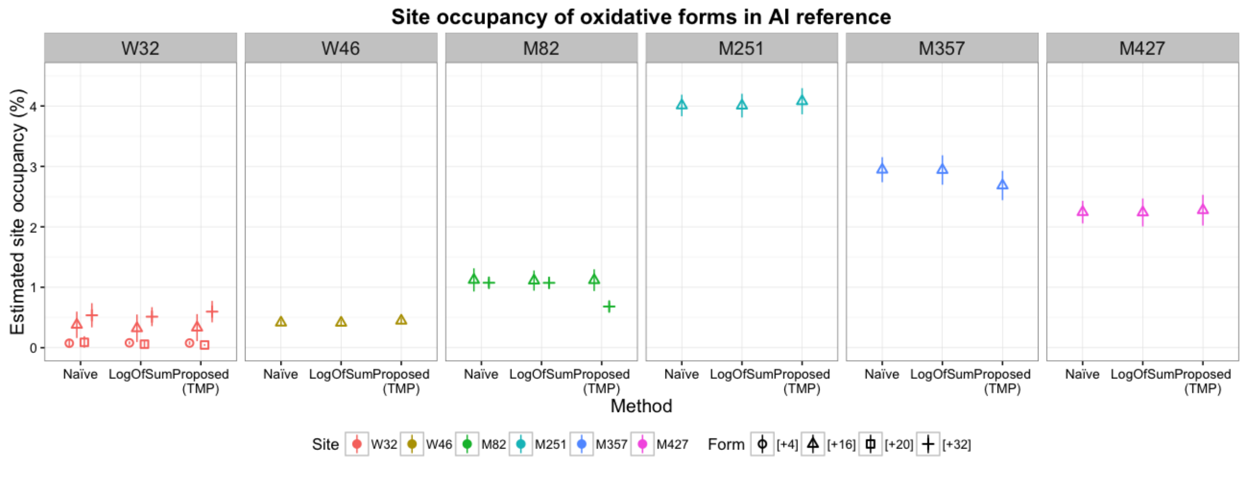

(a)

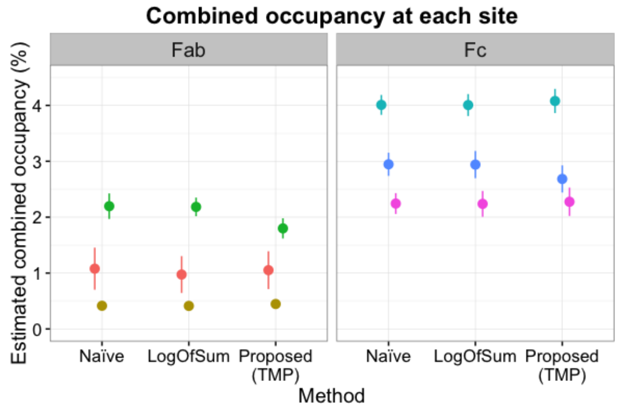

(b)

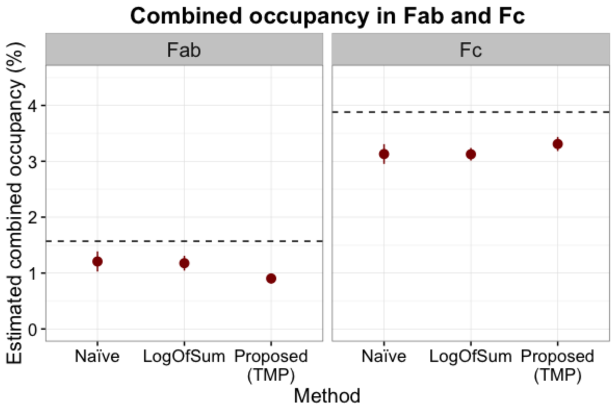

(c)

Figure S18: Estimation of site/combined occupancy for oxidative modifications. (a) Site occupancy estimation. (b) Combined occupancy estimation for sites in Fab and Fc. (c) Combined occupancy estimation in Fab and Fc

11 Evaluation of model assumptions

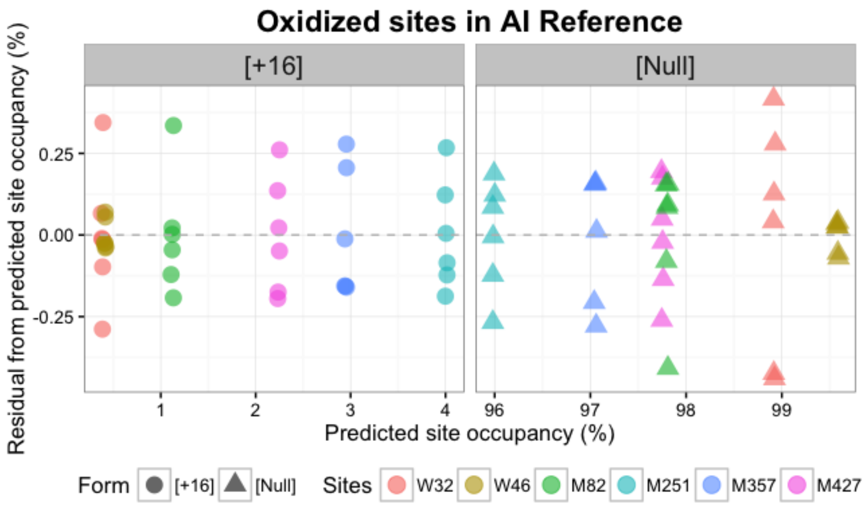

(a)

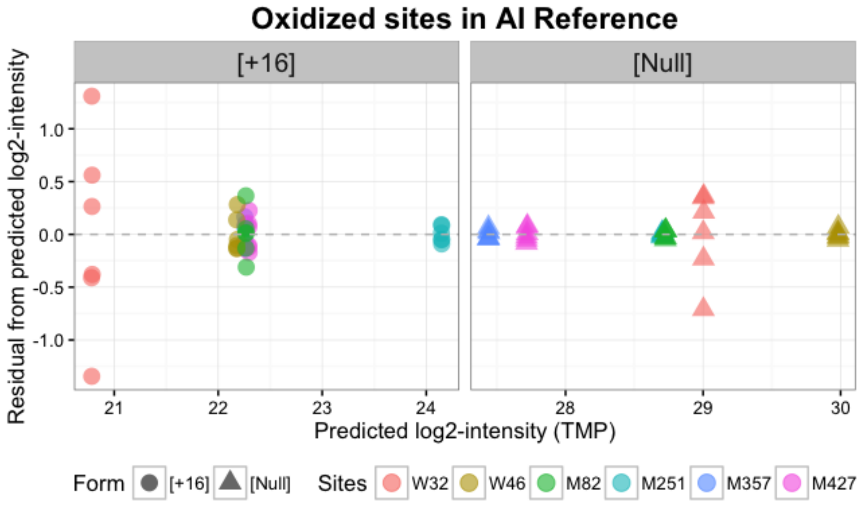

(b)

Figure S19: Residual plots for (a) site occupancy summarized in each run, and (b) log2-intensity of feature with the measurements for a [+16] oxidative form and the unmodified form at six oxidized sites in AI reference sample. Both methods do not assume constant variance, which seems reasonable from the plots.

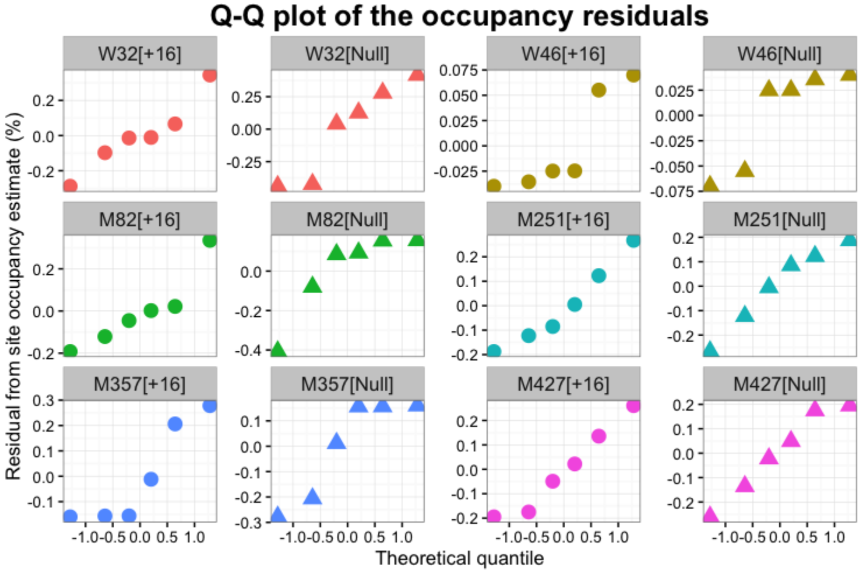

(a)

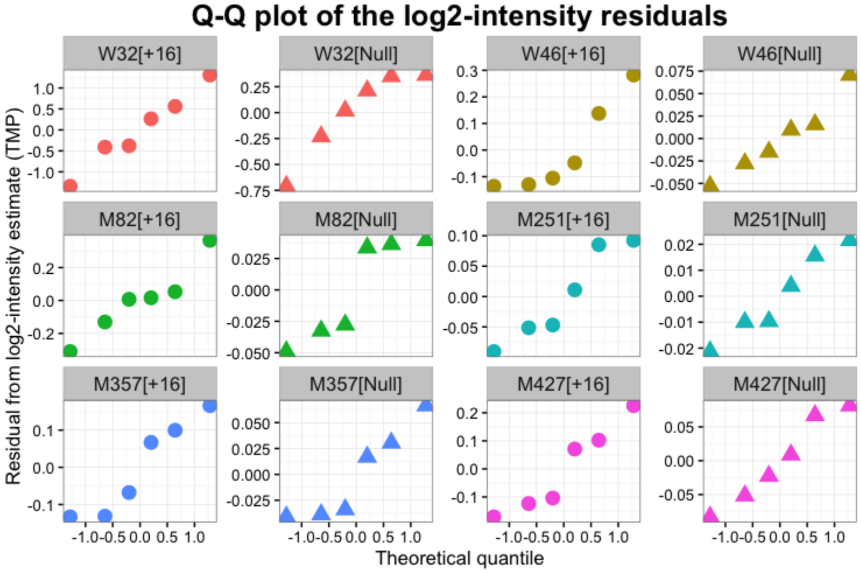

(b)

Figure S20: Quantile-quantile (Q-Q) plots for (a) site occupancy summarized in each run, and (b) log2-intensity of feature. With few replicates, the residuals on both scales do not completely agree with the normality assumption in all the forms. Jump discontinuities are observed in forms W46[+16] and W46[Null] for the occupancy residuals due to the nature of the summary (defined in a bounded space).

References

1. Meena Choi, Ching-Yun Chang, Timothy Clough, Daniel Broudy, Trevor Killeen, Brendan MacLean, and Olga Vitek. MSstats: an R package for statistical analysis of quantitative mass spectrometry-based proteomic experiments. *Bioinformatics*, 30(17):2524–2526, 2014.

2. David S. Johnson. Approximation algorithms for combinatorial problems. *Journal of Computer and System Sciences*, 9(3):256–278, 1974.

3. Yuliya Karpievitch, Jeff Stanley, Thomas Taverner, Jianhua Huang, Joshua N. Adkins, Charles Ansong, Fred Heffron, Thomas O. Metz, Wei-Jun Qian, Hyunjin Yoon, Richard D. Smith, and Alan R. Dabney. A statistical framework for protein quantitation in bottom-up MS-based proteomics. *Bioinformatics*, 25(16):2028–2034, 2009.

4. Douglas C. Montgomery. *Design and Analysis of Experiments*. John Wiley & Sons, Inc., Hoboken, NJ, 4 edition, 2005.

5. Carmen D. Tekwe, Raymond J. Carroll, and Alan R. Dabney. Application of survival analysis methodology to the quantitative analysis of LC-MS proteomics data. *Bioinformatics*, 28(15):1998–2003, 2012.

6. John W. Tukey. *Exploratory Data Analysis*. Addison-Wesley, Hoboken, NJ, 1977.

7. Xuan Wang, Gordon A. Anderson, Richard D. Smith, and Alan R. Dabney. A hybrid approach to protein differential expression in mass spectrometry-based proteomics. *Bioinformatics*, 28(12):1586–1591, 2012.

Statistical characterization of therapeutic protein modifications

This markdown file illustrates the propsoed workflow for statistical characterization of therapeutic protein modifications using LC-MS/MS data. We distinguish three statistical goals for therapeutic protein characterization:

1. Estimation of site occupancy of modifications in one condition.
2. Detection of differential site occupancy between conditions.
3. Estimation of combined site occupancy across multiple modification sites.

The proposed approach is compared to a *naïve* data analysis method via comparison with orthogonal experimental measurements of total oxidation and evaluation in positive and negative controls.

Setup

Load required packages

```
# load packages
library(readr)
library(tidyr)
library(dplyr)
library(broom)
library(survival)
library(ggplot2)
library(ggrepel)
```

Load functions (`protchar-methods.R`, code provided in the end) and data (`occ_example.csv`)

```
dta_raw <- read_csv("occ_example.csv")
source("protchar-methods.R")
```

Select and rename relevant columns:

- `seq_pep`: unmodified peptide sequence
- `seq_mod`: modified peptide sequence
- `z_ms1`: charge state of precursor ion
- `iso_idx`: index of isotope
- `cond`: condition
- `run`: LC-MS/MS run
- `auc`: area under chromatographic peak

```
dta_exp <- tbl_df(dta_raw) %>%
    select(seq_pep = PeptideSequence,
           seq_mod = PeptideModifiedSequence,
           z_ms1 = PrecursorCharge,
           iso_idx = IsotopeDistIndex,
           cond = Condition,
           run = Run,
           auc = Area)
```

```
head(dta_exp) %>% as.data.frame()
```

```
##     seq_pep   seq_mod z_ms1 iso_idx            cond                 run
## 1 PLTFGQGTK PLTFGQGTK     1       0 ADC-AAPHControl ADC-AAPHControl_T1
## 2 PLTFGQGTK PLTFGQGTK     1       1 ADC-AAPHControl ADC-AAPHControl_T1
## 3 PLTFGQGTK PLTFGQGTK     2       0 ADC-AAPHControl ADC-AAPHControl_T1
## 4 PLTFGQGTK PLTFGQGTK     2       2 ADC-AAPHControl ADC-AAPHControl_T1
## 5 PLTFGQGTK PLTFGQGTK     1       2 ADC-AAPHControl ADC-AAPHControl_T1
## 6 PLTFGQGTK PLTFGQGTK     2       1 ADC-AAPHControl ADC-AAPHControl_T1
##       auc
## 1  946703
## 2  432972
## 3 7853827
## 4 1090276
## 5  122347
## 6 3806287
```

Data manipulation and transformation

Annotation of condition ID and run ID

```
df_design <- dta_exp %>%
    distinct(cond, run)

cond_from <- sort(unique(df_design$cond))
cond_to <- paste0("C", sprintf("%02d", 1:length(unique(cond_from))))
cond_key <- data_frame(name = cond_from, id = cond_to)

df_design <- df_design %>%
    mutate(cond_id = plyr::mapvalues(cond, from = cond_key$name, to = cond_key$id)) %>%
    rowwise() %>%
    mutate(run_id = paste(c(cond_id, unlist(strsplit(run, "[_]"))[2]), collapse = "_")) %>%
    ungroup()

dta_exp <- dta_exp %>%
    mutate(z_iso = paste0("z", z_ms1, "_", iso_idx),
           run_id = plyr::mapvalues(run, from = df_design$run, to = df_design$run_id),
           cond_id = plyr::mapvalues(run, from = df_design$run, to = df_design$cond_id))
```

Data frame for the experimental design

```
head(df_design) %>% as.data.frame()
```

```
##              cond                run cond_id run_id
## 1 ADC-AAPHControl ADC-AAPHControl_T1     C01 C01_T1
## 2 ADC-AAPHControl ADC-AAPHControl_T2     C01 C01_T2
## 3 ADC-AAPHControl ADC-AAPHControl_T3     C01 C01_T3
## 4  ADC-AAPHStress  ADC-AAPHStress_T1     C02 C02_T1
## 5  ADC-AAPHStress  ADC-AAPHStress_T2     C02 C02_T2
## 6  ADC-AAPHStress  ADC-AAPHStress_T3     C02 C02_T3
```

Data filtering and normalization

- Remove unreliable features (those never observed in >2 runs per condition)

- Normalization by equalizing the total ion count

```
# remove unreliable features
ftr_rep <- dta_exp %>% group_by(z_iso, seq_mod, cond_id) %>%
    summarise(nb_run = n()) %>%
    summarise(max_rep = max(nb_run)) %>%
    ungroup()

dta_exp <- dta_exp %>% semi_join(filter(ftr_rep, max_rep > 2))

# normalization by equalizing the total ion count
tic_run <- dta_exp %>% group_by(run_id) %>% summarise(tic = sum(auc, na.rm = TRUE))
med_tic <- median(tic_run$tic)

dta_exp <- dta_exp %>% left_join(tic_run) %>%
    mutate(inty = ifelse(auc == 0, NA, auc),
           inty_eqtic = inty * med_tic / tic,
           log2inty = ifelse(inty <= 1, 0, log2(inty)),
           log2inty_eqtic = ifelse(inty_eqtic <= 1, 0, log2(inty_eqtic))) %>%
    select(-tic)

rm(list = c("ftr_rep", "tic_run", "med_tic"))
```

Annotate (un)modified peptides

- Map a peptide to the protein sequence `locate_pep2prot()`
  - protein sequence is obtained from http://www.who.int/medicines/publications/druginformation/innlists/PL108.pdf
- Abbreviate a peptide and its modification forms `abbreviate_mod()`

```
# heavy chain sequence
seq_hc <- paste0("EVQLVESGGGLVQPGGSLRLSCAASGYTFSSYWIEWVRQAPGKGLEWIGE",
                 "ILPGGGDTNYNEIFKGRATFSADTSKNTAYLQMNSLRAEDTAVYYCTRRV",
                 "PIRLDYWGQGTLVTVSSASTKGPSVFPLAPSSKSTSGGTAALGCLVKDYF",
                 "PEPVTVSWNSGALTSGVHTFPAVLQSSGLYSLSSVVTVPSSSLGTQTYIC",
                 "NVNHKPSNTKVDKKVEPKSCDKTHTCPPCPAPELLGGPSVFLFPPKPKDT",
                 "LMISRTPEVTCVVVDVSHEDPEVKFNWYVDGVEVHNAKTKPREEQYNSTY",
                 "RVVSVLTVLHQDWLNGKEYKCKVSNKALPAPIEKTISKAKGQPREPQVYT",
                 "LPPSREEMTKNQVSLTCLVKGFYPSDIAVEWESNGQPENNYKTTPPVLDS",
                 "DGSFFLYSKLTVDKSRWQQGNVFSCSVMHEALHNHYTQKSLSLSPGK")
# light chain sequence
seq_lc <- paste0("DIQLTQSPSSLSASVGDRVTITCKASQSVDYEGDSFLNWYQQKPGKAPKL",
                 "LIYAASNLESGVPSRFSGSGSGTDFTLTISSLQPEDFATYYCQQSNEDPL",
                 "TFGQGTKVEIKRTVAAPSVFIFPPSDEQLKSGTASVVCLLNNFYPREAKV",
                 "QWKVDNALQSGNSQESVTEQDSKDSTYSLSSTLTLSKADYEKHKVYACEV",
                 "THQGLSSPVTKSFNRGEC")

# sequence, position and abbreviation for each modified peptide
df_mod <- dta_exp %>%
    select(seq_pep, seq_mod) %>%
    distinct() %>% rowwise() %>%
    mutate(pos_pep = locate_pep2prot(seq_pep, c(seq_hc, seq_lc), c("hc", "lc")),
           pos_begin = as.integer(unlist(strsplit(pos_pep, "_"))[2]),
           pos_end = as.integer(unlist(strsplit(pos_pep, "_"))[3]),
```

```r
          seq_abbr = abbreviate_mod(seq_mod, pos_begin)) %>%
    ungroup()

head(df_mod) %>% as.data.frame()
```

```
##                  seq_pep                      seq_mod    pos_pep pos_begin
## 1               ALPAPIEK                     ALPAPIEK hc_326_333       326
## 2              PLTFGQGTK                    PLTFGQGTK  lc_98_106        98
## 3                   SCDK              SC[+1315.8]DK hc_218_221       218
## 4    ASQSVDYEGDSFLNWYQQK    ASQSVD[-18]YEGDSFLNWYQQK   lc_24_42        24
## 5 ASQSVDYEGDSFLNWYQQKPGK ASQSVD[-18]YEGDSFLNWYQQKPGK   lc_24_45        24
## 6 ASQSVDYEGDSFLNWYQQKPGK  ASQSVDYEGDSFLN[+1]WYQQKPGK   lc_24_45        24
##   pos_end      seq_abbr
## 1     333         unmod
## 2     106         unmod
## 3     221 C219[+1315.8]
## 4      42       D29[-18]
## 5      45       D29[-18]
## 6      45        N37[+1]
```

Position for each peptide

```r
df_pep <- df_mod %>%
    select(seq_pep, pos_pep, pos_begin, pos_end) %>%
    distinct()

df_pep
```

```
## # A tibble: 42 × 4
##                      seq_pep    pos_pep pos_begin pos_end
##                        <chr>      <chr>     <int>   <int>
## 1                   ALPAPIEK hc_326_333       326     333
## 2                  PLTFGQGTK  lc_98_106        98     106
## 3                       SCDK hc_218_221       218     221
## 4        ASQSVDYEGDSFLNWYQQK   lc_24_42        24      42
## 5     ASQSVDYEGDSFLNWYQQKPGK   lc_24_45        24      45
## 6    DIQLTQSPSSLSASVGDRVTITCK    lc_0_23         0      23
## 7             DSTYSLSSTLTLSK lc_173_186       173     186
## 8        DSTYSLSSTLTLSKADYEK lc_173_191       173     191
## 9  DTLMISRTPEVTCVVVDVSHEDPEVK hc_248_273       248     273
## 10            FNWYVDGVEVHNAK hc_274_287       274     287
## # ... with 32 more rows
```

Site-centered representation `mod2site()`

Extract candidate sites (and their forms) for characterization in consideration of:

- peptides with only one modification sites
- peptides with two modification sites including a carboxymethylated [+58] cysteine

```r
# identify eligible sites
df_site <- mod2site(df_mod) %>%
    mutate(chain = ifelse(grepl("hc", pos_pep), "hc", "lc"),
           site = paste(chain, pos_mod, sep = "_")) %>%
    arrange(site, pos_pep)
```

```r
# compare number of distinct forms & determine the forms to be characterized
soe_cand <- df_site %>% group_by(site, nb_site) %>%
    summarise(nb_form = n_distinct(form)) %>%
    filter(nb_form == max(nb_form)) %>%
    ungroup() %>%
    select(site, nb_site)

# site for the characterization
soe_site <- soe_cand$site
```

Considered sites

```r
soe_site
```

```
##  [1] "hc_21"  "hc_219" "hc_225" "hc_228" "hc_251" "hc_260" "hc_296"
##  [8] "hc_32"  "hc_357" "hc_383" "hc_388" "hc_389" "hc_424" "hc_427"
## [15] "hc_446" "hc_46"  "hc_56"  "hc_58"  "hc_64"  "hc_82"  "hc_95"
## [22] "lc_106" "lc_152" "lc_217" "lc_22"  "lc_23"  "lc_29"  "lc_37"
## [29] "lc_42"  "lc_91"
```

Summarization of feature intensities `quan_mod()`

Three steps are carried out in a sequence:

1. Feature selection `select_ftrs_set()`
2. Imputation of censored missing values
3. Summarization using Tukey's median polish (TMP) method

LogOfSum summarization is also carried out for comparison, where feature selection and missing value imputation are not performed.

```r
dta_eqtic <- dta_exp %>%
    select(seq_pep, seq_mod, z_iso, run_id, log2inty = log2inty_eqtic) %>%
    mutate(pos_pep = plyr::mapvalues(seq_pep, from = df_pep$seq_pep, to = df_pep$pos_pep),
           feature = paste0(pos_pep, "__", z_iso))

df_soesite <- df_site %>% semi_join(soe_cand)
tmpsum <- vector("list", length(soe_site))
lossum <- vector("list", length(soe_site))
for (s in seq_along(soe_site)) {
    onesite <- df_soesite %>% filter(site == soe_site[s])
    ftr_site <- dta_eqtic %>% filter(seq_mod %in% onesite$seq_mod) %>%
        mutate(site = soe_site[s],
               form = plyr::mapvalues(seq_mod, from = onesite$seq_mod, to = onesite$form)) %>%
        select(site, form, run = run_id, feature, log2inty)
    full_site <- ftr_site %>%
        complete(feature, form, run = df_design$run_id) %>%
        mutate(is_obs = !is.na(log2inty))
    ## matrix for feature coverage as input to feature selection
    obs_site <- full_site %>% select(feature, form, run, is_obs) %>%
        spread(feature, is_obs) %>% select(-form, -run) %>% as.matrix()
    idx_ftr <- select_ftrs_set(obs_site, nb_tolmiss = 3)  # index of selected features
    ftrname <- colnames(obs_site)[idx_ftr]
    ## summarization of the abundance of each form
```

```
    tmpsum[[s]] <- quan_mod(ftr_site, ftr_slc = ftrname, meth = "tmp")
    lossum[[s]] <- quan_mod(ftr_site, meth = "los")
}
df_ftrsum <- bind_rows(bind_rows(tmpsum), bind_rows(lossum))

## reference/normalizing peptide
seq_ref <- "VDNALQSGNSQESVTEQDSK"
ftr_site <- dta_eqtic %>% filter(seq_pep == seq_ref) %>%
    mutate(site = "ref", form = "ref") %>%
    select(site, form, run = run_id, feature, log2inty)
df_ftrsum <- bind_rows(df_ftrsum, quan_mod(ftr_site, meth = "tmp"))
df_ftrsum <- bind_rows(df_ftrsum, quan_mod(ftr_site, meth = "los"))

df_ftrsum <- df_ftrsum %>%
    mutate(cond = plyr::mapvalues(run, from = df_design$run_id, to = df_design$cond_id))

rm(list = c("s", "onesite", "ftr_site", "full_site", "obs_site", "idx_ftr", "ftrname"))
```

Data frame of feature intensity and summarization

```
df_ftrsum
```

```
## # A tibble: 73,490 × 8
##      site     form    run log2inty       feature    quan method  cond
##     <chr>    <chr>  <chr>    <dbl>         <chr>   <chr>  <chr> <chr>
## 1   hc_21 C21[+58] C01_T1 27.76646 hc_0_42__z3_0 feature    TMP   C01
## 2   hc_21 C21[+58] C01_T1 29.12803 hc_0_42__z3_1 feature    TMP   C01
## 3   hc_21 C21[+58] C01_T1 29.54923 hc_0_42__z3_2 feature    TMP   C01
## 4   hc_21 C21[+58] C01_T1 29.42852 hc_0_42__z3_3 feature    TMP   C01
## 5   hc_21 C21[+58] C01_T1 28.94854 hc_0_42__z3_4 feature    TMP   C01
## 6   hc_21 C21[+58] C01_T2 27.73985 hc_0_42__z3_0 feature    TMP   C01
## 7   hc_21 C21[+58] C01_T2 29.09808 hc_0_42__z3_1 feature    TMP   C01
## 8   hc_21 C21[+58] C01_T2 29.52376 hc_0_42__z3_2 feature    TMP   C01
## 9   hc_21 C21[+58] C01_T2 29.43070 hc_0_42__z3_3 feature    TMP   C01
## 10  hc_21 C21[+58] C01_T2 28.94316 hc_0_42__z3_4 feature    TMP   C01
## # ... with 73,480 more rows
```

Profile plot of features at one site `plot_pfform()`

```
ss <- "hc_251"
plot_pfform(filter(df_ftrsum, method == "LogOfSum", quan == "feature"), ss)
```

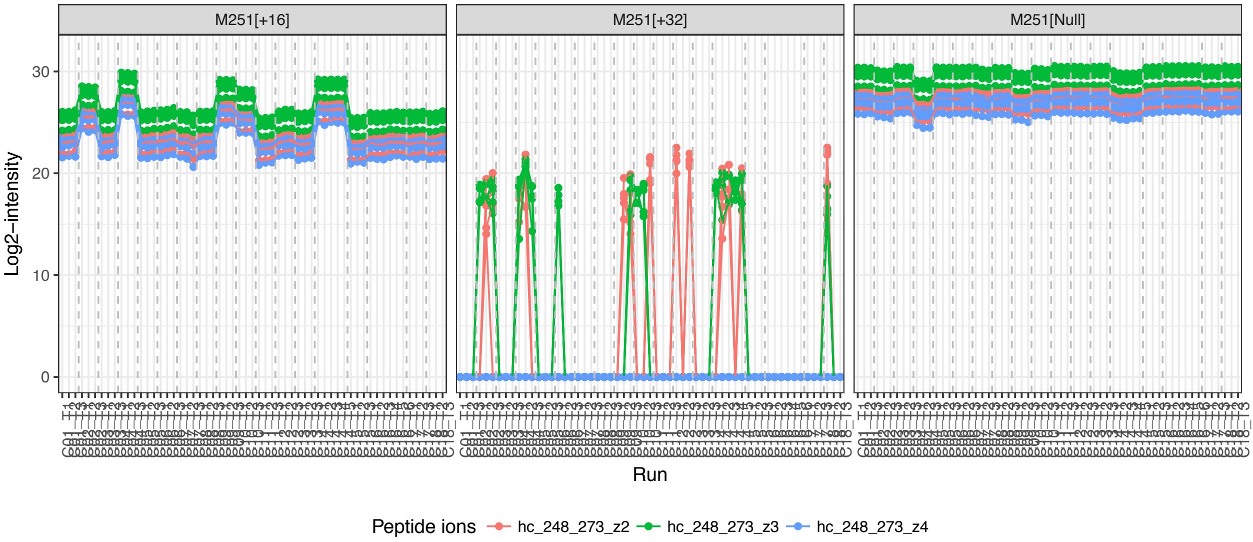

Profile plot of features with summarization `plot_sumform()`

LogOfSum

```
plot_sumform(filter(df_ftrsum, method == "LogOfSum"), ss)
```

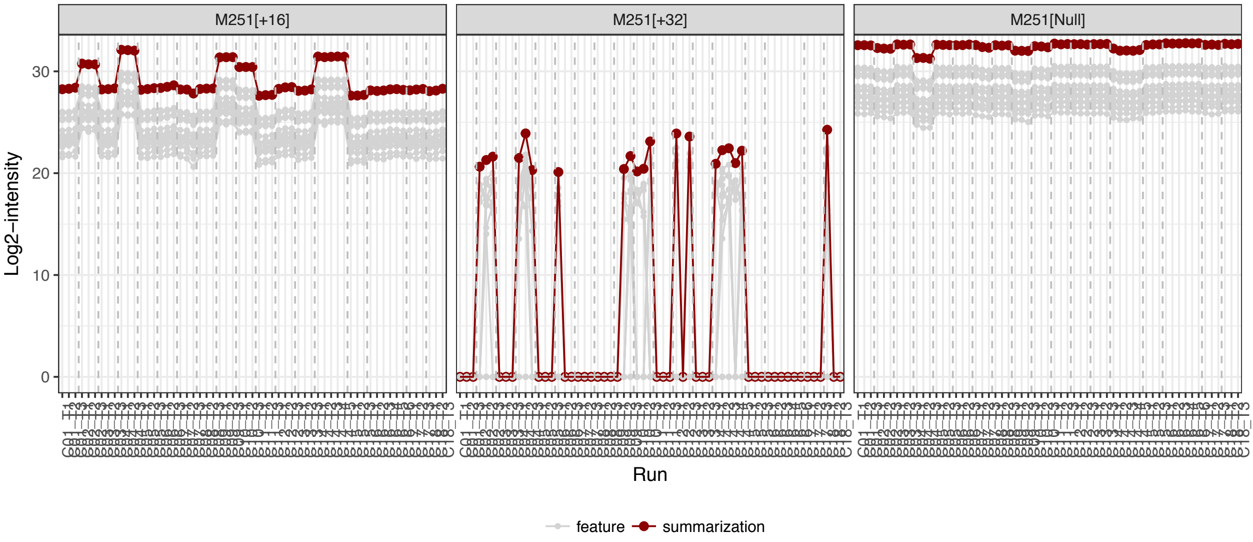

Proposed TMP

```
plot_sumform(filter(df_ftrsum, method == "TMP"), ss)
```

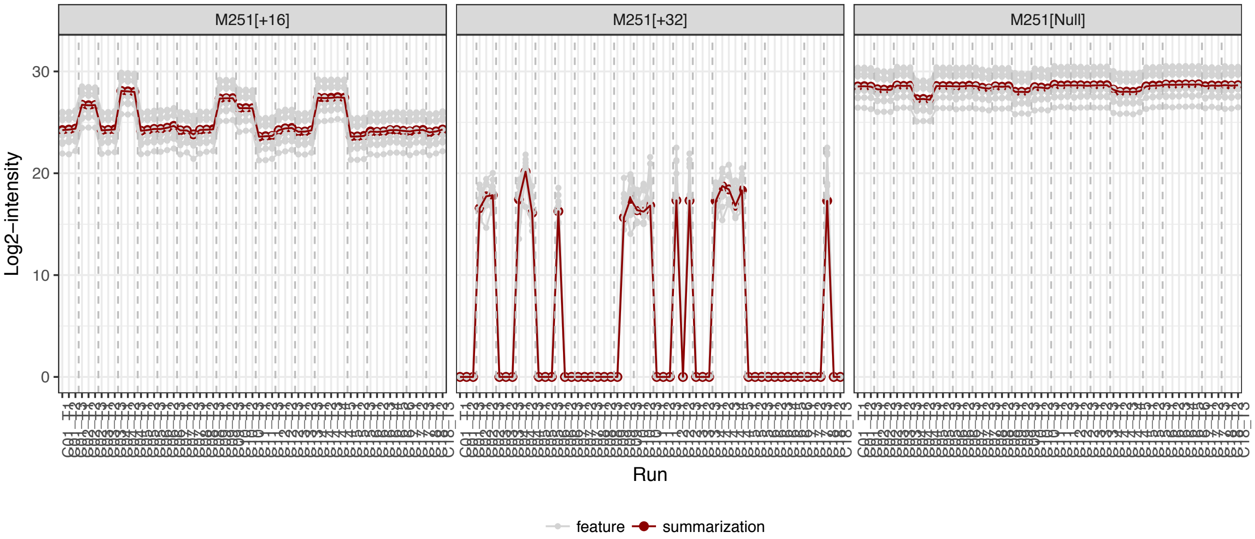

Goal 1: site occupancy estimation

Three methods are considered:

1. Naïve method
2. LogOfSum mehtod
3. Proposed TMP method

Statistical modeling

- Modeling on the scale of log-intensity `model_prop()`
- Modeling on the scale of run-level occupancy `model_occrun()`

```
df_sum <- df_ftrsum %>% filter(quan == "summarization") %>%
    select(site:log2inty, cond, method)
conds <- cond_key$id

# modeling
mdl_tmp <- model_prop(filter(df_sum, method == "TMP"), conds, soe_site, level = "c")
mdl_los <- model_prop(filter(df_sum, method == "LogOfSum"), conds, soe_site, level = "c")
mdl_run <- model_occrun(filter(df_sum, method == "LogOfSum"), conds, soe_site)
```

Inference of site occupancy `est_siteocc()`

```
# site occupancy estimation
soe_tmp <- est_siteocc(mdl_tmp, sumscale = "abundance", conf_level = 0.95) %>%
    mutate(method = "TMP")
soe_los <- est_siteocc(mdl_los, sumscale = "abundance", conf_level = 0.95) %>%
    mutate(method = "LogOfSum")
soe_run <- est_siteocc(mdl_run, sumscale = "occrun", conf_level = 0.95) %>%
    mutate(method = "Naïve")

soe_res <- bind_rows(soe_tmp, soe_los, soe_run)
```

Site occupancy estimates of form M251[+16] with 95% confidence intervals

```
soe_res %>%
    filter(site == "hc_251", cond %in% c("C09", "C10", "C16"), form == "M251[+16]") %>%
    mutate(method = factor(method, levels = c("Naïve", "LogOfSum", "TMP")),
           condition = ifelse(cond == "C16", "AI reference",
                              ifelse(cond == "C10", "AI 3 mM AAPH", "AI 10 mM AAPH"))) %>%
    ggplot(aes(method, occ_est)) +
    geom_pointrange(aes(ymin = occ_lb, ymax = occ_ub), colour = "darkred") +
    xlab("Method") + ylab("Estimated site occupancy (%)") + ggtitle("M251[+16]") +
    facet_wrap(~ condition, nrow = 1) +
    theme_bw() +
    theme(plot.title = element_text(size = 16), strip.text.x = element_text(size = 14))
```

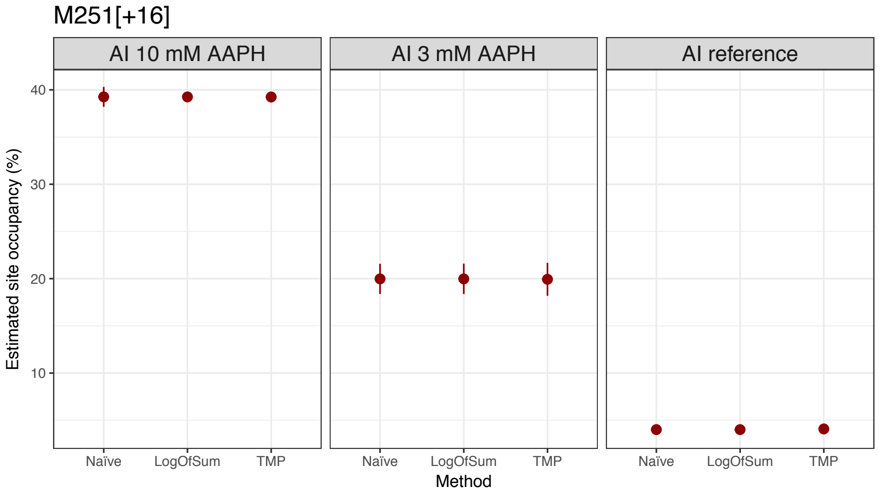

Goal 2: detection of differential site occupancies

Define comparisons:

- ADC reference vs. AI reference
- ADC samples vs. ADC reference
- AI samples vs. AI reference

```
idx_case <- c(which(grepl("ADC-Ref", cond_key$name)),
              which(!grepl("AI-|Ref", cond_key$name)),
              which(!grepl("ADC-|Ref", cond_key$name)))
idx_ctrl <- c(which(grepl("AI-Ref", cond_key$name)),
              rep(which(grepl("ADC-Ref", cond_key$name)), sum(!grepl("AI-|Ref", cond_key$name))),
              rep(which(grepl("AI-Ref", cond_key$name)), sum(!grepl("ADC-|Ref", cond_key$name))))
```

Hypothesis testing based on $H_0^{(1)}$ `test_diffocc()`

```
# differential site occupancy
dso_tmp <- test_diffocc(mdl_tmp, conds, idx_ctrl, idx_case, sumscale = "abundance") %>%
    mutate(method = "TMP")
dso_los <- test_diffocc(mdl_los, conds, idx_ctrl, idx_case, sumscale = "abundance") %>%
    mutate(method = "LogOfSum")
dso_run <- test_diffocc(mdl_run, conds, idx_ctrl, idx_case, sumscale = "occrun") %>%
```

```
    mutate(method = "Naïve")

dso_res <- bind_rows(dso_tmp, dso_los, dso_run)
```

Hypothesis testing based on $H_0^{(2)}$ and $H_0^{(3)}$

Normalization with respect to the reference peptide

```
df_adjsum <- df_sum %>% group_by(method, run) %>%
    mutate(log2inty_ref = log2inty[site == "ref"]) %>%
    ungroup() %>%
    mutate(log2inty = log2inty - log2inty_ref)
```

Statistical modeling on the scale of log-intensity `model_log2inty()`

```
mdl_h2tmp <- model_log2inty(filter(df_sum, method == "TMP"), conds, soe_site)
mdl_h2los <- model_log2inty(filter(df_sum, method == "LogOfSum"), conds, soe_site)
mdl_h3tmp <- model_log2inty(filter(df_adjsum, method == "TMP"), conds, soe_site)
mdl_h3los <- model_log2inty(filter(df_adjsum, method == "LogOfSum"), conds, soe_site)
```

Hypothesis testing for differential abundance `test_diffabun()`

```
dsa2_tmp <- test_diffabun(mdl_h2tmp, conds, idx_ctrl, idx_case) %>%
    mutate(method = "TMP", test = "H2") %>%
    left_join(select(dso_tmp, site, form, control, case, dso_est))
dsa2_los <- test_diffabun(mdl_h2los, conds, idx_ctrl, idx_case) %>%
    mutate(method = "LogOfSum", test = "H2") %>%
    left_join(select(dso_los, site, form, control, case, dso_est))
dsa3_tmp <- test_diffabun(mdl_h3tmp, conds, idx_ctrl, idx_case) %>%
    mutate(method = "TMP", test = "H3") %>%
    left_join(select(dso_tmp, site, form, control, case, dso_est))
dsa3_los <- test_diffabun(mdl_h3los, conds, idx_ctrl, idx_case) %>%
    mutate(method = "LogOfSum", test = "H3") %>%
    left_join(select(dso_los, site, form, control, case, dso_est))
```

Performance evaluation

Evaluation on

- positive control: AI AAPH 10 mM vs. AI reference for sites at Fab, Fc
- negative control: ADC reference vs. AI reference excluding sites for drug modifications

```
# positive control: AI AAPH 10 mM vs AI reference for sites at Fab, Fc
ss_oxi <- c("hc_32", "hc_46", "hc_82", "hc_251", "hc_357", "hc_427")
dso_pos <- dso_res %>% filter(control == "C16", case %in% c("C09", "C10"), site %in% ss_oxi)

# negative control: ADC reference vs AI reference excluding sites for drug modifications
ss_drug <- c("hc_219", "hc_225", "hc_228", "lc_217")
dso_neg <- dso_res %>% filter(control == "C16", case == "C06", !(site %in% ss_drug))
```

Hypothesis testing based on $H_0^{(1)}$

Sensitivity, specificity, and positive predictive value (PPV)

```
# sensitivity of detecting differential site occupancy
eval_pos <- dso_pos %>%
    group_by(method) %>%
    summarise(tp = sum(!is.na(adj_p_val) & adj_p_val < 0.05), nb_pos = n()) %>%
    mutate(fn = nb_pos - tp, sensitivity = round(tp / nb_pos, digits = 3)) %>%
    select(method, tp, fn, nb_pos, sensitivity)

# specificity of detecting differential site occupancy
eval_neg <- dso_neg %>%
    group_by(method) %>%
    summarise(fp = sum(!is.na(adj_p_val) & adj_p_val < 0.05), nb_neg = n()) %>%
    mutate(tn = nb_neg - fp, specificity = round(tn / nb_neg, digits = 3)) %>%
    select(method, fp, tn, nb_neg, specificity)

# PPV
left_join(eval_pos, eval_neg) %>% mutate(ppv = round(tp / (tp + fp), digits = 3)) %>%
    as.data.frame()
```

```
##     method tp fn nb_pos sensitivity fp tn nb_neg specificity   ppv
## 1 LogOfSum 28  6     34       0.824  6 55     61       0.902 0.824
## 2    Naive 28  6     34       0.824 13 49     62       0.790 0.683
## 3      TMP 28  6     34       0.824  5 56     61       0.918 0.848
```

Hypothesis testing based on $H_0^{(2)}$ and $H_0^{(3)}$

```
dsa_res <- bind_rows(dsa2_tmp, dsa2_los, dsa3_tmp, dsa3_los)
dsa_pos <- dsa_res %>% filter(control == "C16", case %in% c("C09", "C10"), site %in% ss_oxi)
dsa_neg <- dsa_res %>% filter(control == "C16", case == "C06", !(site %in% ss_drug))

eval_posa <- dsa_pos %>%
    group_by(test, method) %>%
    summarise(tp = sum(!is.na(adj_p_val) & adj_p_val < 0.05), nb_pos = n()) %>%
    ungroup() %>%
    mutate(fn = nb_pos - tp, sensitivity = round(tp / nb_pos, digits = 3)) %>%
    select(test, method, tp, fn, nb_pos, sensitivity)

eval_nega <- dsa_neg %>%
    group_by(test, method) %>%
    summarise(fp = sum(!is.na(adj_p_val) & adj_p_val < 0.05), nb_neg = n()) %>%
    ungroup() %>%
    mutate(tn = nb_neg - fp, specificity = round(tn / nb_neg, digits = 3)) %>%
    select(test, method, fp, tn, nb_neg, specificity)

left_join(eval_posa, eval_nega) %>% mutate(ppv = round(tp / (tp + fp), digits = 3)) %>%
    as.data.frame()
```

```
##   test   method tp fn nb_pos sensitivity fp tn nb_neg specificity   ppv
## 1   H2 LogOfSum 22 12     34       0.647 10 51     61       0.836 0.688
## 2   H2      TMP 23 11     34       0.676  9 52     61       0.852 0.719
## 3   H3 LogOfSum 21 13     34       0.618 12 49     61       0.803 0.636
## 4   H3      TMP 22 12     34       0.647 13 48     61       0.787 0.629
```

Volcano plots `plot_volcano()`

Positive control based $H_0^{(1)}$

```r
plot_volcano(filter(dso_pos, method == "TMP"), plim = 10, dlim = 40,
             gname = "Positive control, proposed method")
```

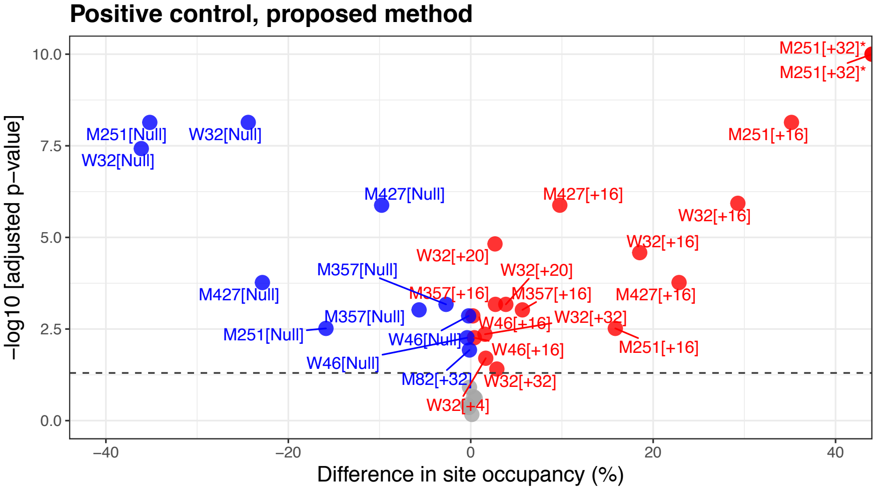

Positive control based $H_0^{(2)}$

```r
plot_volcano(filter(dsa_pos, method == "TMP", test == "H2"), plim = 10, dlim = 40,
             gname = "Positive control, proposed method")
```

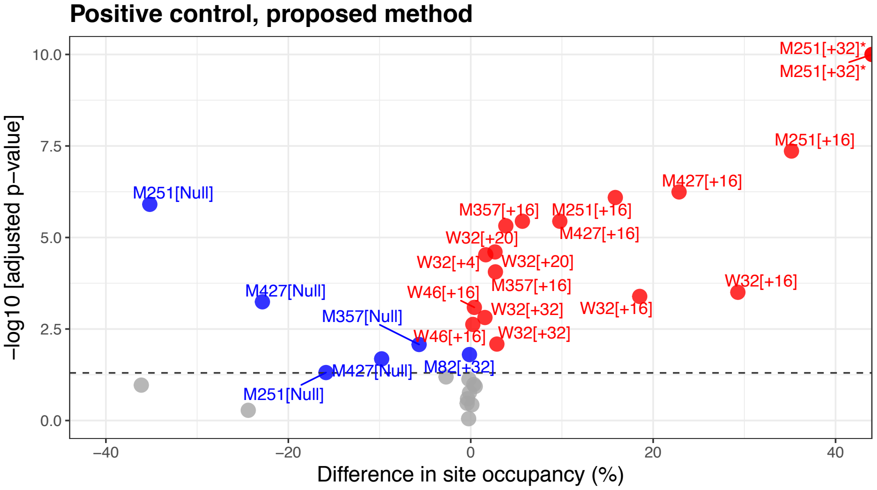

Positive control based $H_0^{(3)}$

```r
plot_volcano(filter(dsa_pos, method == "TMP", test == "H3"), plim = 10, dlim = 40,
             gname = "Positive control, proposed method")
```

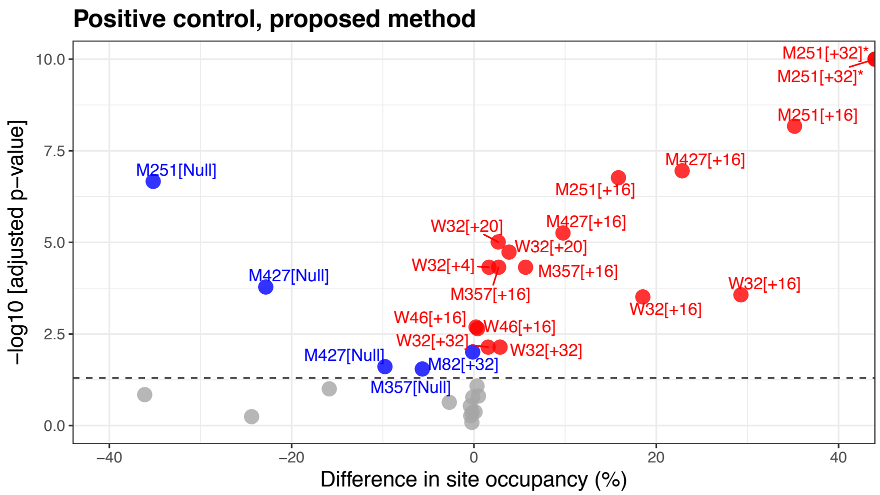

Negative control based $H_0^{(1)}$

```
plot_volcano(filter(dso_neg, method == "TMP"), plim = 10, dlim = 15,
             gname = "Negative control, proposed method")
```

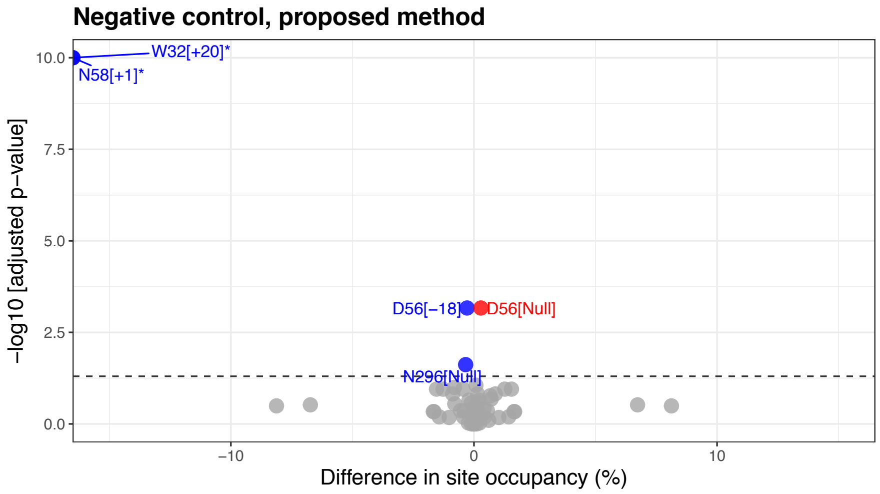

Negative control based $H_0^{(2)}$

```
plot_volcano(filter(dsa_neg, method == "TMP", test == "H2"), plim = 10, dlim = 15,
             gname = "Negative control, proposed method")
```

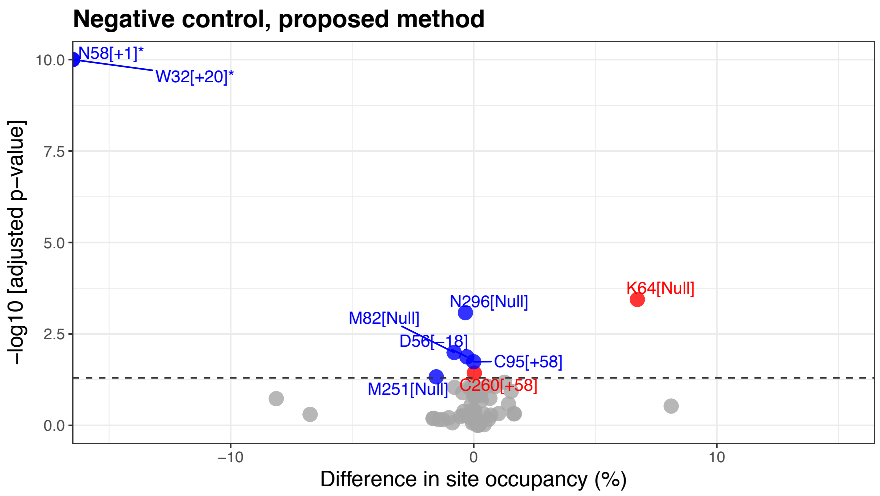

Negative control based $H_0^{(3)}$

```
plot_volcano(filter(dsa_neg, method == "TMP", test == "H3"), plim = 10, dlim = 15,
             gname = "Negative control, proposed method")
```

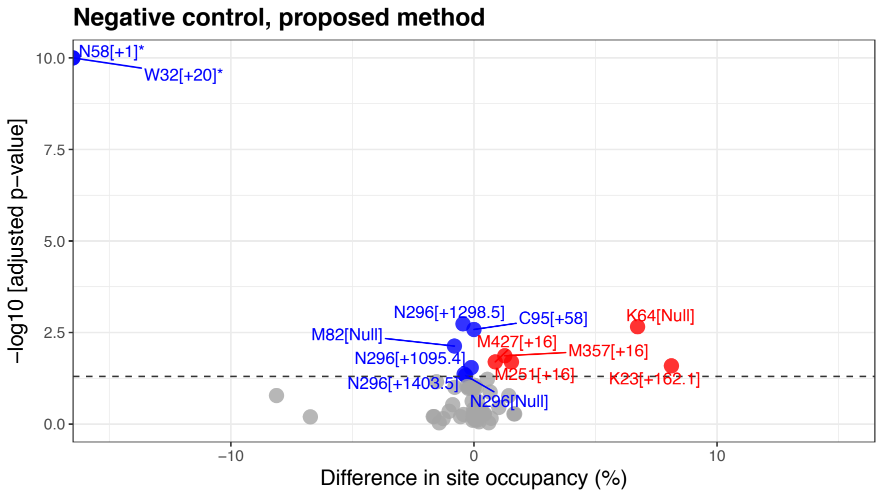

Goal 3: combined occupancy estimation

Define forms and sites to be combined - oxidative forms ([+4], [+16], [+20], [+32]):

- At each site in Fab (hc_32, hc_46, hc_82) and Fc (hc_251, hc_357, hc_427)
- Across sites in Fab and Fc

```
# combined occupancy estimation
oxilist <- list(Fab = c("hc_32", "hc_46", "hc_82"), Fc = c("hc_251", "hc_357", "hc_427"),
                hc_32 = "hc_32", hc_46 = "hc_46", hc_82 = "hc_82",
                hc_251 = "hc_251", hc_357 = "hc_357", hc_427 = "hc_427")

combform <- list(Null = c("[+58]", "[Null]"), Oxidation = c("[+4]", "[+16]", "[+20]", "[+32]"))
ptnform <- vector("character", length(combform))
for (i in seq_along(ptnform)) {
    tmp <- paste(combform[[i]], collapse = "|")
    tmp <- gsub("\\[", "\\\\[", tmp)
```

```
    tmp <- gsub("\\]", "\\\\]", tmp)
    tmp <- gsub("\\+", "\\\\+", tmp)
    ptnform[i] <- tmp
}
```

Estimate combined occupancy `est_combocc()`

```
coe_tmp <- filter(df_sum, method == "TMP") %>%
    est_combocc(conds, oxilist, ptnform, names(combform), sumscale = "abundance") %>%
    mutate(method = "TMP")
coe_los <- filter(df_sum, method == "LogOfSum") %>%
    est_combocc(conds, oxilist, ptnform, names(combform), sumscale = "abundance") %>%
    mutate(method = "LogOfSum")
coe_run <- filter(df_sum, method == "LogOfSum") %>%
    est_combocc(conds, oxilist, ptnform, names(combform), sumscale = "occrun") %>%
    mutate(method = "Naïve")

coe_res <- bind_rows(coe_tmp, coe_los, coe_run)
```

Estimation results

```
coe_site <- coe_res %>%
    filter(combsite %in% ss_oxi) %>%
    mutate(frag = ifelse(combsite %in% oxilist$Fab, "Fab", "Fc"),
           aapos = plyr::mapvalues(combsite,
                                   from = c("hc_32", "hc_46", "hc_82", "hc_251", "hc_357", "hc_427"),
                                   to = c("W32", "W46", "M82", "M251", "M357", "M427")))
coe_site$method <- factor(coe_site$method, levels = c("Naïve", "LogOfSum", "TMP"))
coe_site$aapos <- factor(coe_site$aapos, levels = c("W32", "W46", "M82", "M251", "M357", "M427"))

# compare with orthogonal data
coe_frag <- coe_res %>%
    filter(combsite %in% c("hc_32-hc_46-hc_82", "hc_251-hc_357-hc_427")) %>%
    mutate(frag = ifelse(combsite == "hc_32-hc_46-hc_82", "Fab", "Fc"),
           method = factor(method, levels = c("Naïve", "LogOfSum", "TMP"))) %>%
    mutate(occ_orth = ifelse(frag == "Fab", 1.57, 3.88))
```

```
coe_site %>%
    filter(cond == "C16", mod == "Oxidation") %>%
    ggplot(aes(method, occ_est, colour = aapos, group = aapos)) +
    geom_pointrange(aes(ymin = occ_lb, ymax = occ_ub), position = position_dodge(width = 0.25)) +
    scale_x_discrete(breaks = c("Naïve", "LogOfSum", "TMP"),
                     labels = c("Naïve", "LogOfSum", "Proposed\n(TMP)")) +
    xlab("Method") + ylab("Estimated combined occupancy (%)") +
    ggtitle("Combined occupancy at each site") +
    coord_cartesian(ylim = c(0, 4.5)) +
    facet_wrap(~ frag, nrow = 1) +
    theme_bw() +
    scale_colour_discrete(name = "Site") +
    theme(legend.position = "bottom", legend.box = "horizontal", legend.direction="horizontal") +
    guides(colour = guide_legend(nrow = 1)) +
```

```r
    theme(axis.text = element_text(size = 12),
          axis.title = element_text(size = 14),
          plot.title = element_text(size = 16, face = "bold"),
          strip.text.x = element_text(size = 14))
```

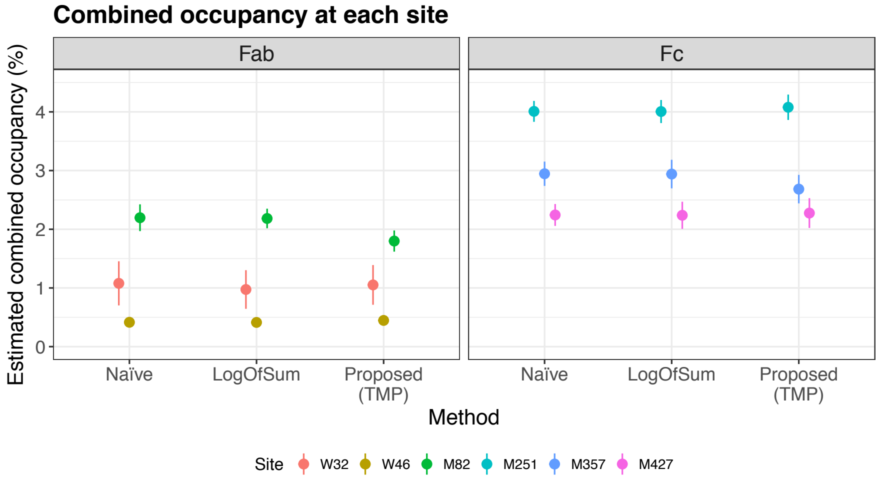


```r
coe_frag %>% filter(cond == "C16", mod == "Oxidation") %>%
    ggplot(aes(method, occ_est)) +
    geom_pointrange(aes(ymin = occ_lb, ymax = occ_ub), colour = "darkred") +
    geom_hline(aes(yintercept = occ_orth), linetype = 2) +
    scale_x_discrete(breaks = c("Naïve", "LogOfSum", "TMP"),
                     labels = c("Naïve", "LogOfSum", "Proposed\n(TMP)")) +
    xlab("Method") + ylab("Estimated combined occupancy (%)") +
    ggtitle("Combined occupancy in Fab and Fc") +
    coord_cartesian(ylim = c(0, 4.5)) +
    facet_wrap(~ frag, nrow = 1) +
    theme_bw() +
    theme(axis.text = element_text(size = 12),
          axis.title = element_text(size = 14),
          plot.title = element_text(size = 16, face = "bold"),
          strip.text.x = element_text(size = 14))
```

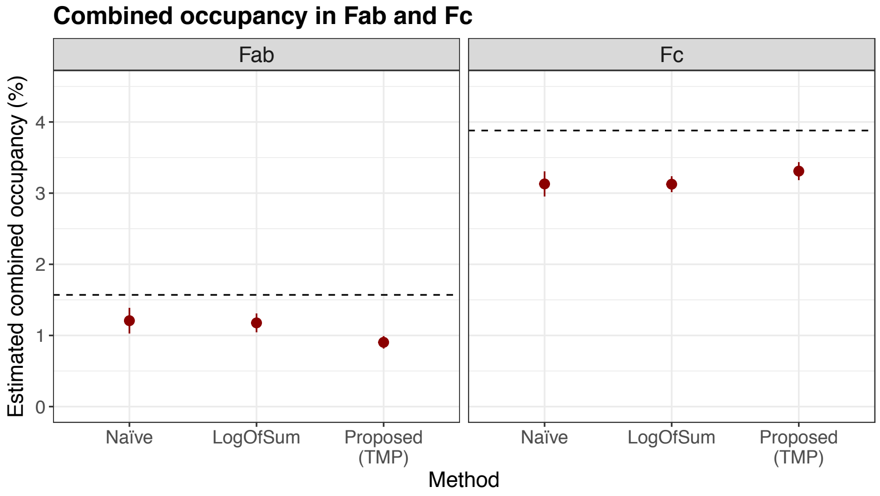

Code in `protchar-methods.R`

```
## Data manipulations ----------------------------------------------
## Locate and rename unmodified peptide (position index starts at 0)
## INPUT:
##   -- seq_pep: peptide sequence
##   -- seq_prot: protein sequence
##   -- prot_abbr: abbreviation of protein name
## OUTPUT:
##   -- renamed peptide as "ProteinAbbr_PosStart_PosEnd"
# locate_pep2ptn
locate_pep2prot <- function(seq_pep, seq_prot, prot_abbr) {
    if (length(seq_prot) != length(prot_abbr)) {
        stop("Numbers of proteins and protein abbreviations should be identical!")
    }
    pos_char <- NA
    for (p in 1:length(seq_prot)) {
        if (grepl(seq_pep, seq_prot[p])) {
            idx_seq <- regexpr(seq_pep, seq_prot[p])
            pos_begin <- idx_seq - 1  # position starting at 0
            pos_end <- nchar(seq_pep) + pos_begin - 1
            pos_char <- paste(prot_abbr[p], pos_begin, pos_end, sep = "_")
        }
    }

    return(pos_char)
}


## Abbreviate a modified peptide with its site (AA & position) and mass shift
## INPUT:
##   -- seq_mod: sequence of the modified peptide
##   -- pos_start: starting AA position of peptide
## OUTPUT:
##   -- seq_abbr: abbreviated peptide name as "AAPos[MassShift]" or "unmod"
abbreviate_mod <- function(seq_mod, pos_start) {
    if (pos_start < 0) stop("The smallest possible AA index is '0'")
    ## indices of square brackets
    idx_brkt <- unlist(gregexpr(pattern = "\\[|\\]", seq_mod))  # return -1 if no match
    len_idx <- length(idx_brkt)
    if (len_idx == 1) {
        seq_abbr <- "unmod"
    } else {
        idx_lbrkt <- idx_brkt[seq(from = 1, to = length(idx_brkt) - 1, by = 2)]
        idx_rbrkt <- idx_brkt[seq(from = 2, to = length(idx_brkt), by = 2)]
        aa <- substring(seq_mod, idx_lbrkt - 1, idx_lbrkt - 1)  # amino acid
        ## number of characters for each bracket used in allocating AA positions
        nb_char <- idx_rbrkt - idx_lbrkt + 1
        pos_adj <- if (len_idx == 2) 0 else cumsum(c(0, nb_char[1:(len_idx / 2 - 1)]))
        aa_pos <- pos_start + idx_lbrkt - 2 - pos_adj
        mshift <- substring(seq_mod, idx_lbrkt, idx_rbrkt)  # mass shift
        seq_abbr <- paste0(aa, aa_pos, mshift, collapse = "")
    }
```

```
    return(seq_abbr)
}


## Convert peptide-centered representation to site-centered representation
## INPUT:
##   -- df_mod: data frame for peptide & modifications with the following fields:
##     $ seq_pep: peptide sequence
##     $ pos_pep: peptide represented by protein name and its AA positions: ProteinAbbr_PosStart_PosEnd
##     $ pos_begin: starting AA position of peptide
##     $ pos_end: ending AA position of peptide
##     $ seq_mod: modified peptide sequence
##     $ seq_abbr: abbreviated name of modified peptide (AAPos[MassShift])
## OUTPUT:
##   -- df_site: data frame for site-centered representation with fields:
##     $ seq_pep: peptide sequence
##     $ pos_pep: position of peptide defined by the beginning/ending its AA positions
##     $ seq_mod: modified peptide sequence
##     $ seq_abbr: abbreviated name of modified peptide (AAPos[MassShift])
##     $ aapos: represented by AA residue and its position (AAPos)
##     $ form: represented by AAPos[MassShift] or AAPos[Null]
##     $ nb_site: number of modification sites for the peptide
##     $ pos_mod: position of the site
## NB:
##   -- only peptides with one modification site are considered with exception
##      to have another carboxymethylated [+58] cysteine
##   -- may be extended to cases with more sites if necessary
##   -- matching through peptide sequence, may be extended to consider a case
##      with fully-cleaved modified peptide and partially-cleaved unmodified peptide
mod2site <- function(df_mod) {
    ## housekeeping
    if (!is.data.frame(df_mod)) stop("'df_mod' must be a data.frame")
    if (is.null(df_mod$seq_pep)) stop("column 'seq_pep' missing")
    if (is.null(df_mod$pos_pep)) stop("column 'pos_pep' missing")
    if (is.null(df_mod$seq_mod)) stop("column 'seq_mod' missing")
    if (is.null(df_mod$seq_abbr)) stop("column 'seq_abbr' missing")
    df_site <- NULL  # info about modification sites to be used (to be concatenated)
    df_cys2 <- NULL  # as df_site, but for those with 2 sites including a cysteine
    df_wmod <- df_mod %>% filter(seq_abbr != "unmod")  # modified peptides
    for (i in 1:nrow(df_wmod)) {
        abbr <- df_wmod$seq_abbr[i]
        idx <- unlist(gregexpr(pattern = "\\[|\\]", abbr))  # indices of (l/r) brackets
        len_idx <- length(idx)  # length of the index vector
        nb_msite <- len_idx / 2  # number of bracket pairs (modification sites)
        idxl <- idx[seq(from = 1, to = len_idx - 1, by = 2)]  # left index
        idxr <- idx[seq(from = 2, to = len_idx, by = 2)]  # right index
        mshift <- substring(abbr, idxl, idxr)  # extract mass shift
        msite <- unlist(strsplit(abbr, "\\[[+-]\\d+(\\.\\d+)?\\]"))  # extract sites
        ## NB: modsite defined below may have >1 rows (one site per row)
        modsite <- data_frame(seq_pep = df_wmod$seq_pep[i],
                              pos_pep = df_wmod$pos_pep[i],
                              seq_mod = df_wmod$seq_mod[i],
                              seq_abbr = abbr, aapos = msite,
```

```
                             form = paste0(msite, mshift), nb_site = nb_msite) %>%
            mutate(pos_mod = as.integer(gsub("[^0-9]", "", aapos)))
        if (nb_msite == 1) {
            ## only one modification site
            df_site <- bind_rows(df_site, modsite)
        } else if (nb_msite == 2) {
            ## only sites accompancying another [+58] are relevant
            is_cys <- (mshift == "[+58]")
            if (any(is_cys)) {
                ## to be used for matching the [+58] site
                df_cys2 <- bind_rows(df_cys2, modsite)
                if (sum(is_cys) == 1) {
                    ## one carboxymethylated cysteine residue
                    df_site <- bind_rows(df_site, modsite[which(!is_cys), ])
                } else {
                    ## two carboxymethylated cysteine residues
                    df_site <- bind_rows(df_site, modsite)
                }
            }
        }
    }
    ## matching sites with unmodified forms
    df_womod <- df_mod %>% filter(seq_abbr == "unmod")
    df_site1 <- df_site %>% filter(nb_site == 1)
    for (pp in unique(df_site1$pos_pep)) {
        if (pp %in% df_womod$pos_pep) {
            modsite <- df_site1 %>%
                filter(pos_pep == pp) %>%
                select(seq_pep, aapos, pos_mod) %>% distinct() %>%
                mutate(pos_pep = pp, seq_mod = seq_pep, seq_abbr = "unmod",
                       form = paste0(aapos, "[Null]"), nb_site = 1)
            df_site <- bind_rows(df_site, modsite)
        }
    }
    ## matching sites with unmodified forms accompanying [+58]
    df_cys1 <- df_site1 %>% filter(grepl("\\[\\+58\\]", form))
    for (i in 1:nrow(df_cys1)) {
        pp <- df_cys1$pos_pep[i]
        ss <- df_cys1$aapos[i]
        if (pp %in% df_cys2$pos_pep) {
            tmp_match <- df_cys2 %>% filter(pos_pep == pp) %>%
                mutate(is_match = (aapos == ss)) %>%
                group_by(seq_abbr) %>% filter(sum(is_match) == 1) %>% ungroup() %>%
                filter(!is_match) %>% distinct(aapos, pos_mod)
            if (nrow(tmp_match) > 0) {
                modsite <- data_frame(seq_pep = df_cys1$seq_pep[i], pos_pep = pp,
                                      seq_mod = df_cys1$seq_mod[i], seq_abbr = df_cys1$seq_abbr[i],
                                      aapos = tmp_match$aapos, form = paste0(tmp_match$aapos, "[Null]")
                                      pos_mod = tmp_match$pos_mod, nb_site = 2)
                df_site <- bind_rows(df_site, modsite)
            }
        }
    }
```

```r
    return(df_site)
}


## Summarization of feature intensities -----------------------------
## Feature selection as a weighted set cover problem, solved by a greedy algorithm
## INPUT:
##   -- mx_obs: data matrix with elements of TRUE (observed) and FALSE (unobserved),
##      where form-run pairs are in rows and features are in columns
##   -- nb_tolmiss: tolerance of missing values (default 0) for the cost function
## OUTPUT:
##   -- set_ftr: a selected set of feature indices
## NB:
##   -- cost function for each feature defined as the number of missing values (runs)
select_ftrs_set <- function(mx_obs, nb_tolmiss) {
    if (ncol(mx_obs) == 1) return(1)  # only one feature
    if (is.null(nb_tolmiss)) nb_tolmiss <- 0
    if (nb_tolmiss < 0)
        stop("'nb_tolmiss' cannot be a negative value")
    nb_obsftr <- rowSums(mx_obs)  # number of observed features in each form-run pair
    mx_wobs <- mx_obs[nb_obsftr != 0, ]  # remove uncovered pair
    cost_ftr <- colSums(!mx_wobs)  # number of missing values as cost
    cost_ftr <- (cost_ftr > nb_tolmiss) * cost_ftr  # allow few missing values
    ## return all fully covering features
    if (any(cost_ftr == 0)) return(which(cost_ftr == 0))
    is_complete <- FALSE
    set_ftr <- NULL
    ## find features with the smallest cost (# of missing / new additions)
    ## until no more additions can be found (all pairs are covered)
    ## NB: pairs covered by selected features will be removed from mx_wobs
    while (!is_complete) {
        if (nrow(mx_wobs) == 1) {
            add <- c(mx_wobs)
        } else {
            add <- colSums(mx_wobs)
        }
        if (all(add == 0)) {
            is_complete <- TRUE
        } else {
            cost_per_add <- cost_ftr / add
            min_cost <- min(cost_per_add)
            new_ftr <- which(cost_per_add == min_cost)
            set_ftr <- c(set_ftr, new_ftr)
            if (length(new_ftr) == 1) {
                idx_add <- which(mx_wobs[, new_ftr])
                # idx_add <- which(mx_wobs[, new_ftr] != 0)
            } else {
                idx_add <- which(rowSums(mx_wobs[, new_ftr, drop = FALSE]) != 0)
            }
            mx_wobs[, new_ftr] <- FALSE  # exclude selected features from further consideration
            mx_wobs <- mx_wobs[-idx_add, , drop = FALSE]  # remove covered pairs
            if (nrow(mx_wobs) == 0) is_complete <- TRUE
        }
```

```
    }

    return(set_ftr)
}


## quantification of modified forms using log of sum
## function quan_mod_los is called by function quan_mod
## it does not apply any feature selection or imputation
quan_mod_los <- function(dta_modftr) {
    dta_ftr_los <- dta_modftr %>% mutate(quan = "feature", method = "LogOfSum") %>%
        select(site, form, run, log2inty, feature, quan, method)
    dta_sum_los <- dta_ftr_los %>% group_by(site, form, run) %>%
        summarise(log2inty = log2(sum(2 ^ log2inty, na.rm = TRUE))) %>%
        ungroup() %>% mutate(feature = "los", quan = "summarization", method = "LogOfSum")
    dta_ftrsum <- bind_rows(dta_ftr_los, dta_sum_los)

    return(dta_ftrsum)
}


## quantification of modification forms at one site
## dta_modftr: data frame with the following fields:
##   -- site, form, run, log2inty, feature
##   -- !!probably don't need column 'site' unless doing error correction!!
## ftr_slc: selected features
quan_mod <- function(dta_modftr, ftr_slc = NULL, meth = "tmp") {
    ## housekeeping
    if (!is.data.frame(dta_modftr))
        stop("'dta_modftr' must be a data.frame")
    if (is.null(dta_modftr$site))
        stop("column 'site' missing")
    if (is.null(dta_modftr$form))
        stop("column 'form' missing")
    if (is.null(dta_modftr$run))
        stop("column 'run' missing")
    if (is.null(dta_modftr$log2inty))
        stop("column 'log2inty' missing")
    if (is.null(dta_modftr$feature))
        stop("column 'feature' missing")
    if (is.null(ftr_slc))
        ftr_slc <- unique(dta_modftr$feature)
    dta_modftr$run <- as.character(dta_modftr$run)
    ## summaization using log of sum
    if (meth == "los")
        return(quan_mod_los(dta_modftr))
    ftr_obs <- dta_modftr %>% filter(feature %in% ftr_slc) %>% group_by(feature) %>%
        summarise(nb_form = n_distinct(form), nb_run = n()) %>%
        mutate(nb_maxform = max(nb_form), quan = (nb_form == nb_maxform))
    ftr_quan <- ftr_obs$feature[ftr_obs$quan]
    ## exclude runs without any measurement in a form from imputation
    dta_modf2 <- dta_modftr %>% filter(feature %in% ftr_slc) %>%
        complete(form, run, feature) %>% group_by(form, run) %>%
```

```
        mutate(nb_obs = sum(!is.na(log2inty))) %>% ungroup() %>% filter(nb_obs != 0) %>%
        mutate(cen = ifelse(is.na(log2inty) & feature %in% ftr_quan, 0, 1))
    tmp_abbr <- unique(dta_modf2$form)  # forms of the peptide
    dta_modf_imp <- NULL
    dta_modf_bfi <- NULL
    form_bfi <- NULL
    for (ff in tmp_abbr) {
        dta_aft <- dta_modf2 %>% filter(form == ff, cen == 0 | !is.na(log2inty)) %>%
            group_by(feature, form) %>% mutate(log2inty_min = min(log2inty, na.rm = TRUE)) %>% ungroup(
            mutate(log2intyy = ifelse(cen == 1, log2inty, 0.99 * log2inty_min))
        obs4quan <- dta_aft %>% filter(feature %in% ftr_quan) %>%
            group_by(feature) %>% summarise(nb_obs = sum(!is.na(log2inty)))
        if (any(obs4quan$nb_obs == 0)) {
            form_bfi <- c(form_bfi, ff)  # require between-form imputation
        } else {
            ## impute censored values using accelerated failure time model
            if (length(unique(dta_aft$feature)) == 1) {
                mdl <- survreg(Surv(log2intyy, cen, type = "left") ~ run, data = dta_aft, dist = "gauss
            } else {
                mdl <- survreg(Surv(log2intyy, cen, type = "left") ~ run + feature, data = dta_aft, dis
            }
            dta_postaft <- dta_aft %>%
                mutate(log2inty_pred = predict(mdl, newdata = dta_aft, type = "response"),
                       log2inty = ifelse(cen == 0, log2inty_pred, log2inty)) %>%
                select(feature, form, run, log2inty)
            dta_modf_imp <- bind_rows(dta_modf_imp, dta_postaft)
            ## data for use in between-form imputation
            if (any(!(unique(dta_postaft$feature) %in% ftr_quan))) {
                dta_modf_bfi <- dta_postaft %>% filter(feature %in% ftr_quan) %>%
                    select(run, feature, form, log2inty) %>%
                    bind_rows(dta_modf_bfi)
                dta_modf_bfi <- dta_postaft %>% filter(!(feature %in% ftr_quan)) %>%
                    group_by(run) %>% summarise(log2inty = median(log2inty, na.rm = TRUE)) %>%
                    mutate(form = ff, feature = "ftr_med") %>%
                    bind_rows(dta_modf_bfi)
            }
        }
    }
    if (!is.null(form_bfi)) {
        dta_modf_bfi <- dta_modf_bfi %>% complete(run, form, feature)
        for (ff in form_bfi) {
            dta_med <- dta_modf2 %>%
                filter(form == ff, !(feature %in% ftr_quan), !is.na(log2inty)) %>%
                group_by(run) %>% summarise(ftr_med = median(log2inty, na.rm = TRUE))
            for (pf in ftr_quan) {
                dta_bfi <- dta_modf_bfi %>% filter(feature %in% c("ftr_med", pf)) %>%
                    group_by(run, form) %>%
                    summarise(diff_form = diff(log2inty)) %>%
                    summarise(diff_bfi = median(diff_form, na.rm = TRUE))
                dta_postbfi <- dta_bfi %>% right_join(dta_med) %>%
                    mutate(feature = pf, form = ff, log2inty = ftr_med + diff_bfi) %>%
                    select(feature, form, run, log2inty)
                dta_modf_imp <- bind_rows(dta_modf_imp, dta_postbfi)
```

```
            }
        }
    }
    dta_ftrsum <- NULL
    for (ff in tmp_abbr) {
        dta_modff <- dta_modf_imp %>% filter(feature %in% ftr_quan, form == ff) %>%
            mutate(site = dta_modftr$site[1], quan = "feature", method = "TMP") %>%
            select(site, form, run, log2inty, feature, quan, method)
        dta_ftrsum <- bind_rows(dta_ftrsum, dta_modff)
        ## TMP summarization
        ttmp <- dta_modff %>% filter(feature %in% ftr_quan) %>%
            select(feature, run, log2inty) %>%
            spread(feature, log2inty)
        dt_tmp <- data.matrix(ttmp[, -1])
        dt_tmp[is.na(dt_tmp)] <- 0
        mp_tmp <- medpolish(dt_tmp, na.rm = TRUE, trace.iter = FALSE)
        ssum_df <- data_frame(site = dta_modftr$site[1], form = ff, run = ttmp$run,
                              log2inty = mp_tmp$overall + mp_tmp$row)
        ## replace "0" by NA (this should be handled from upstream)
        ssum_df$log2inty[ssum_df$log2inty == 0] <- NA
        dta_ftrsum <- bind_rows(dta_ftrsum, mutate(ssum_df, feature = "tmp", quan = "summarization", me
    }

    return(dta_ftrsum)
}


## modeling, occupancy estimation, differential site occupancy ------
## modeling based on proportion of abundance
model_prop <- function(dta_summod, conditions, sites, level = "c") {
    ## level: "c" (condition), "fc" (form across conditions), "sf" (site across forms)
    dta_summod <- dta_summod %>% filter(site %in% sites, cond %in% conditions)
    nb_rep <- dta_summod %>% group_by(site, cond, form) %>%
        summarise(nb_run = n_distinct(run)) %>% ungroup()
    ## fit linear models and combine the results
    if (level == "c") {
        ## linear model for each form in each condition
        df_form <- dta_summod %>%
            group_by(site, form, cond) %>%
            do(tidy(lm(log2inty ~ 1, data = .))) %>%
            ungroup() %>%
            select(site, cond, form, log2abun_est = estimate, log2abun_se = std.error)
    } else if (level == "fc") {
        ## linear model for each form across conditions
        df_form <- dta_summod %>%
            group_by(site, form) %>%
            do(tidy(lm(log2inty ~ cond - 1, data = .))) %>%
            ungroup() %>%
            mutate(cond = gsub("cond", "", term)) %>%
            select(site, cond, form, log2abun_est = estimate, log2abun_se = std.error)
    }
    ## remove singletons (that inhibit inference)
    df_form <- df_form %>%
```

```
        mutate(abun = 2 ^ log2abun_est) %>%
        inner_join(nb_rep) %>%
        filter(nb_run > 1)
    lmres <- matrix(vector("list", length(sites) * length(conditions)), nrow = length(sites))
    for (i in seq_along(sites)) {
        onesite <- df_form %>% filter(site == sites[i])
        for (j in seq_along(conditions)) {
            onecond <- onesite %>% filter(cond == conditions[j]) %>%
                mutate(abun_sum = sum(abun), abun_rel = abun / abun_sum, abun_relc = 1 - abun_rel)
            ## gradient of site occupancy in the following 3 lines
            occ_grad <- -crossprod(t(onecond$abun_rel))
            diag(occ_grad) <- onecond$abun_rel * onecond$abun_relc
            occ_grad <- log(2) * occ_grad
            ## calculate the SE and DF using the Delta method
            lmres[[i, j]] <- onecond %>%
                mutate(abun_rel_se = as.vector(sqrt(occ_grad ^ 2 %*% log2abun_se ^ 2)),
                       df_num = abun_rel_se ^ 4,
                       df_den = as.vector(occ_grad ^ 4 %*% (log2abun_se ^ 4 / (nb_run - 1)))) %>%
                select(site, cond, form, nb_run, abun, abun_rel, abun_rel_se, df_num, df_den)
        }
    }
    rownames(lmres) <- sites
    colnames(lmres) <- conditions

    return(lmres)
}


## modeling on the scale of run-level site occupancy
model_occrun <- function(dta_summod, conditions, sites) {
    dta_summod <- dta_summod %>% filter(site %in% sites, cond %in% conditions)
    nb_rep <- dta_summod %>% group_by(site, cond) %>%
        summarise(nb_run = n_distinct(run)) %>% ungroup()
    ## remove singletons (that inhibit inference)
    dta_summod <- dta_summod %>%
        mutate(inty_form = 2 ^ log2inty) %>%
        inner_join(nb_rep) %>%
        filter(nb_run > 1)
    lmres <- matrix(vector("list", length(sites) * length(conditions)), nrow = length(sites))
    for (i in seq_along(sites)) {
        onesite <- dta_summod %>% filter(site == sites[i])
        for (j in seq_along(conditions)) {
            if (conditions[j] %in% onesite$cond) {
                lmres[[i, j]] <- onesite %>%
                    filter(cond == conditions[j]) %>%
                    complete(nesting(site, cond, run), form, fill = list(inty_form = 0)) %>%
                    group_by(run) %>%
                    mutate(inty_sum = sum(inty_form, na.rm = TRUE), occ = inty_form / inty_sum * 100) %
                    group_by(site, cond, form) %>%
                    summarise(occ_est = mean(occ), occ_sd = sd(occ), nb_run = n(),
                              occ_se = occ_sd / sqrt(nb_run)) %>%
                    ungroup() %>%
                    select(site, cond, form, nb_run, occ_est, occ_se)
```

```r
            }
        }
    }
    rownames(lmres) <- sites
    colnames(lmres) <- conditions

    return(lmres)
}


## modeling on the scale of log2-intensity
model_log2inty <- function(dta_summod, conditions, sites) {
    dta_summod <- dta_summod %>% filter(site %in% sites, cond %in% conditions)
    nb_reprun <- dta_summod %>% group_by(site, cond, form) %>%
        summarise(nb_run = n_distinct(run)) %>% ungroup()
    ## remove singletons (that inhibit inference)
    dta_summod <- dta_summod %>%
        inner_join(nb_reprun) %>%
        filter(nb_run > 1)
    lmres <- matrix(vector("list", length(sites) * length(conditions)), nrow = length(sites))
    for (i in seq_along(sites)) {
        onesite <- dta_summod %>% filter(site == sites[i])
        for (j in seq_along(conditions)) {
            lmres[[i, j]] <- onesite %>%
                filter(cond == conditions[j]) %>%
                group_by(site, cond, form) %>%
                summarise(logabun_est = mean(log2inty), logabun_sd = sd(log2inty),
                          nb_run = n(), logabun_se = logabun_sd / sqrt(nb_run)) %>%
                ungroup() %>%
                select(site, cond, form, nb_run, logabun_est, logabun_se)
        }
    }
    rownames(lmres) <- sites
    colnames(lmres) <- conditions

    return(lmres)
}


## estimate site occupancy using the fitted models
est_siteocc <- function(lmres, sumscale = "abundance", conf_level = 0.95) {
    t_alpha <- (1 - conf_level) / 2
    soeform <- matrix(vector("list", nrow(lmres) * ncol(lmres)), nrow = nrow(lmres))
    if (sumscale == "abundance") {
        for (i in 1:nrow(lmres)) {
            for (j in 1:ncol(lmres)) {
                onecond <- lmres[[i, j]]
                soeform[[i, j]] <- onecond %>%
                    mutate(occ_est = 100 * abun_rel, occ_se = 100 * abun_rel_se,
                           occ_df = ifelse(df_den == 0 | df_den == Inf, 0, df_num / df_den)) %>%
                    rowwise() %>%
                    mutate(occ_me = ifelse(occ_df == 0, NA_real_, qt(1 - t_alpha, occ_df) * occ_se)) %>[illegible]
                    ungroup() %>%
```

```
                    mutate(occ_lb = occ_est - occ_me,
                           occ_lb = ifelse(occ_lb < 0, 0, occ_lb),
                           occ_ub = occ_est + occ_me,
                           occ_ub = ifelse(occ_ub > 100, 100, occ_ub)) %>%
                    select(site, cond, form, occ_est, occ_se, occ_lb, occ_ub)
            }
        }
    } else if (sumscale == "occrun") {
        for (i in 1:nrow(lmres)) {
            for (j in 1:ncol(lmres)) {
                onecond <- lmres[[i, j]]
                if (!is.null(onecond)) {
                    soeform[[i, j]] <- onecond %>%
                        mutate(occ_df = nb_run - 1,
                               occ_me = qt(1 - t_alpha, occ_df) * occ_se,
                               occ_lb = occ_est - occ_me,
                               occ_lb = ifelse(occ_lb < 0, 0, occ_lb),
                               occ_ub = occ_est + occ_me,
                               occ_ub = ifelse(occ_ub > 100, 100, occ_ub)) %>%
                        select(site, cond, form, occ_est, occ_se, occ_lb, occ_ub)
                }
            }
        }
    }

    return(bind_rows(soeform))
}


## test for differential site occupancy using the fitted models
test_diffocc <- function(lmres, conditions, idx_ctrl, idx_case, sumscale = "abundance") {
    dsoform <- matrix(vector("list", nrow(lmres) * length(idx_case)), nrow = nrow(lmres))
    if (sumscale == "abundance") {
        for (i in 1:nrow(lmres)) {
            for (j in seq_along(idx_case)) {
                j0 <- idx_ctrl[j]
                j1 <- idx_case[j]
                twocond <- bind_rows(lmres[i, c(j0, j1)])
                if (nrow(twocond) > 0) {
                    dsoform[[i, j]] <- twocond %>%
                        mutate(cond = factor(cond, levels = conditions[c(j0, j1)])) %>%
                        complete(site, cond, form, fill = list(abun_rel = -Inf)) %>%
                        group_by(site, form) %>%
                        summarise(dso_est = 100 * diff(abun_rel),
                                  dso_se = 100 * sqrt(sum(abun_rel_se ^ 2)),
                                  dso_df = ifelse(dso_se == 0, 0, sum(sqrt(df_num)) ^ 2 / sum(df_den)))
                        ungroup() %>%
                        mutate(t_stat = ifelse(dso_se == 0, NA_real_, dso_est / dso_se),
                               p_val = ifelse(abs(dso_est) == Inf, 0,
                                              ifelse(dso_df == 0, NA, 2 * pt(abs(t_stat), df = dso_df, [illegible]
                               control = conditions[j0], case = conditions[j1]) %>%
                        select(site, form, control, case, dso_est, dso_se, dso_df, t_stat, p_val)
                }
```

```
            }
        }
    } else if (sumscale == "occrun") {
        for (i in 1:nrow(lmres)) {
            for (j in seq_along(idx_case)) {
                j0 <- idx_ctrl[j]
                j1 <- idx_case[j]
                twocond <- bind_rows(lmres[i, c(j0, j1)])
                if (nrow(twocond) > 0) {
                    dsoform[[i, j]] <- twocond %>%
                        mutate(cond = factor(cond, levels = conditions[c(j0, j1)])) %>%
                        complete(site, cond, form, fill = list(occ_est = -Inf)) %>%
                        mutate(occ_se2 = occ_se ^ 2) %>%
                        group_by(site, form) %>%
                        summarise(dso_est = diff(occ_est),
                                  dso_se = sqrt(sum(occ_se2)),
                                  dso_df = ifelse(dso_se == 0, 0, sum(occ_se2) ^ 2 / sum(occ_se2 ^ 2 /
                        ungroup() %>%
                        mutate(t_stat = ifelse(dso_se == 0, NA_real_, dso_est / dso_se),
                               p_val = ifelse(abs(dso_est) == Inf, 0,
                                              ifelse(dso_df == 0, NA, 2 * pt(abs(t_stat), df = dso_df,
                               control = conditions[j0], case = conditions[j1]) %>%
                        select(site, form, control, case, dso_est, dso_se, dso_df, t_stat, p_val)
                }
            }
        }
    }
    dsores <- bind_rows(dsoform)
    to_adj <- !is.na(dsores$t_stat)
    dsores$adj_p_val <- dsores$p_val
    dsores$adj_p_val[to_adj] <- p.adjust(dsores$p_val[to_adj], method = "BH")

    return(dsores)
}


test_diffabun <- function(lmres, conditions, idx_ctrl, idx_case) {
    dsaform <- matrix(vector("list", nrow(lmres) * length(idx_case)), nrow = nrow(lmres))
    for (i in 1:nrow(lmres)) {
        for (j in seq_along(idx_case)) {
            j0 <- idx_ctrl[j]
            j1 <- idx_case[j]
            twocond <- bind_rows(lmres[i, c(j0, j1)])
            if (nrow(twocond) > 0) {
                dsaform[[i, j]] <- twocond %>%
                    mutate(cond = factor(cond, levels = conditions[c(j0, j1)])) %>%
                    complete(site, cond, form, fill = list(logabun_est = -Inf)) %>%
                    mutate(logabun_se2 = logabun_se ^ 2) %>%
                    group_by(site, form) %>%
                    summarise(dsa_est = diff(logabun_est),
                              dsa_se = sqrt(sum(logabun_se2)),
                              dsa_df = ifelse(dsa_se == 0, 0, sum(logabun_se2) ^ 2 / sum(logabun_se2 ^
                    ungroup() %>%
```

```r
                    mutate(t_stat = ifelse(dsa_se == 0, NA_real_, dsa_est / dsa_se),
                           p_val = ifelse(abs(dsa_est) == Inf, 0,
                                          ifelse(dsa_df == 0, NA, 2 * pt(abs(t_stat), df = dsa_df, lowe
                           control = conditions[j0], case = conditions[j1]) %>%
                    select(site, form, control, case, dsa_est, dsa_se, dsa_df, t_stat, p_val)
            }
        }
    }
    dsares <- bind_rows(dsaform)
    to_adj <- !is.na(dsares$t_stat)
    dsares$adj_p_val <- dsares$p_val
    dsares$adj_p_val[to_adj] <- p.adjust(dsares$p_val[to_adj], method = "BH")

    return(dsares)

}


## estimate combined occupancy
est_combocc <- function(dta_summod, conditions, sites_list, ptnform, modgroup,
                        sumscale = "abundance", conf_level = 0.95) {
    if (sumscale == "occrun") {
        return(est_combocc_run(dta_summod, conditions, sites_list, ptnform, modgroup, conf_level))
    }
    t_alpha <- (1 - conf_level) / 2
    sites = unlist(sites_list, use.names = FALSE)
    ## level: "c" (condition)
    dta_summod <- dta_summod %>% filter(site %in% sites, cond %in% conditions)
    nb_rep <- dta_summod %>% group_by(site, cond, form) %>%
        summarise(nb_run = n_distinct(run)) %>% ungroup()
    ## fit linear models and combine the results
    df_form <- dta_summod %>%
        group_by(site, form, cond) %>%
        do(tidy(lm(log2inty ~ 1, data = .))) %>%
        ungroup() %>%
        select(site, cond, form, log2abun_est = estimate, log2abun_se = std.error)
    ## remove singletons (that inhibit inference)
    df_form <- df_form %>%
        mutate(abun = 2 ^ log2abun_est) %>%
        inner_join(nb_rep) %>%
        filter(nb_run > 1)
    ## map forms to modification
    tmpmod <- vector("character", nrow(df_form))
    for (m in seq_along(modgroup)) {
        tmpmod[grepl(pattern = ptnform[m], df_form$form)] <- modgroup[m]
    }
    df_form$mod <- tmpmod
    ## estimate combined occupancy using the fitted models
    coe <- matrix(vector("list", length(sites_list) * length(conditions)), nrow = length(sites_list))
    for (i in seq_along(sites_list)) {
        onecomb <- df_form %>% filter(site %in% sites_list[[i]])
        csite <- paste(sites_list[[i]], collapse = "-")
        for (j in seq_along(conditions)) {
```

```
            onecond <- onecomb %>% filter(cond == conditions[j]) %>%
                mutate(abun_sum = sum(abun), abun_rel = abun / abun_sum, abun_relc = 1 - abun_rel)
            ## gradient of site occupancy in the following 3 lines
            occ_grad <- -crossprod(t(onecond$abun_rel))
            diag(occ_grad) <- onecond$abun_rel * onecond$abun_relc
            occ_grad <- log(2) * occ_grad
            ## calculate the SE and DF using the Delta method
            logabun_se <- onecond$log2abun_se
            combrel_est <- combrel_se <- combrel_df <- rep(NA, length(modgroup))
            for (m in seq_along(modgroup)) {
                is_combform <- (onecond$mod == modgroup[m])
                comb_grad <- rowSums(occ_grad[, is_combform, drop = F])
                combrel_est[m] <- sum(onecond$abun_rel[is_combform])
                combrel_se[m] <- sqrt(sum((comb_grad * logabun_se) ^ 2))
                combrel_df[m] <- combrel_se[m] ^ 4 / sum((comb_grad * logabun_se) ^ 4 / (onecond$nb_run
            }
            onemod <- data_frame(combsite = csite, cond = conditions[j], mod = modgroup,
                                 occ_est = combrel_est * 100, occ_se = combrel_se * 100,
                                 occ_df = combrel_df)
            coe[[i, j]] <- onemod %>% rowwise() %>%
                mutate(occ_me = ifelse(occ_df == 0, NA_real_, qt(1 - t_alpha, occ_df) * occ_se)) %>%
                ungroup() %>%
                mutate(occ_lb = occ_est - occ_me, occ_lb = ifelse(occ_lb < 0, 0, occ_lb),
                       occ_ub = occ_est + occ_me, occ_ub = ifelse(occ_ub > 100, 100, occ_ub)) %>%
                select(combsite, cond, mod, occ_est, occ_se, occ_df, occ_lb, occ_ub)
        }
    }

    return(bind_rows(coe))
}


est_combocc_run <- function(dta_summod, conditions, sites_list, ptnform, modgroup, conf_level = 0.95) {
    t_alpha <- (1 - conf_level) / 2
    sites = unlist(sites_list, use.names = FALSE)
    dta_summod <- dta_summod %>%
        filter(site %in% sites, cond %in% conditions) %>%
        mutate(inty_form = 2 ^ log2inty)
    ## map forms to modification
    tmpmod <- vector("character", nrow(dta_summod))
    for (m in seq_along(modgroup)) {
        tmpmod[grepl(pattern = ptnform[m], dta_summod$form)] <- modgroup[m]
    }
    dta_summod$mod <- tmpmod
    ## estimate the combined occupancy with run-level summary
    coe <- matrix(vector("list", length(sites_list) * length(conditions)), nrow = length(sites_list))
    for (i in seq_along(sites_list)) {
        onecomb <- dta_summod %>% filter(site %in% sites_list[[i]]) %>%
            mutate(combsite = paste(sites_list[[i]], collapse = "-"))
        nb_reprun <- onecomb %>% group_by(cond) %>%
            summarise(nb_run = n_distinct(run)) %>% ungroup()
        onecomb <- inner_join(onecomb, nb_reprun) %>% filter(nb_run > 1)
        for (j in seq_along(conditions)) {
```

```
            onemod <- onecomb %>%
                filter(cond == conditions[j]) %>%
                group_by(combsite, cond, run, mod) %>%
                summarise(inty_mod = sum(inty_form, na.rm = TRUE)) %>%
                ungroup() %>%
                complete(nesting(combsite, cond, run), mod, fill = list(inty_mod = 0)) %>%
                group_by(run) %>%
                mutate(inty_sum = sum(inty_mod, na.rm = TRUE), occ = inty_mod / inty_sum * 100) %>%
                group_by(combsite, cond, mod) %>%
                summarise(occ_est = mean(occ), occ_sd = sd(occ), nb_run = n(),
                          occ_se = occ_sd / sqrt(nb_run)) %>%
                ungroup()
            coe[[i, j]] <- onemod %>%
                mutate(occ_df = nb_run - 1,
                       occ_me = qt(1 - t_alpha, occ_df) * occ_se,
                       occ_lb = occ_est - occ_me, occ_lb = ifelse(occ_lb < 0, 0, occ_lb),
                       occ_ub = occ_est + occ_me, occ_ub = ifelse(occ_ub > 100, 100, occ_ub)) %>%
                select(combsite, cond, mod, occ_est, occ_se, occ_df, occ_lb, occ_ub)
        }
    }

    return(bind_rows(coe))
}


## visualization ----------------------------------------------------
## profile plots of features in forms
plot_pfform <- function(dta, ss) {
    idx_cumsum <- dta %>% distinct(cond, run) %>% count(cond) %>% .$n %>% cumsum()
    dta_tmp <- dta %>% filter(site == ss) %>% select(-site) %>% rowwise() %>%
        mutate(pos_pep = unlist(strsplit(feature, "__"))[1],
               z_iso = unlist(strsplit(feature, "__"))[2],
               chrg_prec = unlist(strsplit(gsub("z", "", z_iso), "_"))[1],
               iso_idx = unlist(strsplit(gsub("z", "", z_iso), "_"))[2]) %>%
        ungroup() %>%
        mutate(pz = paste0(pos_pep, "_", "z", chrg_prec))
    dta_ctmp <- dta_tmp %>% select(feature, pz, run, form, log2inty) %>%
        complete(nesting(feature, pz), run, form, fill = list(log2inty = 0))
    gp_pfform <- ggplot(dta_ctmp, aes(run, log2inty, group = feature, colour = factor(pz)))
    gp_pfform + geom_point() +
        geom_line() + facet_wrap(~ form, ncol = 3) +
        geom_vline(xintercept = 0.5 + idx_cumsum[-length(idx_cumsum)], linetype = "dashed", colour = "g[illegible]
        xlab("Run") + ylab("Log2-intensity") +
        coord_cartesian(ylim = c(0, 32)) +
        theme_bw() +
        theme(axis.text.x = element_text(angle = 90, hjust = 1)) +
        theme(legend.position = "bottom", legend.box = "horizontal") +
        scale_colour_discrete(name ="Peptide ions")
}


## profile plots of features with summarization in forms
plot_sumform <- function(dta, ss) {
```

```
    idx_cumsum <- dta %>% distinct(cond, run) %>% count(cond) %>% .$n %>% cumsum()
    dta_ftr0 <- dta %>% filter(site == ss, quan == "feature") %>%
        complete(feature, run, form, quan, fill = list(log2inty = 0))
    dta_ftrsum0 <- dta %>% filter(site == ss, quan == "summarization") %>%
        complete(feature, run, form, quan, fill = list(log2inty = 0)) %>%
        bind_rows(dta_ftr0)
    gp_sum <- ggplot(dta_ftrsum0, aes(run, log2inty, group = feature, colour = factor(quan), size = fac
    gp_sum + geom_point() + geom_line(size=0.5) + facet_wrap(~ form, ncol = 3) +
        scale_colour_manual(values = c("lightgray", "darkred")) +
        scale_size_manual(values = c(1, 2)) +
        geom_vline(xintercept = 0.5 + idx_cumsum[-length(idx_cumsum)], linetype = "dashed", colour = "g
        xlab("Run") + ylab("Log2-intensity") +
        coord_cartesian(ylim = c(0, 32)) +
        theme_bw() +
        theme(axis.text.x = element_text(angle = 90, hjust = 1)) +
        theme(legend.title = element_blank(), legend.position = "bottom", legend.box = "horizontal")
}


## volcano plots
plot_volcano <- function(dta, sig_level = 0.05, plim = Inf, dlim = 50, gname = NULL) {
    data_dso <- dta %>%
        mutate(nlog_pval = -log10(adj_p_val),
               form = ifelse(nlog_pval > plim, paste0(form, "*"), form),
               nlog_pval = ifelse(nlog_pval > plim, plim, nlog_pval),
               col_sig = ifelse(adj_p_val > sig_level, "nonsig", ifelse(dso_est > 0, "upreg", "dnreg"))
    vp_tmp <- ggplot(data_dso, aes(dso_est, nlog_pval, colour = col_sig)) +
        geom_point(size = 4, alpha = 0.8) +
        scale_colour_manual(name = NULL, values = c("gray65", "blue", "red"),
                            limits = c("nonsig", "dnreg", "upreg"),
                            labels = c("No change", "Decreased occupancy", "Increased occupancy")) +
        xlab("Difference in site occupancy (%)") + ylab("-log10 [adjusted p-value]") + ggtitle(gname) +
        geom_hline(aes(yintercept = -log10(sig_level)), colour = "gray20", linetype = 2) +
        scale_x_continuous(limits = c(-dlim, dlim)) +
        scale_y_continuous(limits = c(0, plim)) +
        theme_bw() +
        theme(legend.position = "none") +
        theme(axis.text = element_text(size = 10),
              axis.title = element_text(size = 14),
              plot.title = element_text(size = 16, face = "bold"),
              strip.text.x = element_text(size = 14))
    vp_tmp + geom_text_repel(data = filter(data_dso, adj_p_val <= sig_level), aes(label = form))
}
```

Session information

```
sessionInfo()
```

```
## R version 3.3.0 (2016-05-03)
## Platform: x86_64-apple-darwin13.4.0 (64-bit)
## Running under: OS X 10.12.5 (unknown)
##
```

```
## locale:
## [1] en_US.UTF-8/en_US.UTF-8/en_US.UTF-8/C/en_US.UTF-8/en_US.UTF-8
##
## attached base packages:
## [1] stats     graphics  grDevices utils     datasets  methods   base
##
## other attached packages:
## [1] ggrepel_0.6.5   ggplot2_2.2.1   survival_2.41-3 broom_0.4.2
## [5] dplyr_0.5.0     tidyr_0.6.1     readr_1.1.0
##
## loaded via a namespace (and not attached):
##  [1] Rcpp_0.12.10     plyr_1.8.4       tools_3.3.0      digest_0.6.12
##  [5] evaluate_0.10    tibble_1.3.0     nlme_3.1-131     gtable_0.2.0
##  [9] lattice_0.20-35  Matrix_1.2-8     psych_1.7.3.21   DBI_0.6-1
## [13] yaml_2.1.14      parallel_3.3.0   stringr_1.2.0    knitr_1.15.1
## [17] hms_0.3          rprojroot_1.2    grid_3.3.0       R6_2.2.0
## [21] foreign_0.8-67   rmarkdown_1.4    reshape2_1.4.2   magrittr_1.5
## [25] backports_1.0.5  scales_0.4.1     htmltools_0.3.5  splines_3.3.0
## [29] assertthat_0.2.0 mnormt_1.5-5     colorspace_1.3-2 labeling_0.3
## [33] stringi_1.1.5    lazyeval_0.2.0   munsell_0.4.3
```
